# Whole-Blood Fatty Acid Phenotypes and Subsequent Quality-of-Life Changes Among Cancer Survivors

**DOI:** 10.3390/nu18172838

**Published:** 2026-08-28

**Authors:** Lin Lv, Juan Xia, Xinyu Zhu, Luxin Wang, Chen Lv, Yanyi Fu, Donghui Yu, Jinming Yu

**Affiliations:** 1 School of Public Health, Fudan University, Shanghai 200032, China; 2 School of Public Health, Capital Medical University, Beijing 100069, China; 3 National Clinical Research Center for Eye Diseases, Shanghai General Hospital, Shanghai 200080, China

**Keywords:** whole-blood fatty acids, nutritional-metabolic phenotype, cancer survivors, quality of life, Diet Balance Index 2022, longitudinal cohort

## Abstract

**Background/Objectives:** Whole-blood fatty acid composition provides an objective biomarker of fatty acid status, but its relationships with nutritional characteristics and subsequent quality-of-life (QoL) changes among cancer survivors remain incompletely understood. We aimed to identify whole-blood fatty acid phenotypes and examine their associations with nutritional characteristics, cross-sectional QoL profiles, and subsequent QoL changes in an overlapping longitudinal subcohort. **Methods:** In this cohort study, the 2023 baseline investigation included 3175 community-dwelling adult cancer survivors with whole-blood fatty acid profiles and paired questionnaires. After excluding participants with substantial missing QoL data, implausible fatty acid measurements, or missing age, sex, or cancer type, 3009 participants comprised the baseline analytical sample. Among these, 423 overlapped with an independent longitudinal cohort contributing QoL data from June 2021 and June 2024, and 398 had complete covariate data for the core adjusted longitudinal analyses. Whole-blood fatty acid phenotypes were identified using principal component analysis and unsupervised clustering. Nutritional characteristics were assessed using habitual dietary intake, Diet Balance Index 2022 scores, and nutrition literacy measures. QoL was assessed using the EORTC QLQ-C30. Multivariable models examined cross-sectional QoL and annualised QoL changes. An open-source phenotype assignment tool was developed to support reproducible classification in future cohorts. **Results:** Among 3009 baseline participants (mean [SD] age, 64.9 [7.0] years; 2397 women [79.7%]), five whole-blood fatty acid phenotypes were identified. Phenotypes 2 and 3 were rarely observed in the available general-population reference sample and appeared enriched among survivors. Phenotype 2, a high-SFA/low-PUFA phenotype accompanied by higher red meat intake and greater DBI-22 excess scores for red meat and related animal foods, was associated with statistically significant worsening across multiple QoL domains over 12 months despite relatively favourable baseline QoL. Compared with the general-population-mapped reference pool (Phenotypes 1, 4, and 5), Phenotype 2 showed annualised declines in summary score (−4.66 [95% CI, −8.14 to −1.19]) and role functioning (−5.35 [−9.88 to −0.83]), and worsening pain (8.28 [2.58 to 13.98]), dyspnoea (6.83 [0.16 to 13.50]), and constipation (14.10 [7.30 to 20.90]). Prebaseline annualised QoL changes over the preceding 24 months did not differ significantly across phenotypes. **Conclusions:** Whole-blood fatty acid profiling identified distinct nutritional-metabolic phenotypes among community-dwelling cancer survivors. The high-SFA/low-PUFA phenotype, accompanied by higher red meat intake and greater DBI-22 excess scores for red meat and related animal foods, was associated with worsening across multiple QoL domains over the subsequent 12 months despite relatively favourable baseline QoL. These findings highlight whole-blood fatty acid phenotyping as a potentially informative framework for characterising nutritional-metabolic heterogeneity and subsequent QoL vulnerability among cancer survivors. The open-source phenotype assignment tool may facilitate reproducible phenotype assignment and external validation in future cohorts.

## 1. Introduction

Cancer remains a major global public health challenge. Advances in screening and treatment have prolonged survival, leading to a rapidly expanding population of cancer survivors; as of 2022, more than 50 million individuals worldwide were living within 5 years after diagnosis [1]. In China, an estimated 5.15 million new cancer cases and 2.58 million cancer deaths occurred in 2024 [2]. Consequently, survivorship care has shifted from acute treatment to long-term management, rehabilitation, and maintenance of quality of life (QoL) [3]. Multidimensional QoL has therefore become an important patient-reported outcome for evaluating recovery, symptom burden, and ongoing health needs among cancer survivors [4]. In this setting, approaches that characterise nutritional-metabolic heterogeneity before overt functional decline or symptom worsening becomes apparent may help clarify variation in subsequent survivorship outcomes.

Metabolic alterations can persist after completion of cancer treatment and could contribute to long-term health outcomes [5]. Lipid metabolism is a central component of cancer biology [6,7], and fatty acids are involved in inflammatory signalling, membrane structure, cellular signalling, and neurocognitive processes [8,9]. These functions make fatty acid patterns relevant to symptoms, functional status, and survivorship outcomes, beyond their traditional role as markers of dietary intake.

Fatty acid exposure in epidemiological studies is commonly assessed using self-reported dietary intake or circulating biomarkers such as plasma fatty acids. Dietary questionnaires are susceptible to recall and reporting bias, whereas circulating fatty acid measurements provide an objective assessment of fatty acid status. Fasting whole-blood fatty acid composition captures fatty acids across circulating cellular and plasma lipid pools and has been shown to reflect habitual intake of selected essential fatty acids with performance comparable to fasting plasma [10]. Whole-blood dried blood spot (DBS) sampling also offers practical advantages, including minimally invasive finger-prick collection, low blood-volume requirements, and minimal sample processing, making it well suited to large population-based and community-based studies [11]. Standardised fasting collection can further reduce variability related to recent food intake [12].

Although lipid metabolism and fatty acid status have been widely studied in relation to tumourigenesis, treatment response, and cancer progression, emerging survivorship studies have linked erythrocyte *n*-3 fatty acids with body composition and physical function in breast cancer, red-blood-cell polyunsaturated fatty acids with mortality after breast cancer, and circulating metabolic profiles with persistent fatigue after colorectal cancer treatment [13,14,15]. However, the broader literature has largely focused on individual fatty acids in relation to cancer development and progression, while survivorship studies remain limited to selected biomarkers and cancer types; the relationships of multidimensional whole-blood fatty acid phenotypes with nutritional characteristics and subsequent QoL changes among cancer survivors therefore remain unclear. This gap is particularly relevant to community-based survivorship care, where single-time-point dietary or QoL assessments may provide an incomplete view of later deterioration in functioning or symptoms. Because circulating fatty acids are intercorrelated and jointly reflect dietary and endogenous metabolic influences, a phenotype-level approach may help characterise broader nutritional-metabolic states that are not captured by individual fatty acids alone.

In this cohort study, we aimed to identify whole-blood fatty acid phenotypes and examine their associations with nutritional characteristics, cross-sectional QoL profiles, and subsequent QoL changes in an overlapping longitudinal subcohort. We identified whole-blood fatty acid phenotypes in a large city-wide community-based cohort of cancer survivors, characterised phenotype-specific nutritional profiles using habitual dietary intake, Diet Balance Index 2022 scores, and nutrition literacy measures, examined cross-sectional associations with QoL outcomes in the full baseline cohort, and assessed subsequent QoL changes in the overlapping longitudinal subcohort. We also evaluated the feasibility of projecting independent external samples into the established phenotype space and developed an open-source assignment tool to support reproducible phenotype assignment and future external validation.

## 2. Materials and Methods

### 2.1. Study Design, Setting, and Ethics

Participants were recruited from the Shanghai Cancer Recovery Club, a city-wide, community-based nongovernmental organization comprising branches across all administrative districts of Shanghai [16]. Approvals for the baseline cross-sectional investigation were obtained from Fudan University (IRB No. 2020-12-0860-S) and Capital Medical University (IRB No. Z2022SY002). The independent longitudinal cohort was approved by Fudan University (IRB No. 2021-02-0877). All participants provided written informed consent. The study followed the STROBE reporting guideline [17].

### 2.2. Study Population

Eligible participants were adult cancer survivors (≥18 years) who had completed active antineoplastic treatment, were living in the community, and were able to complete questionnaire-based assessments. The baseline cross-sectional investigation was conducted from March to September 2023 and initially included 3175 participants with whole-blood fatty acid profiles and paired questionnaires, completed either through face-to-face interviews or self-administration. After exclusion of 52 participants with ≥10 missing EORTC QLQ-C30 items, 1 participant with an implausible fatty acid measurement (negative value), and 113 participants with missing age, sex, or cancer type, 3009 participants comprised the final baseline analytical sample (Figure S1).

The 3009 participants in the baseline analytical sample were cross-linked with an independent longitudinal cohort (*n* = 1051) established under a separate study protocol using participant identifiers. This linkage identified 423 overlapping participants, who constituted the longitudinal subcohort; the remaining 2586 participants contributed exclusively to the cross-sectional cohort. For these participants, the 2023 whole-blood fatty acid assessment served as the metabolic anchor. QoL assessed at the 2023 baseline survey was combined with QoL data from two additional survey waves conducted in June 2021 and June 2024, corresponding approximately to 24 months before and 12 months after the fatty acid assessment. These data were used to characterise prebaseline QoL change and prospective postbaseline QoL change, respectively.

### 2.3. Whole-Blood Fatty Acid Profiling

Fasting capillary whole blood was collected by finger prick in the morning and applied directly to dried blood spot (DBS) cards (Omegabandz, Shanghai, China). The DBS samples were stored at 5 °C and analysed within 1 week of collection. Fatty acids from a standardised punch of each DBS card were converted to fatty acid methyl esters (FAMEs) by direct transesterification and analysed by gas chromatography using an Agilent 7820A system (Agilent Technologies, Santa Clara, CA, USA) equipped with a DB-23 capillary column (60 m; Agilent Technologies, Santa Clara, CA, USA). Fatty acids were identified and quantified based on retention times and peak areas of a commercial FAME reference mixture (Nu-Chek-Prep, Inc., Waterville, MN, USA), which was analysed with each sample batch for quality control. Results were expressed as relative percentages of the total identified whole-blood fatty acids (Methods S1) [18,19].

Among the 37 initially identified fatty acids, 4 trace short- and medium-chain fatty acids (C10:0, C11:0, C12:0, and C13:0; cumulatively < 0.1%) were excluded a priori because of suboptimal quantification reliability. Eicosadienoic acid (20:2) and trans fatty acids were analysed individually and excluded from the summations of major fatty acid classes.

### 2.4. Dietary Assessment, DBI-22 Scoring, and Nutrition Literacy

Habitual dietary intake and the derived Diet Balance Index 2022 (DBI-22) [20] were evaluated via a 20-item semi-quantitative food frequency questionnaire (SQFFQ) (Methods S2; Table S1). The DBI-22 summarises both insufficient and excessive intake across major food groups: the Low Bound Score (LBS) reflects dietary inadequacy, the High Bound Score (HBS) reflects dietary excess, and the Diet Quality Distance (DQD) reflects overall dietary imbalance. Energy intake and cooked-to-raw conversions were calculated using China Food Composition Tables coefficients [21]. Nutrition literacy was measured using a validated scale [22,23].

### 2.5. Quality-of-Life Assessment

The outcomes were quality of life (QoL) and annualised QoL change, assessed using the EORTC QLQ-C30 (version 3.0) [24]. This 30-item instrument includes 5 functional scales (physical, role, emotional, cognitive, and social functioning), 9 symptom domains (fatigue, nausea and vomiting, pain, dyspnoea, insomnia, appetite loss, constipation, diarrhoea, and financial difficulties), global health status, and the EORTC QLQ-C30 summary score. Responses were linearly transformed to a standardised 0-to-100 scale. Higher functional, global health, and summary scores indicate better QoL or functioning, whereas higher symptom scores indicate greater symptom burden. The EORTC QLQ-C30 summary score was treated as the primary QoL outcome, whereas individual functional and symptom domains were analysed as secondary exploratory outcomes; no formal multiplicity adjustment was applied across QoL domains.

Annualised QoL change was calculated as the difference between the later and earlier QoL assessments divided by the interval in years. For the prebaseline period, change was calculated as the 2023 baseline score minus the 2021 score; for the postbaseline period, change was calculated as the 2024 follow-up score minus the 2023 baseline score. Interpretation followed the EORTC QLQ-C30 scoring direction: positive changes indicate improvement for functional, global health, and summary scores, whereas positive changes in symptom scores indicate greater symptom burden.

To aid interpretation of effect magnitude, cross-sectional between-group differences in EORTC QLQ-C30 scores and between-group differences in annualised score changes were contextualised using published minimally important difference (MID) guidance. A 5-point difference, representing the lower bound of the commonly used 5–10-point range for clinically important group-level differences or changes, was used as a descriptive benchmark for contextual interpretation [25,26]. For the EORTC QLQ-C30 summary score, the 5-point benchmark was used for contextual interpretation because the cited MID guidance did not derive a summary-score-specific MID.

### 2.6. Phenotype Identification and External Projection

Given the compositional nature of the whole-blood fatty acid data, 4 specific fatty acids with >50% zero values (C21:0, C14:1, C20:3n-3, and C22:2n-6) were excluded prior to centred log-ratio (CLR) transformation [27]. To account for the intrinsic intercorrelations among the remaining fatty acids, the transformed relative abundances underwent principal component analysis (PCA), with the first seven components retained based on scree plot inspection. Unsupervised K-means clustering, informed by the gap statistic, was used to identify whole-blood fatty acid phenotypes [28]. Cluster stability was assessed using 1000 bootstrap resamples of the retained PCA scores, with five-cluster K-means clustering refitted in each resample and cluster-wise stability quantified using mean Jaccard similarity. The qualitative phenotype descriptors (high, moderate, low, and intermediate) were assigned post hoc to summarise relative differences in fatty acid composition within the cancer-survivor cohort and did not represent clinically defined thresholds.

The feasibility of external phenotype projection was evaluated by projecting independent external samples into the established PCA space. The available general-population reference sample and external survivor samples were likewise based on whole-blood fatty acid profiles generated by the same laboratory. Based on the external projection results, Phenotypes 1, 4, and 5 were pooled as the general-population-mapped reference pool for downstream contrasts within the cancer-survivor cohort because they collectively represented nearly all classified profiles in the available general-population reference sample. This pooling provided a common analytical comparator and did not imply metabolic homogeneity or clinical normality.

### 2.7. Open-Source Phenotype Assignment Tool

To support reproducible phenotype assignment in future cohorts, we developed an open-source R-based Whole-Blood Fatty Acid Phenotype Calculator (version 1.0.0). This phenotype assignment tool applies batch-adaptive zero imputation, projects external whole-blood fatty acid profiles into the fixed baseline PCA space, and assigns profiles to the nearest phenotype only when the multidimensional distance falls within the prespecified 99th percentile intracluster boundary; otherwise, profiles are flagged as unclassified outliers. The executable software, example validation dataset, and operational instructions are available in a public GitHub version 1.0.0 repository and described in Supplementary File S2; the detailed mathematical framework is provided in Methods S3.

### 2.8. Statistical Analysis

Observations with ≥10 missing EORTC QLQ-C30 items, any negative fatty acid quantifications, or missing core demographic data were excluded, yielding a baseline sample of 3009 (including 423 overlapping participants). Dietary analyses formally excluded participants with >3 missing SQFFQ items or biologically implausible daily energy intakes [29].

Baseline data were expressed as means (SDs) or frequencies (percentages). Categorical characteristics were compared using chi-square or Fisher exact tests. Continuous variables were compared using independent-samples *t* tests, ANOVA, Wilcoxon rank-sum, or Kruskal–Wallis tests. Dietary comparisons used hierarchical adjustments: DBI indices for cancer type; total energy and dietary variety for age, sex, and cancer type; individual components further adjusted for total energy.

Informed by comparisons of baseline characteristics across phenotypes and between the overlapping longitudinal subcohort and the exclusively cross-sectional participants, 3 progressive multivariable models were constructed. Model 1 adjusted for age, sex, and cancer type. Model 2 additionally adjusted for body mass index, income, smoking, drinking, living arrangement, physical activity, comorbidities, survivorship duration, and disease trajectory. Model 3 further incorporated COVID-19 severity. Education was excluded due to high missingness and collinearity with income. Model 2 served as the core adjusted multivariable model. Because the primary analyses used a complete-case approach, the number of participants varied according to covariate availability. For the baseline cross-sectional analyses, Models 1, 2, and 3 included 3009, 2698, and 2679 participants, respectively. For the longitudinal analyses, the corresponding sample sizes were 423, 398, and 395 participants. Thus, the core adjusted Model 2 included 2698 participants in the cross-sectional analyses and 398 participants in the longitudinal analyses. Annualised change analyses additionally adjusted for the QoL score at the start of each corresponding interval to account for differences in initial QoL levels and reduce potential regression-to-the-mean effects.

Phenotypic differences in QoL and its annualised changes were evaluated using multivariable type II ANCOVA, or rank-transformed ANCOVA when model assumptions were not met. To compare Phenotypes 2 and 3 with the general-population-mapped reference pool, contrasts based on estimated marginal means were performed. Repeated QoL measurements across the available survey waves were modelled using linear mixed models (LMMs). To address missing covariates, sensitivity analyses employed multiple imputation by chained equations (MICE), determining global significance via pooled F statistics (D1) and synthesising contrasts using Rubin rules [30]. Additional sensitivity analyses assessed the robustness of the prospective phenotype-QoL associations to measured dietary characteristics and cohort composition. Among participants meeting the dietary-data quality criteria, the core Model 2 was first refitted in the same analytical sample and was then additionally adjusted for total energy intake and intake of beef, mutton, and other red meats. Restricted-sample analyses repeated the core Model 2 after excluding breast cancer survivors, among women only, and among survivors 5–10 years after diagnosis. Covariates rendered invariant by a restriction, as well as any other covariate with no within-stratum variation, were omitted from the corresponding restricted model; all other Model 2 covariates and the corresponding baseline QoL score were retained.

Differences in fatty acids comparing each of Phenotypes 2 and 3 with the general-population-mapped reference pool were evaluated using Wilcoxon tests in CLR-transformed space. To describe systems-level fatty acid co-variation, phenotype-specific pairwise Pearson correlation networks were constructed from CLR-transformed fatty acid abundances. Edges were displayed for correlations with |*r*| > 0.3. All networks used a fixed spring layout derived from the general-population-mapped reference pool to facilitate visual comparison, and node colours represented mean CLR differences between the target phenotype and the general-population-mapped reference pool. Network analyses were exploratory and descriptive; no formal statistical tests were performed to compare network topology or edge density across phenotypes.

Statistical significance was set at 2-sided *p* < 0.05. Analyses were performed using R version 4.3.3 (R Foundation for Statistical Computing, Vienna, Austria).

## 3. Results

### 3.1. Baseline Participant Profiles and Cross-Sectional Associations with QoL

The final baseline analytical sample included 3009 cancer survivors (mean [SD] age, 64.9 [7.0] years; 2397 women [79.7%]; participant selection and cohort formation, Figure S1; baseline characteristics, Table 1). Baseline EORTC QLQ-C30 summary scores differed across multiple sociodemographic, lifestyle, and clinical characteristics. Examples of factors associated with higher summary scores included younger age, male sex, more frequent physical activity, fewer comorbidities, absence of relapse or metastasis, and more favourable disease trajectories.

After adjustment for age and sex, cancer survivors showed distinct whole-blood fatty acid profiles compared with the available general-population reference sample (Table S2). The survivor cohort had higher total saturated fatty acids (SFAs) and monounsaturated fatty acids (MUFAs), and lower total omega-3 and omega-6 polyunsaturated fatty acids (PUFAs; all *p* < 0.001).

In the core adjusted model, the summary score was positively associated with total SFAs, but inversely associated with total omega-3 and omega-6 PUFAs (Table S3). Baseline EORTC QLQ-C30 scores were similar between the complete-case analytic sample (*n* = 2679) and participants excluded because of missing covariates (*n* = 330; Table S4).

### 3.2. Identification of Whole-Blood Fatty Acid Phenotypes

**Figure 1:**
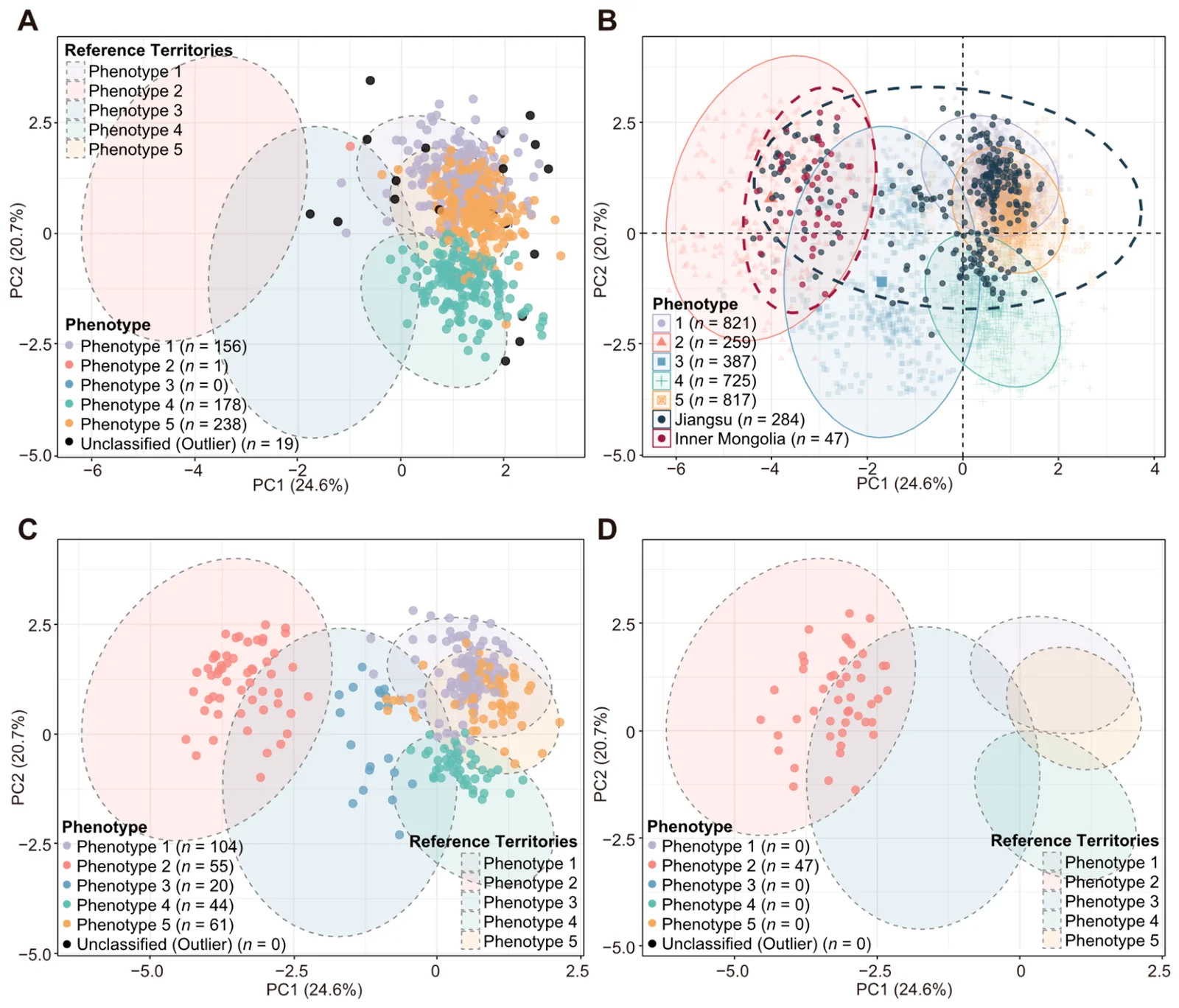
Projection and Assignment of Reference and External Samples Onto the Baseline Principal Component Space. (**A**) Assignment of the available general-population reference sample (*n* = 592) using the Phenotype Calculator. (**B**) Projection of independent external samples from Jiangsu (*n* = 284) and Inner Mongolia (*n* = 47). (**C**) Assignment of the Jiangsu sample using the Phenotype Calculator. (**D**) Assignment of the Inner Mongolia sample using the Phenotype Calculator. Note: In (**B**), dashed and shaded ellipses represent 95% confidence ellipses for the projected samples and the baseline phenotype clusters, respectively. In (**A**,**C**,**D**), dashed ellipses delineate the fixed reference territories of the baseline phenotypes.

Unsupervised clustering identified five distinct whole-blood fatty acid phenotypes (Figure S2; Table S5): Phenotype 1 (moderate SFA, *n* = 821); Phenotype 2 (high SFA/low PUFA, *n* = 259); Phenotype 3 (intermediate SFA-PUFA, *n* = 387); Phenotype 4 (moderate PUFA, *n* = 725); and Phenotype 5 (high PUFA, *n* = 817). Bootstrap analysis indicated high stability of the five-phenotype solution, with mean Jaccard similarities ranging from 0.965 to 0.999 across phenotypes (Table S6). Projection of external samples into the established phenotype space supported the feasibility of phenotype assignment in external cohorts (Figure 1A,B).

The Phenotype Calculator mapped 572 of 573 classified profiles in the available general-population reference sample (*n* = 592) to Phenotypes 1, 4, and 5, with 19 profiles remaining unclassified. These three phenotypes were therefore pooled as the general-population-mapped reference pool. Conversely, Phenotypes 2 and 3 were rarely observed in the available general-population reference sample and appeared enriched among cancer survivors; for brevity, these were referred to as specific phenotypes hereafter. External survivor samples showed marked regional variation: the Jiangsu sample (*n* = 284) showed broad overlap with the Shanghai survivor cohort, whereas all participants in the Inner Mongolia sample (*n* = 47) were assigned to Phenotype 2 (Figure 1C,D).

Because the EORTC QLQ-C30 includes functional, global health, summary, and symptom domains with different scoring directions, higher functional, global health, and summary scores indicate better QoL or functioning, whereas higher symptom scores indicate greater symptom burden.

In the core adjusted model, the summary score and physical, role, cognitive, and social functioning differed significantly across phenotypes (Table 2). Participants assigned to Phenotype 2 had the highest adjusted role and social functioning scores, alongside a relatively high summary score (87.65 [95% CI, 86.09 to 89.21]). By contrast, participants assigned to Phenotype 5 had the lowest adjusted scores across all significantly divergent functional domains and the lowest summary score (86.22 [95% CI, 85.11 to 87.32]). Across these statistically significant domains, the maximum adjusted between-phenotype differences ranged from 1.81 to 4.26 points, all below the 5-point contextual benchmark used for interpretation (Table S7).

### 3.3. Nutritional Characteristics Across Whole-Blood Fatty Acid Phenotypes

Among participants with valid dietary assessments and plausible energy intakes (*n* = 2812), dietary components and nutritional balance varied significantly across phenotypes (Figure S3A,B; Tables S8 and S9). Phenotype 2 was characterised by the highest beef, mutton, and other red meat intake and the greatest DBI-22 excess score for red meat and related animal foods. Phenotype 3 had the lowest total energy intake and the lowest HBS, suggesting the lowest overall dietary excess. Phenotypes 2 and 3 had the highest adjusted dietary variety counts. Nutrition literacy pass rates were comparable across phenotypes (Figure S3C; Table S10).

### 3.4. Quality-of-Life Changes in the Longitudinal Subcohort

**Figure 2:**
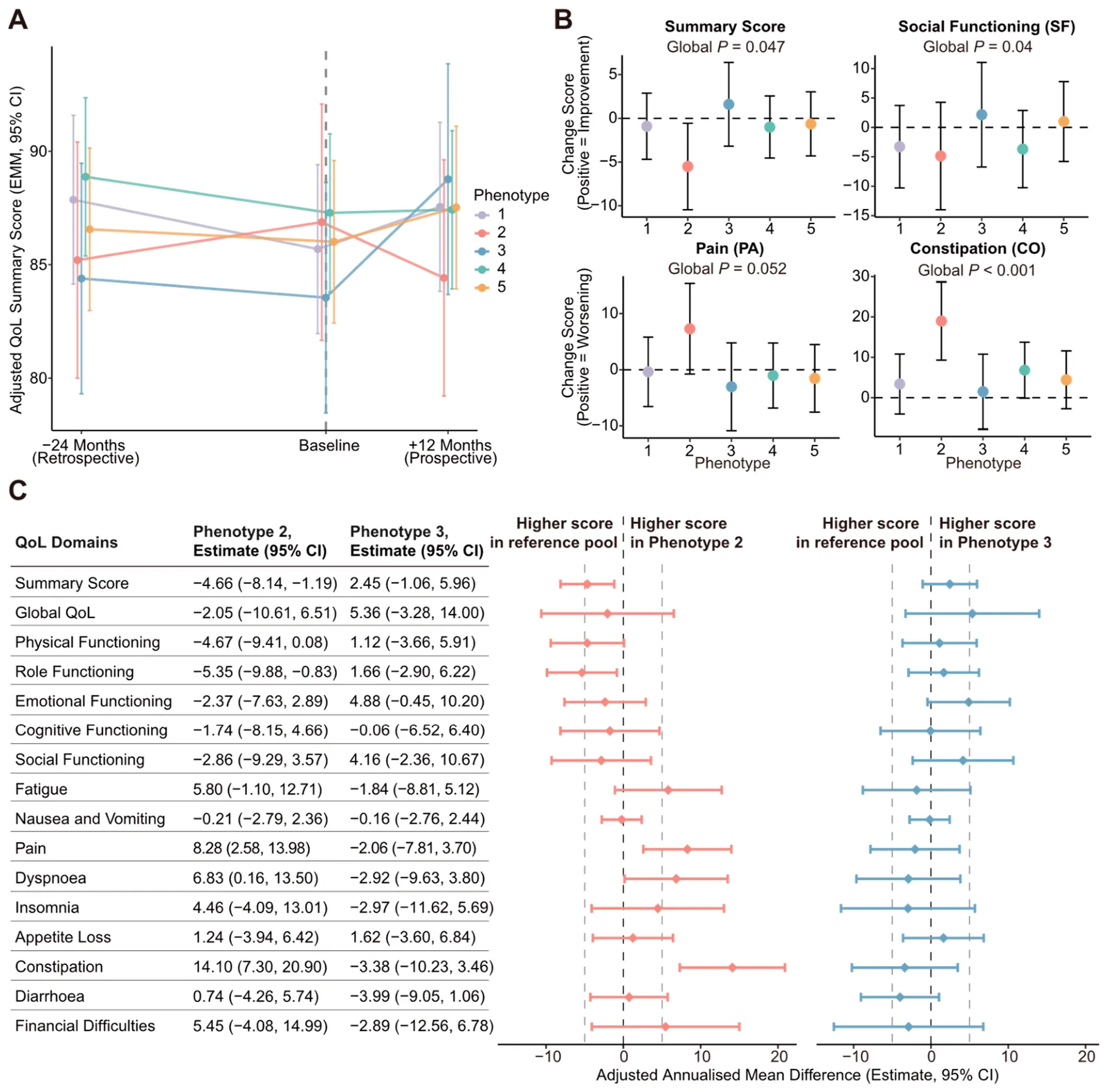
Quality of Life Across Three Assessment Waves and Annualised Changes Across Whole-Blood Fatty Acid Phenotypes. (**A**) Adjusted EORTC QLQ-C30 summary scores at approximately 24 months before baseline, baseline, and 12 months after baseline. (**B**) Adjusted annualised changes in selected QoL domains from baseline to 12-month follow-up. (**C**) Adjusted mean differences in annualised change from baseline to the 12-month follow-up for Phenotypes 2 and 3 compared with the general-population-mapped reference pool. Note: Dashed lines indicate zero and the ±5-point contextual benchmark.

Baseline characteristics differed between the overlapping longitudinal subcohort and the exclusively cross-sectional cohort, and also across whole-blood fatty acid phenotypes (Tables S11 and S12). In the core adjusted model, the EORTC QLQ-C30 summary score remained relatively stable within the general-population-mapped reference pool over the 12-month follow-up, whereas participants assigned to Phenotype 2 showed a decline and those assigned to Phenotype 3 showed an apparent improvement (Figure 2A).

Annualised change analyses showed significant heterogeneity across phenotypes in the summary score, social functioning, and constipation domains (all *p* < 0.05), with Phenotype 2 showing the least favourable direction of change and Phenotype 3 showing the most favourable direction of change (Figure 2B; Table S13).

Compared with the general-population-mapped reference pool, participants assigned to Phenotype 2 had significantly worse annualised changes in five QoL domains, including declines in the summary score (−4.66 [95% CI, −8.14 to −1.19]) and role functioning (−5.35 [95% CI, −9.88 to −0.83]), and worsening pain (8.28 [95% CI, 2.58 to 13.98]), dyspnoea (6.83 [95% CI, 0.16 to 13.50]), and constipation (14.10 [95% CI, 7.30 to 20.90]). For Phenotype 3, apparent favourable changes observed in Model 1 were attenuated and were no longer significant after multivariable adjustment in Model 2 (Figure 2C; Table S14). Based on the point estimates, four of these five statistically significant contrasts exceeded the 5-point contextual benchmark, whereas the summary-score contrast remained below this benchmark (Table S15).

In contrast, annualised QoL changes during the 24 months before the fatty acid assessment did not differ significantly across phenotypes or in contrasts comparing Phenotypes 2 and 3 with the general-population-mapped reference pool (Tables S16 and S17). Sensitivity analyses using multiple imputation produced similar results for both prospective and retrospective analyses (Tables S18–S21), and additional dietary-adjusted and restricted-sample analyses were broadly consistent with the primary prospective findings (Tables S22–S24).

### 3.5. Exploratory Fatty Acid Co-Variation Networks

**Figure 3:**
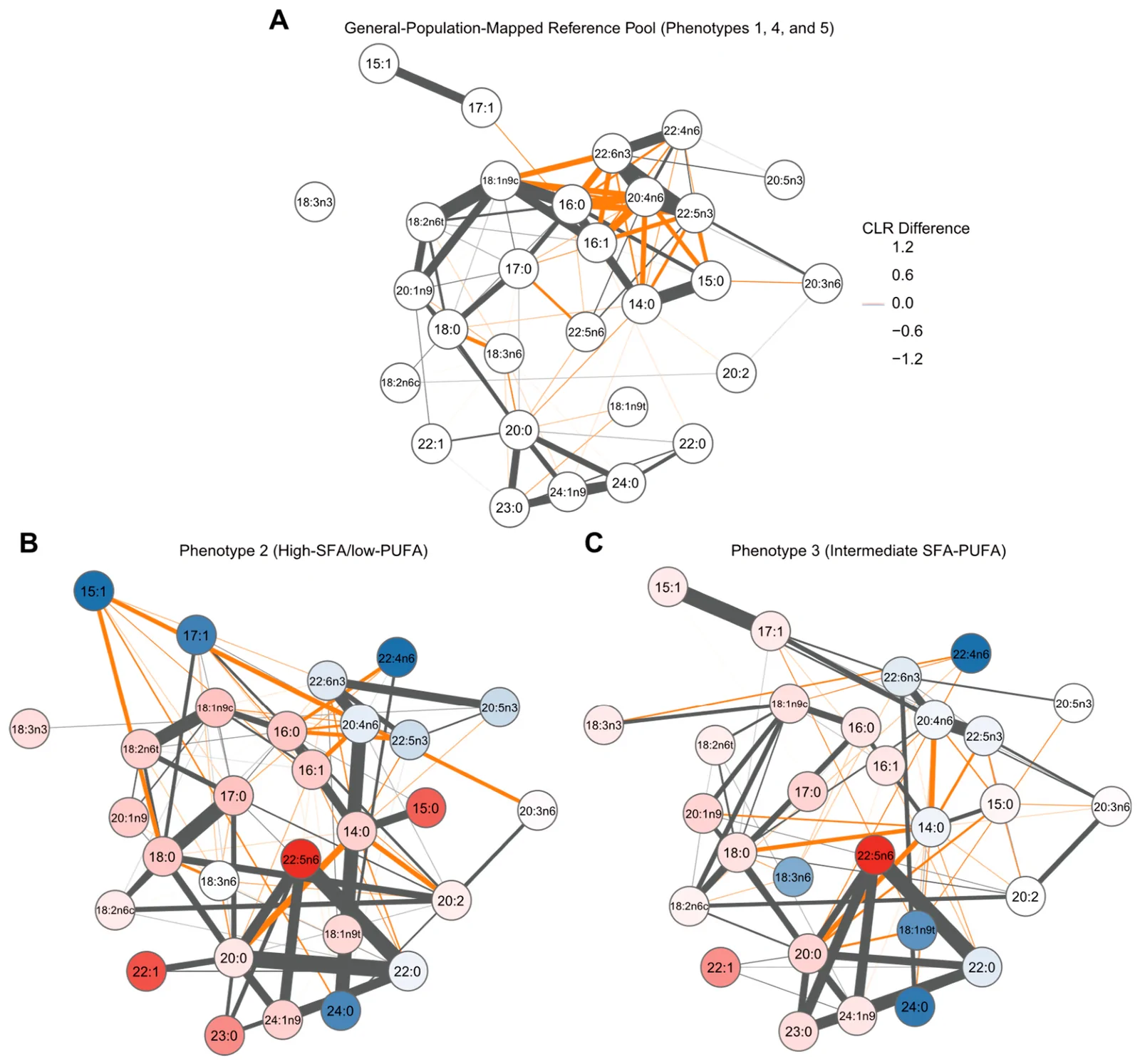
Exploratory Whole-Blood Fatty Acid Co-Variation Networks Across Phenotypes. (**A**) General-population-mapped reference pool (Phenotypes 1, 4, and 5). (**B**) Phenotype 2. (**C**) Phenotype 3. Node colour represents mean CLR difference from the reference pool (red, relative enrichment; blue, relative depletion); edges represent Pearson correlations with |*r*| > 0.3 (dark grey, positive; orange, negative; width proportional to |*r*|).

Compared with the general-population-mapped reference pool (Figure 3A), Phenotypes 2 and 3 showed distinct compositional differences, accompanied by descriptive differences in fatty acid co-variation patterns (Figure S4; Table S25). The reference network was characterised by strong positive correlations among core SFAs, inverse correlations between selected PUFAs and the SFA-rich region, and relative isolation of several MUFAs, including C15:1 and C17:1.

Phenotype 2 showed an SFA-enriched composition (Figure 3B), with relative enrichment of SFAs such as C14:0 and C15:0, as well as C22:5n-6, alongside depletion of MUFAs such as C15:1 and C17:1 and PUFAs such as C22:4n-6. Its co-variation pattern also showed reduced MUFA isolation and attenuation of inverse correlations between selected PUFAs and the SFA-rich region.

Phenotype 3 showed a co-variation pattern descriptively more similar to the general-population-mapped reference pool (Figure 3C), with more localised differences, including C22:5n-6 and C22:1 enrichment and depletion of C22:4n-6, C24:0, and C18:3n-6.

Because phenotype groups differed substantially in sample size and no formal between-network comparison tests were performed, differences in correlation structure were interpreted descriptively.

## 4. Discussion

Among 3009 community-dwelling cancer survivors, this study identified five distinct whole-blood fatty acid phenotypes; 423 participants also contributed data to the overlapping longitudinal subcohort. Two phenotypes were rarely observed in the available general-population reference sample and appeared enriched among cancer survivors, suggesting that long-term survivorship may be accompanied by distinct nutritional-metabolic fatty acid states. In the overlapping longitudinal subcohort, Phenotype 2, a high-SFA/low-PUFA phenotype accompanied by higher red meat intake and greater DBI-22 excess scores for red meat and related animal foods, was associated with worsening across multiple QoL domains over the subsequent 12 months despite relatively favourable baseline QoL. This discordance between cross-sectional QoL status and subsequent change highlights whole-blood fatty acid phenotyping as a potentially informative framework for characterising nutritional-metabolic heterogeneity and aspects of subsequent QoL vulnerability that may not be fully reflected in single-time-point dietary or QoL assessments.

With more than 80% of participants surviving beyond 5 years, this study provides a phenotype-level description of whole-blood fatty acid patterns among predominantly long-term cancer survivors. The available general-population reference sample mapped mainly to Phenotypes 1, 4, and 5, whereas Phenotypes 2 and 3 appeared enriched among survivors, suggesting that cancer survivors may exhibit distinct nutritional-metabolic fatty acid states after hospital-based treatment [31,32]. External survivor samples supported the feasibility of projecting new cohorts into the established phenotype space. The broad overlap between the Jiangsu and Shanghai samples and the assignment of all participants in the small Inner Mongolia sample to the high-SFA/low-PUFA Phenotype 2 further underscore regional heterogeneity, which may partly reflect regional differences in diet and lifestyle [33,34].

Self-reported dietary characteristics corresponded to whole-blood fatty acid phenotypes, consistent with diet as an important exogenous influence on whole-blood fatty acid profiles [10,11,35]. However, these phenotypes should not be interpreted simply as dietary surrogates. Rather, they characterise nutritional-metabolic fatty acid patterns among cancer survivors that are objectively measured in whole-blood DBS. Self-reported dietary assessment captures reported intake over a specified period and is susceptible to recall error, reporting bias, and post-diagnosis dietary changes [36]. By contrast, fasting whole-blood fatty acid profiling provides an objective circulating biomarker that captures fatty acids across cellular and plasma lipid pools. Whole-blood fatty acid composition reflects both dietary exposure and endogenous metabolic influences [10,11,37]. Thus, whole-blood fatty acid phenotyping may capture aspects of nutritional-metabolic status that are not fully reflected in single-time-point self-reported dietary assessment alone.

Phenotype 2 was accompanied by higher beef, mutton, and other red meat intake and greater DBI-22 excess scores for red meat and related animal foods, whereas Phenotype 3 had lower total energy intake and lower overall dietary excess. The comparable nutrition literacy pass rates and omega-3 supplement use across phenotypes suggest that phenotype differences were not simply explained by measured nutrition knowledge or isolated supplement behaviour. The lack of association with omega-3 supplement use is consistent with previous evidence showing heterogeneous or limited effects of single-nutrient supplementation, including omega-3 PUFA supplementation, on cancer-related functional and QoL outcomes [38]. Instead, together with observed differences in sociodemographic, behavioural, and clinical characteristics across phenotypes, these findings suggest that whole-blood fatty acid phenotypes are embedded within a broader survivorship context in which nutrition knowledge may not translate directly into sustained dietary behaviour [39]. The higher red-meat intake observed in Phenotype 2 should be interpreted as a descriptive phenotype characteristic rather than evidence that red-meat intake causally contributes to or mediates subsequent QoL change.

Although previous studies have generally linked higher SFA exposure with less favourable outcomes [40] and omega-3 PUFA exposure with better inflammatory or functional profiles [41,42], baseline cross-sectional analyses in this study showed a counterintuitive pattern: participants assigned to the high-SFA/low-PUFA Phenotype 2 had relatively favourable scores in selected functional domains, whereas those assigned to the high-PUFA Phenotype 5 had the lowest scores across the domains that differed significantly by phenotype. Importantly, although several baseline QoL domains differed statistically across phenotypes, the magnitudes of these cross-sectional differences were all below the 5-point contextual benchmark, indicating relatively small between-group differences at baseline. This apparent paradox may reflect well-recognised epidemiological artefacts in cross-sectional analyses, including indication confounding, reverse causality, and the healthy survivor effect, rather than direct biological effects [43,44,45].

Participants with poorer physical health or greater psychological distress may be more likely to modify their diets, adopt recommended dietary practices, or use nutritional products, creating potential indication confounding [39,46,47]. Conversely, participants with better baseline QoL may have greater capacity to maintain habitual dietary patterns, including higher red-meat intake, which could contribute to a more SFA-enriched whole-blood profile. Together, these considerations provide a plausible explanation for the counterintuitive baseline pattern and underscore the need to interpret cross-sectional fatty acid–QoL associations cautiously.

To complement these cross-sectional findings, analysis of the overlapping longitudinal subcohort—adjusted for baseline QoL [48] and other confounders—showed divergent patterns of subsequent QoL change. The general-population-mapped reference pool showed relatively stable QoL over follow-up, whereas Phenotype 2 was associated with worsening across multiple QoL domains despite relatively favourable baseline QoL. These changes included declines in the summary score and role functioning, as well as worsening pain, dyspnoea, and constipation. In contrast to the relatively small cross-sectional differences, four of the five statistically significant longitudinal contrasts exceeded the 5-point contextual benchmark, indicating larger between-group differences in subsequent QoL change. Notably, additional adjustment for total energy and red-meat intake did not materially attenuate these associations, suggesting that the prospective phenotype-QoL associations were not fully accounted for by the measured dietary characteristics. Although Phenotype 3 showed apparent QoL improvements in the minimally adjusted model, these associations were attenuated and were no longer significant in the core adjusted multivariable model. This attenuation suggests that the apparent improvements in Phenotype 3 may have been partly explained by sociodemographic or clinical covariates [49], consistent with evidence that these factors contribute substantially to QoL trajectories among cancer survivors [50,51].

Annualised QoL changes during the 24 months preceding fatty acid assessment did not differ significantly across phenotypes. Notably, the postbaseline worsening observed in Phenotype 2 was not preceded by a comparable detectable phenotype-specific decline during the available prebaseline period, providing temporal context for the subsequent QoL changes. Because whole-blood fatty acids were measured only at baseline, however, this analysis cannot determine when the phenotypes emerged or establish that the prebaseline and postbaseline periods differed statistically.

In exploratory network analyses, Phenotype 2 showed an SFA-enriched fatty acid profile with depletion of selected MUFAs and PUFAs, accompanied by descriptive differences in fatty acid co-variation, including weaker inverse PUFA–SFA correlations. This pattern may indicate an SFA-enriched metabolic state, but the network differences were not formally tested and this interpretation should therefore be considered hypothesis-generating.

Experimental evidence has linked SFA-enriched or high-fat metabolic states to inflammatory signalling, oxidative stress, mitochondrial dysfunction, and altered immune regulation [52,53]. TLR4-related neuroimmune signalling has also been implicated in persistent pain and nociceptive sensitisation [54]. SFA-rich or high-fat dietary patterns have additionally been associated with gut microbial alterations and impaired intestinal motility [55,56]. These pathways may provide biologically plausible hypotheses for the worsening pain and constipation observed in Phenotype 2; however, they were not directly evaluated in the present study and should therefore be regarded as speculative and hypothesis-generating.

The open-source Whole-Blood Fatty Acid Phenotype Calculator was developed to support reproducible phenotype assignment in future studies. By projecting new whole-blood fatty acid profiles into the fixed baseline PCA space and applying predefined distance boundaries, the tool enables standardised assignment without re-estimating the clustering structure. Together, the external projection results and the observed association between Phenotype 2 and subsequent QoL vulnerability support further evaluation of this phenotype framework in longitudinal survivorship research. In this context, the open-source calculator provides a means for reproducible phenotype assignment and external validation, while its clinical monitoring and predictive utility remain to be established.

## 5. Strengths and Limitations

Strengths of this study include the large community-based survivor cohort, whole-blood fatty acid profiling as an objective circulating biomarker reflecting dietary and endogenous metabolic influences, phenotype derivation based on unsupervised clustering of fatty acid patterns, identification of a cross-sectional–longitudinal discordance supported by longitudinal QoL data, and development of an open-source phenotype assignment tool to support reproducible phenotype assignment in future cohorts.

This study has several limitations. First, the cohort comprised predominantly long-term, community-dwelling cancer survivors from Shanghai with relatively favourable baseline QoL; 79.7% were women and 37.0% were breast cancer survivors. This sex and cancer-type distribution, together with regional heterogeneity in diet, lifestyle, and survivor characteristics, as illustrated by the assignment of all participants in the small Inner Mongolia external sample to Phenotype 2, may limit generalisability to men, underrepresented cancer types, newly diagnosed patients, patients receiving active treatment, and survivors with advanced disease. Sensitivity analyses excluding breast cancer survivors and restricting the analyses to women and to survivors 5–10 years after diagnosis yielded directionally consistent estimates, providing additional support for the findings across these alternative cohort compositions. Second, the observational design precludes causal inference, and residual unmeasured confounding cannot be excluded despite multivariable adjustment. Although cancer type, survivorship duration, and disease trajectory were accounted for, detailed tumour stage and treatment-specific information were not available in the present dataset, which may limit stage- and treatment-specific interpretation. Third, baseline characteristics differed between the overlapping longitudinal subcohort and the exclusively cross-sectional cohort, introducing potential selection bias. The longitudinal subcohort nevertheless retained overlap across major measured covariate strata, allowing multivariable adjustment for observed differences. Residual selection bias from unmeasured factors cannot be excluded, and the smaller longitudinal sample size may have limited statistical power, particularly for Phenotypes 2 and 3. Nevertheless, the observed differences for Phenotype 2 were sizeable and were directionally consistent in sensitivity analyses using multiple imputation within the longitudinal subcohort. Fourth, dietary intake was assessed using self-reported food frequency questionnaires, which are susceptible to recall and reporting bias. Dietary variables were used to characterise phenotype-specific nutritional profiles rather than to establish causal dietary determinants of QoL deterioration. Repeated dietary records during follow-up were not available, limiting our ability to determine whether post-baseline dietary changes contributed to subsequent QoL changes or to compare baseline whole-blood fatty acid phenotyping directly with repeated longitudinal dietary assessment. Fifth, whole-blood fatty acid profiles were measured only at baseline; therefore, single-point sampling cannot assess the temporal stability of phenotype membership, characterise time-varying fatty acid patterns, or determine when the phenotypes emerged. Moreover, the prebaseline and postbaseline QoL intervals occurred in different calendar and social-health contexts; therefore, the prebaseline comparison should be interpreted as providing temporal context rather than as an equivalent negative-control period. Finally, although external samples supported the feasibility of phenotype projection, they did not include comparable longitudinal QoL outcomes, and the observed longitudinal associations therefore require validation in independent cohorts. Moreover, the present analyses were designed to examine associations rather than predictive performance; incremental predictive value beyond conventional clinical, dietary, and QoL information, discrimination, calibration, and clinical utility were not evaluated. Network analyses were exploratory and based on correlation structure rather than direct mechanistic measurements. Future studies incorporating external longitudinal validation, repeated biospecimen collection, mechanistic studies, intervention research, and evaluation of feasibility, accessibility, and cost-effectiveness could refine this phenotype approach and clarify whether it could have utility for nutritional-metabolic monitoring.

## 6. Conclusions

Whole-blood fatty acid profiling identified distinct nutritional-metabolic phenotypes among community-dwelling cancer survivors. The high-SFA/low-PUFA phenotype, accompanied by higher red meat intake and greater DBI-22 excess scores for red meat, was associated with worsening across multiple QoL domains over the subsequent 12 months despite relatively favourable baseline QoL. This discordance between cross-sectional QoL status and subsequent change highlights whole-blood fatty acid phenotyping as a potentially informative framework for characterising nutritional-metabolic heterogeneity and aspects of subsequent QoL vulnerability that may not be fully reflected in single-time-point dietary or QoL assessments. The open-source phenotype assignment tool may facilitate reproducible phenotype assignment and external validation in future cohorts. Further longitudinal validation and intervention studies are needed to clarify the potential utility of whole-blood fatty acid phenotyping for nutritional-metabolic monitoring and for informing nutritional strategies in survivorship care.

## Figures and Tables

**Table 1:** Baseline Sociodemographic, Lifestyle, and Clinical Characteristics of Cancer Survivors and Their Associations With EORTC QLQ-C30 Summary Scores.

Characteristics	No. (%)	EORTC QLQ-C30 Summary Score Mean (SD)	*p* Value ^a^
Age group, y (*n* = 3009)			<0.001
<60	746 (24.8)	88.8 (10.8)	
60–64	634 (21.1)	88.2 (10.9)	
65–69	911 (30.3)	86.8 (11.7)	
≥70	718 (23.9)	85.1 (12.1)	
Sex (*n* = 3009)			<0.001
Male	612 (20.3)	89.0 (11.0)	
Female	2397 (79.7)	86.7 (11.6)	
Monthly personal income, CNY (*n* = 2984)			<0.001
≤1000	256 (8.6)	86.9 (12.0)	
1001–3000	967 (32.4)	88.4 (10.7)	
3001–5000	1275 (42.7)	86.2 (11.9)	
>5000	486 (16.3)	87.4 (11.5)	
Education (*n* = 2818)			0.12
Primary school or less	248 (8.8)	87.6 (11.7)	
Middle school	1368 (48.5)	87.7 (11.2)	
High school	901 (32.0)	86.6 (11.7)	
College or higher	301 (10.7)	86.9 (10.8)	
Physical activity (*n* = 2915)			<0.001
5–7 times per week	880 (30.2)	89.6 (9.6)	
3–4 times per week	882 (30.3)	87.1 (11.1)	
1–2 times per week	715 (24.5)	86.3 (12.0)	
<1 time per week	438 (15.0)	83.9 (13.7)	
Smoking status (*n* = 2922)			0.052
Never smoker	2641 (90.4)	87.0 (11.5)	
Former smoker	187 (6.4)	87.9 (11.8)	
Current smoker	94 (3.2)	89.8 (10.1)	
Drinking status (*n* = 2895)			<0.001
Never drinker	2475 (85.5)	87.0 (11.5)	
Former drinker	145 (5.0)	86.2 (12.4)	
Current drinker	275 (9.5)	89.6 (10.3)	
Living arrangement (*n* = 2937)			0.03
With spouse	1346 (45.8)	87.0 (11.5)	
With family	225 (7.7)	87.6 (11.0)	
With spouse and family	1117 (38.0)	87.7 (11.2)	
Live alone	249 (8.5)	85.4 (12.9)	
Body mass index (*n* = 2980)			0.10
Underweight	104 (3.5)	84.6 (12.9)	
Normal	1561 (52.4)	87.2 (11.3)	
Overweight	1014 (34.0)	87.5 (11.7)	
Obese	301 (10.1)	87.0 (11.2)	
Number of comorbidities (*n* = 3004)			<0.001
0	883 (29.4)	90.7 (9.3)	
1	952 (31.7)	88.2 (10.7)	
2	586 (19.5)	86.1 (11.6)	
≥3	583 (19.4)	81.2 (13.0)	
Duration of cancer survivorship, y (*n* = 2977)			0.16
<5	350 (11.8)	88.1 (11.1)	
5–10	1115 (37.5)	87.3 (11.7)	
≥10	1512 (50.8)	86.8 (11.5)	
Disease trajectory (*n* = 3007)			<0.001
Cured	1069 (35.6)	89.1 (10.1)	
Well-controlled	608 (20.2)	85.6 (11.8)	
Stable	987 (32.8)	87.8 (11.1)	
Worsened	27 (0.9)	80.6 (14.3)	
Unknown	316 (10.5)	82.4 (14.0)	
Relapse (*n* = 3008)			<0.001
Yes	238 (7.9)	83.8 (13.6)	
No	2770 (92.1)	87.5 (11.2)	
Metastasis (*n* = 3005)			<0.001
Yes	144 (4.8)	83.0 (12.7)	
No	2861 (95.2)	87.4 (11.4)	
Severity of COVID-19 (*n* = 2976)			<0.001
Uninfected	502 (16.9)	88.4 (11.0)	
Asymptomatic	82 (2.8)	91.8 (9.7)	
Mild	1750 (58.8)	88.0 (10.8)	
Moderate	598 (20.1)	83.6 (12.6)	
Severe or critical	44 (1.5)	78.6 (16.1)	
Interval since COVID-19 (*n* = 2396) ^b^			0.15
≤1 month	65 (2.7)	88.5 (9.3)	
>1 month	2331 (97.3)	86.8 (11.7)	
Cancer type (*n* = 3009)			<0.001
Hematologic	73 (2.4)	88.3 (10.6)	
Breast	1113 (37.0)	87.0 (11.6)	
Uterine and cervical	137 (4.6)	89.6 (9.0)	
Colorectal	301 (10.0)	87.7 (11.9)	
Genitourinary	120 (4.0)	88.5 (12.5)	
Head and neck	54 (1.8)	89.1 (8.5)	
Hepatopancreatobiliary	66 (2.2)	89.6 (10.4)	
Lung	361 (12.0)	86.5 (11.4)	
Ovarian	80 (2.7)	86.6 (10.5)	
Thyroid	186 (6.2)	88.6 (11.7)	
Upper gastrointestinal	218 (7.2)	88.0 (10.8)	
Multiple primary cancers	262 (8.7)	83.1 (12.2)	
Others	38 (1.3)	88.9 (11.7)	
Omega-3 supplement use (*n* = 2925)			0.84
Regular user	373 (12.8)	87.2 (10.9)	
Occasional user	466 (15.9)	86.9 (11.5)	
Non-user	2086 (71.3)	87.2 (11.5)	

**Note:** Data are presented as No. (%) for categorical participant characteristics. The EORTC QLQ-C30 summary scores are presented as mean (SD). Differences in sample sizes across characteristics are attributed to missing data. The maximum proportion of missing data was observed for education (6.3%), with missing frequencies for all other covariates remaining at or below 5%. ^a^ *p* values were calculated using independent-samples *t* tests or 1-way analysis of variance. ^b^ The denominator for “interval since COVID-19 infection” was inherently restricted to the subpopulation with a documented history of infection.

**Table 2:** Multivariable-Adjusted Baseline Quality of Life Scores Across Whole-Blood Fatty Acid Phenotypes.

Variables	Phenotype 1 (Moderate SFA, *n* = 821)	Phenotype 2 (High-SFA/Low-PUFA, *n* = 259)	Phenotype 3 (Intermediate SFA-PUFA, *n* = 387)	Phenotype 4 (Moderate PUFA, *n* = 725)	Phenotype 5 (High PUFA, *n* = 817)	Model 1 *p* Value (*N* = 3009)	Model 2 *p* Value (*N* = 2698)	Model 3 *p* Value (*N* = 2679)
EORTC QLQ-C30 Summary Score	88.03 (86.93–89.14)	87.65 (86.09–89.21)	87.41 (86.03–88.79)	87.30 (86.20–88.40)	86.22 (85.11–87.32)	0.17	0.02	0.02
Global Health Status (QL)	68.49 (66.17–70.81)	70.11 (66.84–73.39)	68.83 (65.92–71.73)	70.24 (67.93–72.54)	67.19 (64.87–69.50)	0.09	0.12	0.13
Physical functioning (PF)	86.39 (85.02–87.75)	85.16 (83.24–87.09)	86.77 (85.07–88.48)	85.73 (84.38–87.09)	84.60 (83.24–85.96)	0.19	0.04	0.04
Role functioning (RF)	94.87 (93.43–96.31)	95.30 (93.26–97.34)	94.06 (92.26–95.86)	94.15 (92.72–95.57)	92.79 (91.35–94.23)	0.08	0.03	0.03
Emotional functioning (EF)	87.53 (85.95–89.11)	87.48 (85.24–89.72)	87.83 (85.84–89.81)	87.55 (85.97–89.12)	86.83 (85.25–88.41)	0.95	0.84	0.78
Cognitive functioning (CF)	83.96 (82.19–85.74)	83.70 (81.19–86.20)	82.44 (80.23–84.66)	82.46 (80.70–84.22)	81.23 (79.46–83.00)	0.18	0.04	0.03
Social functioning (SF)	86.87 (84.89–88.84)	87.94 (85.15–90.72)	85.01 (82.54–87.48)	86.11 (84.15–88.07)	83.68 (81.71–85.65)	0.02	0.005	0.004
Fatigue (FA)	22.73 (20.76–24.70)	22.31 (19.52–25.10)	23.57 (21.11–26.04)	23.85 (21.89–25.81)	24.81 (22.84–26.78)	0.26	0.23	0.18
Nausea and vomiting (NV)	2.49 (1.53–3.46)	2.35 (0.99–3.72)	2.57 (1.36–3.78)	2.66 (1.70–3.62)	3.39 (2.43–4.35)	0.53	0.33	0.28
Pain (PA)	11.37 (9.54–13.20)	11.71 (9.12–14.30)	11.75 (9.46–14.04)	12.60 (10.78–14.42)	13.95 (12.12–15.77)	0.36	0.06	0.04
Dyspnoea (DY)	14.29 (12.33–16.25)	13.44 (10.67–16.21)	14.32 (11.86–16.78)	15.26 (13.31–17.21)	16.59 (14.63–18.55)	0.29	0.08	0.06
Insomnia (SL)	18.51 (15.89–21.13)	21.07 (17.37–24.76)	19.07 (15.80–22.35)	19.92 (17.32–22.52)	21.00 (18.39–23.61)	0.30	0.36	0.35
Appetite loss (AP)	8.22 (6.52–9.92)	9.08 (6.67–11.48)	8.04 (5.91–10.17)	9.44 (7.75–11.13)	9.34 (7.64–11.04)	0.55	0.49	0.41
Constipation (CO)	8.66 (6.57–10.74)	9.58 (6.63–12.54)	11.34 (8.72–13.95)	9.53 (7.46–11.61)	9.98 (7.90–12.07)	0.46	0.36	0.35
Diarrhoea (DI)	8.79 (7.09–10.49)	9.97 (7.57–12.37)	8.18 (6.05–10.32)	7.40 (5.71–9.09)	9.17 (7.47–10.87)	0.46	0.18	0.21
Financial difficulties (FI)	17.68 (15.18–20.17)	18.29 (14.77–21.81)	19.49 (16.34–22.63)	18.32 (15.85–20.79)	20.42 (17.93–22.90)	0.43	0.24	0.28

Note: Values are adjusted estimated marginal means (95% CIs) from Model 2. Higher functional, global health, and summary scores indicate better QoL or functioning, whereas higher symptom scores indicate greater symptom burden. *p* values are omnibus tests across phenotypes for Models 1–3.

## Data Availability

Deidentified individual-participant data and the corresponding data dictionary are not publicly available because of participant privacy protections and the conditions of ethics approval. Access to deidentified data may be considered by the corresponding authors upon reasonable request after publication, subject to approval by the study investigators and relevant institutional ethics committees, and after completion of an appropriate data access agreement. The open-source R-based Whole-Blood Fatty Acid Phenotype Calculator is available in a public GitHub repository: https://github.com/lvl000/Whole-Blood-Fatty-Acid-Phenotype-Calculator (accessed on 8 July 2026). The repository contains the core R script, the serialized parameter file containing the baseline feature list, PCA rotation matrix, cluster centroids, 99% distance rejection boundaries, and zero-imputation fallback limits, as well as a deidentified example validation dataset and README instructions for system requirements, input formatting, and execution. The detailed operational framework is provided in Supplementary File S2, and the mathematical framework for PCA-based projection and distance-based outlier rejection is provided in Methods S3. Additional statistical code used for the analyses may be shared with qualified researchers upon reasonable request to the corresponding authors.

## Supplementary Materials

The following supporting information can be downloaded at: https://www.mdpi.com/article/10.3390/nu18172838/s1, Supplementary File S1: Supplementary Methods, Tables, and Figures; Methods S1. Whole-Blood Fatty Acid Extraction and Quantification; Methods S2. Dietary Assessment, Preprocessing, and Diet Balance Index (DBI-22) Scoring Algorithms; Methods S3. Construction of the Phenotype Calculator; Table S1. Components, Quantification Methods, and DBI-22 Mapping of the 20-Item SQFFQ; Table S2. Comparison of Whole-Blood Fatty Acid Profiles Between Cancer Survivors and the Available General-Population Reference Sample; Table S3. Quality of Life Scores Across Quartiles of Whole-Blood Fatty Acids and Multivariable-Adjusted Trends; Table S4. Comparison of Baseline Quality of Life Scores Between Included and Excluded Cancer Survivors; Table S5. Baseline Whole-Blood Fatty Acid Profiles and Derived Indices Across Phenotypes; Table S6. Bootstrap Stability of the Five Whole-Blood Fatty Acid Phenotypes; Table S7. Contextual Interpretation of Adjusted Baseline EORTC QLQ-C30 Differences Across Whole-Blood Fatty Acid Phenotypes; Table S8. Covariate-Adjusted Dietary Intake Profiles Across Whole-Blood Fatty Acid Phenotypes; Table S9. Covariate-Adjusted Chinese Dietary Balance Index-22 (DBI-22) Component Scores Across Whole-Blood Fatty Acid Phenotypes; Table S10. Covariate-Adjusted Pass Rates for Nutrition Literacy Domains Across Whole-Blood Fatty Acid Phenotypes; Table S11. Comparison of Baseline Characteristics Between the Overlapping Longitudinal Subcohort and the Exclusively Cross-sectional Cohort; Table S12. Baseline Characteristics of Cancer Survivors Across Whole-Blood Fatty Acid Phenotypes; Table S13. Adjusted Annualised Changes in Quality of Life From Baseline to the 12-Month Follow-Up Across Whole-Blood Fatty Acid Phenotypes; Table S14. Differences in Annualised Quality-of-Life Change From Baseline to the 12-Month Follow-Up for Specific Phenotypes Compared With the General-Population-Mapped Reference Pool; Table S15. Contextual Interpretation of Differences in Annualised Quality-of-Life Change; Table S16. Adjusted Annualised Changes in Quality of Life From the 24-Month Retrospective Assessment to the Baseline Across Whole-Blood Fatty Acid Phenotypes; Table S17. Differences in Annualised Quality-of-Life Change From the 24-Month Retrospective Assessment to Baseline for Specific Phenotypes Compared With the General-Population-Mapped Reference Pool; Table S18. Adjusted Annualised Changes in Quality of Life From Baseline to the 12-Month Follow-Up Across Whole-Blood Fatty Acid Phenotypes Using Multiple Imputation; Table S19. Differences in Annualised Quality-of-Life Change From Baseline to the 12-Month Follow-Up for Specific Phenotypes Compared With the General-Population-Mapped Reference Pool Using Multiple Imputation; Table S20. Adjusted Annualised Changes in Quality of Life From the 24-Month Retrospective Assessment to the Baseline Across Whole-Blood Fatty Acid Phenotypes Using Multiple Imputation; Table S21. Differences in Annualised Quality-of-Life Change From the 24-Month Retrospective Assessment to Baseline for Specific Phenotypes Compared With the General-Population-Mapped Reference Pool Using Multiple Imputation; Table S22. Dietary-Adjusted Sensitivity Analyses of Differences in Annualised Quality-of-Life Change From Baseline to the 12-Month Follow-Up for Phenotypes 2 and 3 Compared With the General-Population-Mapped Reference Pool; Table S23. Restricted-Sample Sensitivity Analyses of Differences in Annualised Quality-of-Life Change From Baseline to the 12-Month Follow-Up for Phenotype 2 Compared With the General-Population-Mapped Reference Pool; Table S24. Restricted-Sample Sensitivity Analyses of Differences in Annualised Quality-of-Life Change From Baseline to the 12-Month Follow-Up for Phenotype 3 Compared With the General-Population-Mapped Reference Pool; Table S25. Differentially Abundant Whole-Blood Fatty Acids Between Specific Phenotypes and the General-Population-Mapped Reference Pool; Figure S1. Participant Selection and Cohort Formation; Figure S2. Unsupervised Clustering of Whole-Blood Fatty Acid Profiles Among Cancer Survivors; Figure S3. Dietary Intake Profiles, Nutritional Balance, and Nutrition Literacy Across Whole-Blood Fatty Acid Phenotypes; Figure S4. Differential Whole-Blood Fatty Acid Signatures for Phenotypes 2 and 3 Compared With the General-Population-Mapped Reference Pool. Supplementary File S2: Toolkit for the Whole-Blood Fatty Acid Phenotype Calculator.

## Abbreviations

AA, arachidonic acid; ANCOVA, analysis of covariance; BMI, body mass index; CI, confidence interval; CLR, centred log-ratio; CNY, Chinese yuan; DBI-22, Diet Balance Index 2022; DBS, dried blood spot; DHA, docosahexaenoic acid; DQD, Diet Quality Distance; EMM, estimated marginal mean; EORTC QLQ-C30, European Organisation for Research and Treatment of Cancer Quality of Life Questionnaire Core 30; EPA, eicosapentaenoic acid; FAME, fatty acid methyl ester; HBS, High Bound Score; IQR, interquartile range; LBS, Low Bound Score; LMM, linear mixed model; MICE, multiple imputation by chained equations; MUFA, monounsaturated fatty acid; PCA, principal component analysis; PC, principal component; PUFA, polyunsaturated fatty acid; QoL, quality of life; SD, standard deviation; SFA, saturated fatty acid; SQFFQ, semi-quantitative food frequency questionnaire.
