# Supplementary material for: Whole-Blood Fatty Acid Phenotypes and Subsequent Quality-of-Life Changes Among Cancer Survivors
Source: Nutrients. 2026 Aug 28;18(17):2838. doi: 10.3390/nu18172838 (PMC13567407; doi:10.3390/nu18172838)
Supplement: Supplementary file 1 [file nutrients-18-02838-s001.zip › nutrients-4459442-supplementary/Supplementary_File_S1_Supplementary_Methods_Tables_Figures.pdf]

Supplementary File S1. Supplementary Methods, Tables, and Figures

Methods S1. Whole-Blood Fatty Acid Extraction and Quantification
Methods S2. Dietary Assessment, Preprocessing, and Diet Balance Index (DBI-22) Scoring Algorithms
Methods S3. Construction of the Phenotype Calculator
Table S1. Components, Quantification Methods, and DBI-22 Mapping of the 20-Item SQFFQ
Table S2. Comparison of Whole-Blood Fatty Acid Profiles Between Cancer Survivors and the Available General-Population Reference Sample
Table S3. Quality of Life Scores Across Quartiles of Whole-Blood Fatty Acids and Multivariable-Adjusted Trends
Table S4. Comparison of Baseline Quality of Life Scores Between Included and Excluded Cancer Survivors
Table S5. Baseline Whole-Blood Fatty Acid Profiles and Derived Indices Across Phenotypes
Table S6. Bootstrap Stability of the Five Whole-Blood Fatty Acid Phenotypes
Table S7. Contextual Interpretation of Adjusted Baseline EORTC QLQ-C30 Differences Across Whole-Blood Fatty Acid Phenotypes
Table S8. Covariate-Adjusted Dietary Intake Profiles Across Whole-Blood Fatty Acid Phenotypes
Table S9. Covariate-Adjusted Chinese Dietary Balance Index-22 (DBI-22) Component Scores Across Whole-Blood Fatty Acid Phenotypes
Table S10. Covariate-Adjusted Pass Rates for Nutrition Literacy Domains Across Whole-Blood Fatty Acid Phenotypes
Table S11. Comparison of Baseline Characteristics Between the Overlapping Longitudinal Subcohort and the Exclusively Cross-sectional Cohort
Table S12. Baseline Characteristics of Cancer Survivors Across Whole-Blood Fatty Acid Phenotypes
Table S13. Adjusted Annualised Changes in Quality of Life From Baseline to the 12-Month Follow-Up Across Whole-Blood Fatty Acid Phenotypes
Table S14. Differences in Annualised Quality-of-Life Change From Baseline to the 12-Month Follow-Up for Specific Phenotypes Compared With the General-Population-Mapped Reference Pool
Table S15. Contextual Interpretation of Differences in Annualised Quality-of-Life Change
Table S16. Adjusted Annualised Changes in Quality of Life From the 24-Month Retrospective Assessment to the Baseline Across Whole-Blood Fatty Acid Phenotypes
Table S17. Differences in Annualised Quality-of-Life Change From the 24-Month Retrospective Assessment to Baseline for Specific Phenotypes Compared With the General-Population-Mapped Reference Pool
Table S18. Adjusted Annualised Changes in Quality of Life From Baseline to the 12-Month Follow-Up Across Whole-Blood Fatty Acid Phenotypes Using Multiple Imputation
Table S19. Differences in Annualised Quality-of-Life Change From Baseline to the 12-Month Follow-Up for Specific Phenotypes Compared With the General-Population-Mapped Reference Pool Using Multiple Imputation
Table S20. Adjusted Annualised Changes in Quality of Life From the 24-Month Retrospective Assessment to the Baseline Across Whole-Blood Fatty Acid Phenotypes Using Multiple Imputation
Table S21. Differences in Annualised Quality-of-Life Change From the 24-Month Retrospective Assessment to Baseline for Specific Phenotypes Compared With the General-Population-Mapped Reference Pool Using Multiple Imputation

Table S22. Dietary-Adjusted Sensitivity Analyses of Differences in Annualised Quality-of-Life Change From Baseline to the 12-Month Follow-Up for Phenotypes 2 and 3 Compared With the General-Population-Mapped Reference Pool
Table S23. Restricted-Sample Sensitivity Analyses of Differences in Annualised Quality-of-Life Change From Baseline to the 12-Month Follow-Up for Phenotype 2 Compared With the General-Population-Mapped Reference Pool
Table S24. Restricted-Sample Sensitivity Analyses of Differences in Annualised Quality-of-Life Change From Baseline to the 12-Month Follow-Up for Phenotype 3 Compared With the General-Population-Mapped Reference Pool
Table S25. Differentially Abundant Whole-Blood Fatty Acids Between Specific Phenotypes and the General-Population-Mapped Reference Pool
Figure S1. Participant Selection and Cohort Formation
Figure S2. Unsupervised Clustering of Whole-Blood Fatty Acid Profiles Among Cancer Survivors
Figure S3. Dietary Intake Profiles, Nutritional Balance, and Nutrition Literacy Across Whole-Blood Fatty Acid Phenotypes
Figure S4. Differential Whole-Blood Fatty Acid Signatures for Phenotypes 2 and 3 Compared With the General-Population-Mapped Reference Pool

Methods S1. Whole-Blood Fatty Acid Extraction and Quantification

Fasting capillary whole blood was collected by finger prick in the morning and applied directly to dried blood spot (DBS) cards. The DBS samples were stored at 5 °C and analysed within 1 week of collection.

A standardised punch from each DBS card was combined with a methylating mixture comprising boron trifluoride in methanol (14%; ANPEL Laboratory Technologies (Shanghai) Inc., Shanghai, China), toluene (Sinopharm Chemical Reagent Co., Ltd., Shanghai, China), and methanol (Sinopharm Chemical Reagent Co., Ltd., Shanghai, China) (35:30:35, v/v/v), shaken vigorously, and heated at 100 °C for 45 minutes. After cooling, equal volumes of n-heptane (ANPEL Laboratory Technologies (Shanghai) Inc., Shanghai, China) and distilled water were added. Following brief vortexing and centrifugation to separate the layers, an aliquot of the upper n-heptane layer containing the fatty acid methyl esters (FAMEs) was collected for gas chromatographic analysis.

Gas chromatography was performed using an Agilent 7820A system (Agilent Technologies, Santa Clara, CA, USA) equipped with a DB-23 capillary column (60 m; Agilent Technologies, Santa Clara, CA, USA). A commercial FAME reference mixture (Nu-Chek-Prep, Inc., Waterville, MN, USA) was analysed with each sample batch for quality control. Fatty acids were identified and quantified based on retention times and chromatographic peak areas of the commercial FAME reference mixture using Agilent OpenLab CDS ChemStation Edition (version C.01.10; Agilent Technologies, Santa Clara, CA, USA). No internal standard was used. A total of 37 fatty acids were identified, and results were expressed as relative percentages of the total identified whole-blood fatty acid pool.

Methods S2. Dietary Assessment, Preprocessing, and Diet Balance Index (DBI-22) Scoring Algorithms

Dietary Assessment and Quantification

Habitual dietary intake over the past year was assessed using a customised 20-item semi-quantitative food frequency questionnaire (SQFFQ) (Table S1). To mitigate recall bias and cognitive burden inherent in portion size estimation—particularly considering the varying educational levels and health literacy among cancer survivors—standard visual references ("fist equivalents") were employed for solid foods, while standardised volumetric units were used for liquids. The consumption of cooking oil, added salt, and sugar was estimated using a household inventory allocation method (total household consumption divided by household size and duration).

Data Preprocessing and Cooked-to-Raw Conversion

As referenced in the main text, self-reported cooked food intakes were systematically converted to raw weights. The portion quantification strategies and yield factors derived from the China Food Composition Tables are provided in Table S1. Estimated Energy Requirements (EER) were assigned based on age and sex.

DBI-22 Scoring Algorithms

The overall diet quality was evaluated using the updated Chinese Diet Balance Index (DBI-22). To characterise dietary adherence, the DBI-22 framework evaluates intakes across 14 specific scoring sub-dimensions: cereals, vegetables, fruits, dairy, soybeans, red meat and poultry, fish and shrimp, eggs, cooking oil, alcoholic beverages, added sugar, salt, diet variety, and drinking water. Owing to questionnaire limitations, alcoholic beverage scores were estimated based on self-reported consumption frequency rather than quantitative volumetric assessments. Participants were categorised into 11 distinct energy requirement levels (ranging from 1000 to 3000 kcal) based on their estimated EER, with individualised optimal intake thresholds applied accordingly. Following the DBI-22 framework, three aggregate scores were derived to capture multidimensional dietary imbalances: Low Bound Score (LBS): The absolute sum of all negative component scores, reflecting the severity of inadequate dietary intake; High Bound Score (HBS): The sum of all positive component scores, indicating the degree of excessive dietary intake; Diet Quality Distance (DQD): The sum of LBS and HBS, serving as a comprehensive indicator of overall dietary imbalance.

Methods S3. Construction of the Phenotype Calculator

To facilitate reproducible phenotype assignment across independent cohorts, we developed an open-source computational tool (version 1.0.0; Supplementary File S2). This calculator projects external whole-blood fatty acid profiles into the fixed baseline phenotype space without re-estimating the principal component space. The framework involves 3 operational steps:

Batch-adaptive zero imputation

To accommodate variation in analytical detection limits, exact zero values are imputed using half the minimum nonzero value observed within the external batch. If the external dataset contains a single profile or if a specific fatty acid is entirely undetected in the external batch, the calculator uses baseline half-minimum fallback values embedded in the core RData file.

Fixed-space projection

After centred log-ratio (CLR) transformation, external data are projected into the established 7-dimensional principal component space. This projection uses the fixed centring vector and rotation matrix derived from the baseline cohort, maintaining consistency with the baseline PCA space.

Distance-based assignment and outlier rejection

The calculator computes the multidimensional Euclidean distance from each new profile to the established baseline centroids. A profile is assigned to the nearest phenotype only if this distance falls within the phenotype-specific 99th percentile intracluster distance threshold. Profiles exceeding this threshold are flagged as "Unclassified (Outlier)".

Table S1. Components, Quantification Methods, and DBI-22 Mapping of the 20-Item SQFFQ

Food Categories (Items)	Frequency Assessment	Portion Quantification & Conversion Strategy	Mapping to DBI-22 Sub-dimensions
Rice	Times per week	Visual reference (fist) / Raw weight = Cooked weight / 2.3	Cereal (LBS/HBS)
Wheat-based foods	Times per week	Visual reference (fist) / Raw weight = Cooked weight / 1.4	Cereal (LBS/HBS)
Whole grains	Times per week	Visual reference (fist) / Assumed as raw weight	Cereal (LBS/HBS)
Fried wheat foods	Times per week	Visual reference (fist) / Added to total energy	(Not scored in DBI-22)
Pork and pork products	Times per week	Visual reference (fist) / Raw weight = Cooked weight / 0.65	Red meat and poultry (LBS/HBS)
Beef, mutton, and other red meats	Times per week	Visual reference (fist) / Raw weight = Cooked weight / 0.65	Red meat and poultry (LBS/HBS)
Poultry	Times per week	Visual reference (fist) / Raw weight = Cooked weight / 0.65	Red meat and poultry (LBS/HBS)
Seafood	Times per week	Visual reference (fist) / Raw weight = Cooked weight / 0.7	Fish and shrimp (LBS)
Eggs and egg products	Times per week	Visual reference (fist) / Assumed as raw weight	Egg (LBS/HBS)
Light-colored vegetables	Times per week	Visual reference (fist) / Assumed as raw weight	Vegetable (LBS)
Dark-colored vegetables	Times per week	Visual reference (fist) / Assumed as raw weight	Diet variety
Soybean products	Times per week	Visual reference (fist) / Raw weight = Cooked weight / 2.5	Soybean (LBS)
Pickled and smoked foods	Times per week	Visual reference (fist) / Assumed as raw weight	(Not scored in DBI-22)
Fresh fruits	Times per week	Visual reference (fist) / Assumed as raw weight	Fruit (LBS)
Nuts and seeds	Times per week	Visual reference (fist) / Evaluated in variety score	(Not scored in DBI-22)
Dairy products	Times per week	Standard volumetric unit (250 mL/cup)	Dairy (LBS)
Drinking water	Daily	Standard volumetric unit (500 mL/bottle)	Drinking water (LBS)
Cooking oil	-	Household inventory method (days/5L/household size)	Cooking oil (HBS)
Salt	-	Household inventory method (days/350g/household size)	Salt (HBS)
Added sugar	-	Household inventory method (days/500g/household size)	Added sugar (HBS)
(Supplemental) Alcoholic beverages[a]	Categorical	Evaluated strictly via frequency	Alcoholic beverage (HBS)

Abbreviations: DBI-22, Diet Balance Index 2022; HBS, High Bound Score; LBS, Low Bound Score; SQFFQ, semi-quantitative food frequency questionnaire.

Items marked as "Not scored in DBI-22" (eg, fried wheat foods, pickled and smoked foods, nuts and seeds) were included in the SQFFQ to capture comprehensive dietary habits and calculate total energy intake but do not independently contribute to the standardised DBI-22 dimensional scoring algorithms.

[a] Alcoholic beverages were not part of the core 20-item SQFFQ food list but were assessed as a supplemental categorical item. Corresponding HBS penalties were estimated based solely on self-reported consumption frequency.

Table S2. Comparison of Whole-Blood Fatty Acid Profiles Between Cancer Survivors and the Available General-Population Reference Sample

**Fatty Acids and Derived Indices**	**Cancer survivors (*n* = 3009)**	**General population (*n* = 592)**	***P* value[a]**
Total SFA	42.65 [40.58, 44.87]	40.58 [38.67, 42.63]	<0.001
Myristic acid (14:0)	0.26 [0.16, 0.40]	0.26 [0.17, 0.39]	0.60
Pentadecanoic acid (15:0)	0.15 [0.10, 0.19]	0.15 [0.11, 0.20]	0.72
Palmitic acid (16:0)	22.45 [20.53, 24.52]	20.89 [18.94, 22.49]	<0.001
Margaric acid (17:0)	0.41 [0.35, 0.46]	0.39 [0.34, 0.46]	0.006
Stearic acid (18:0)	14.49 [13.45, 15.66]	14.01 [13.12, 14.87]	<0.001
Arachidic acid (20:0)	0.46 [0.39, 0.52]	0.43 [0.38, 0.51]	<0.001
Heneicosanoic acid (21:0)	0.00 [0.00, 0.09]	0.00 [0.00, 0.07]	0.007
Behenic acid (22:0)	1.78 [1.40, 2.24]	1.73 [1.33, 2.22]	0.14
Tricosanoic acid (23:0)	0.36 [0.30, 0.44]	0.34 [0.28, 0.40]	<0.001
Lignoceric acid (24:0)	2.15 [1.35, 2.62]	2.31 [1.90, 2.70]	<0.001
Total MUFA	23.36 [21.61, 25.36]	22.06 [20.37, 24.07]	<0.001
Myristoleic acid (14:1)	0.00 [0.00, 0.00]	0.00 [0.00, 0.00]	<0.001
Pentadecenoic acid (15:1)	0.83 [0.65, 0.98]	0.85 [0.70, 1.00]	0.003
Palmitoleic acid (16:1)	0.77 [0.59, 1.01]	0.62 [0.49, 0.81]	<0.001
Heptadecenoic acid (17:1)	1.26 [0.97, 1.50]	1.33 [1.12, 1.61]	<0.001
Elaidic acid (18:1n-9 *trans*)	0.11 [0.00, 0.14]	0.11 [0.00, 0.16]	0.02
Oleic acid (18:1n-9 cis)	16.94 [15.35, 19.02]	15.64 [14.28, 17.52]	<0.001
Gondoic acid (20:1n-9)	0.31 [0.26, 0.36]	0.30 [0.25, 0.37]	<0.001
Erucic acid (22:1)	0.49 [0.33, 0.80]	0.44 [0.29, 0.65]	<0.001
Nervonic acid (24:1n-9)	2.41 [1.97, 2.91]	2.37 [1.88, 2.92]	0.03
Total n-3 PUFA	3.93 [3.05, 5.00]	6.49 [5.31, 7.63]	<0.001
α-Linolenic acid (ALA, 18:3n-3)	0.31 [0.24, 0.41]	0.56 [0.41, 0.77]	<0.001
Eicosatrienoic acid (ETE, 20:3n-3)	0.00 [0.00, 0.00]	0.00 [0.00, 0.00]	<0.001
Eicosapentaenoic acid (EPA, 20:5n-3)	0.36 [0.23, 0.66]	1.44 [0.88, 2.09]	<0.001
Docosapentaenoic acid (DPA, 22:5n-3)	1.01 [0.76, 1.30]	0.93 [0.71, 1.20]	<0.001
Docosahexaenoic acid (DHA, 22:6n-3)	2.04 [1.51, 2.73]	3.35 [2.62, 4.04]	<0.001
Total n-6 PUFA	27.22 [25.18, 29.51]	28.14 [26.17, 30.35]	<0.001
Linolelaidic acid (18:2n-6 *trans*)	1.57 [1.41, 1.76]	1.47 [1.32, 1.64]	<0.001
Linoleic acid (LA, 18:2n-6 cis)	17.20 [15.80, 18.69]	17.24 [15.57, 18.80]	0.01
γ-Linolenic acid (GLA, 18:3n-6)	0.11 [0.00, 0.17]	0.14 [0.00, 0.24]	<0.001
Dihomo-γ-linolenic acid (DGLA, 20:3n-6)	1.16 [0.98, 1.39]	1.04 [0.87, 1.22]	<0.001
Arachidonic acid (AA, 20:4n-6)	7.05 [5.66, 8.63]	7.74 [6.44, 9.12]	<0.001
Docosadienoic acid (22:2n-6)	0.00 [0.00, 0.11]	0.00 [0.00, 0.09]	0.08
Adrenic acid (22:4n-6)	0.77 [0.46, 1.00]	1.44 [1.14, 1.73]	<0.001
Osbond acid (22:5n-6)	0.42 [0.29, 0.76]	0.28 [0.19, 0.37]	<0.001
Eicosadienoic acid (20:2)	0.38 [0.33, 0.43]	0.36 [0.31, 0.41]	<0.001

Table S2. Comparison of Whole-Blood Fatty Acid Profiles Between Cancer Survivors and the Available General-Population Reference Sample (Continued)

**Fatty Acids and Derived Indices**	**Cancer survivors (*n* = 3009)**	**General population (*n* = 592)**	***P* value[a]**
EPA + DHA	2.51 [1.87, 3.36]	4.78 [3.77, 6.00]	<0.001
AA/EPA ratio	19.39 [10.35, 29.94]	5.35 [3.51, 8.46]	<0.001
n-6/n-3 ratio	6.94 [5.73, 8.49]	4.39 [3.64, 5.34]	<0.001

Abbreviations: AA, arachidonic acid; ANCOVA, analysis of covariance; EPA, eicosapentaenoic acid; IQR, interquartile range; MUFA, monounsaturated fatty acid; PUFA, polyunsaturated fatty acid; SFA, saturated fatty acid.

Note: Under the header "Fatty Acids and Derived Indices," individual and composite fatty acids, alongside EPA + DHA, are expressed as relative abundance (percentage of the total measured whole-blood fatty acid pool). Data are presented as median (IQR). Eicosadienoic acid (20:2) and *trans* fatty acids were evaluated individually and excluded from the composite calculations of polyunsaturated or monounsaturated fatty acids. Calculated ratios (AA/EPA and n-6/n-3) are unitless.

[a] *P* values were derived from rank-based analysis of covariance adjusted for age and sex.

Table S3. Quality of Life Scores Across Quartiles of Whole-Blood Fatty Acids and Multivariable-Adjusted Trends

**Fatty Acids and Derived Indices**	**Q1 Mean (SD)**	**Q2 Mean (SD)**	**Q3 Mean (SD)**	**Q4 Mean (SD)**	**Model 1 *P* for trend (*N* = 3009)**	**Model 2 *P* for trend (*N* = 2698)**	**Model 3 *P* for trend (*N* = 2679)**
Total SFA	86.2 (12.0)	86.6 (11.9)	87.9 (10.6)	88.0 (11.3)	0.001	0.003	0.002
Myristic acid (14:0)	86.9 (11.5)	87.2 (12.1)	87.2 (11.2)	87.5 (11.1)	0.46	0.14	0.19
Pentadecanoic acid (15:0)	87.2 (11.3)	87.1 (11.8)	87.1 (12.0)	87.3 (10.9)	0.78	0.79	0.81
Palmitic acid (16:0)	86.6 (11.6)	87.0 (11.9)	87.2 (11.6)	87.9 (10.8)	0.04	0.02	0.02
Margaric acid (17:0)	87.1 (11.2)	86.2 (12.7)	87.3 (11.1)	88.1 (10.8)	0.03	0.051	0.049
Stearic acid (18:0)	85.9 (12.2)	87.1 (11.7)	88.0 (11.0)	87.7 (11.0)	0.001	0.06	0.06
Arachidic acid (20:0)	87.2 (11.6)	87.1 (11.6)	86.7 (11.7)	87.7 (11.1)	0.35	0.29	0.29
Heneicosanoic acid (21:0)	86.8 (11.5)	87.2 (12.1)	87.8 (10.9)	86.8 (11.5)	0.67	0.90	0.99
Behenic acid (22:0)	86.9 (11.7)	87.4 (11.7)	87.6 (10.9)	86.8 (11.7)	0.69	0.47	0.55
Tricosanoic acid (23:0)	87.5 (11.6)	86.8 (11.4)	87.0 (11.8)	87.4 (11.1)	0.44	0.93	0.94
Lignoceric acid (24:0)	87.1 (11.3)	87.2 (11.6)	87.2 (11.5)	87.1 (11.6)	0.84	0.70	0.94
Total MUFA	87.1 (11.6)	86.9 (11.8)	87.5 (11.3)	87.2 (11.3)	0.76	0.15	0.11
Myristoleic acid (14:1)	86.9 (11.4)	87.0 (11.2)	87.3 (12.0)	87.5 (11.4)	NA	NA	NA
Pentadecenoic acid (15:1)	87.6 (10.8)	86.9 (12.1)	86.7 (11.8)	87.5 (11.3)	0.41	0.35	0.35
Palmitoleic acid (16:1)	87.2 (11.2)	87.1 (12.1)	87.3 (11.6)	87.2 (11.0)	0.65	0.09	0.12
Heptadecenoic acid (17:1)	87.5 (10.9)	87.1 (12.1)	86.9 (11.3)	87.2 (11.6)	0.41	0.18	0.20
Elaidic acid (18:1n-9 *trans*)	87.0 (11.5)	87.5 (11.0)	87.1 (12.1)	87.1 (11.4)	0.96	0.45	0.43
Oleic acid (18:1n-9 *cis*)	86.7 (11.9)	87.4 (11.5)	87.6 (11.2)	87.0 (11.4)	0.61	0.11	0.10
Gondoic acid (20:1n-9)	86.7 (11.5)	86.5 (12.0)	87.4 (11.5)	88.0 (10.8)	0.04	0.046	0.03
Erucic acid (22:1)	86.5 (11.7)	87.0 (11.6)	87.4 (11.7)	87.8 (10.9)	0.02	0.02	0.02
Nervonic acid (24:1n-9)	86.7 (11.6)	86.7 (12.1)	87.8 (11.2)	87.5 (11.0)	0.19	0.15	0.15

Table S3. Quality of Life Scores Across Quartiles of Whole-Blood Fatty Acids and Multivariable-Adjusted Trends (Continued)

Fatty Acids and Derived Indices	Q1 Mean (SD)	Q2 Mean (SD)	Q3 Mean (SD)	Q4 Mean (SD)	Model 1 *P* for trend (*N* = 3009)	Model 2 *P* for trend (*N* = 2698)	Model 3 *P* for trend (*N* = 2679)
Total n-3 PUFA	87.9 (11.4)	87.9 (10.5)	86.5 (11.6)	86.3 (12.3)	0.004	0.002	0.001
α-Linolenic acid (ALA, 18:3n-3)	87.6 (10.8)	87.0 (11.3)	87.4 (11.7)	86.6 (12.1)	0.33	0.91	0.84
Eicosatrienoic acid (ETE, 20:3n-3)	87.0 (11.4)	87.0 (11.4)	87.2 (12.0)	87.5 (11.1)	NA	NA	NA
Eicosapentaenoic acid (EPA, 20:5n-3)	87.3 (11.8)	87.6 (10.5)	87.7 (11.4)	86.1 (12.2)	0.03	0.14	0.22
Docosapentaenoic acid (DPA, 22:5n-3)	87.4 (11.4)	87.3 (11.6)	86.5 (11.9)	87.4 (11.1)	0.52	0.60	0.48
Docosahexaenoic acid (DHA, 22:6n-3)	87.7 (11.4)	87.8 (10.6)	87.0 (11.3)	86.1 (12.5)	0.008	<0.001	<0.001
Total n-6 PUFA	87.8 (11.2)	87.5 (11.0)	86.6 (12.0)	86.8 (11.7)	0.051	0.02	0.01
Linolelaidic acid (18:2n-6 *trans*)	86.9 (11.4)	87.7 (11.4)	86.5 (11.8)	87.6 (11.2)	0.93	0.89	0.96
Linoleic acid (LA, 18:2n-6 *cis*)	87.2 (11.4)	87.0 (11.7)	87.8 (10.8)	86.6 (11.9)	0.59	0.33	0.31
γ-Linolenic acid (GLA, 18:3n-6)	87.4 (11.5)	87.3 (11.7)	87.2 (11.1)	86.8 (11.7)	0.55	0.86	0.82
Dihomo-γ-linolenic acid (DGLA, 20:3n-6)	87.4 (11.8)	87.6 (10.9)	87.1 (11.2)	86.6 (11.9)	0.19	0.09	0.06
Arachidonic acid (AA, 20:4n-6)	87.5 (11.5)	88.2 (10.5)	86.4 (12.1)	86.6 (11.8)	0.03	0.02	0.02
Docosadienoic acid (22:2n-6)	86.8 (11.5)	87.4 (11.5)	87.2 (11.7)	87.3 (11.3)	0.79	0.86	0.67
Adrenic acid (22:4n-6)	87.3 (11.1)	87.8 (11.1)	86.5 (11.9)	87.0 (11.9)	0.26	0.10	0.07
Osbond acid (22:5n-6)	87.4 (11.4)	87.4 (11.5)	86.6 (12.1)	87.3 (10.9)	0.86	0.88	0.93
Eicosadienoic acid (20:2)	87.5 (11.3)	87.6 (11.0)	87.0 (12.1)	86.6 (11.5)	0.07	0.21	0.10
EPA + DHA	87.8 (11.4)	87.8 (10.7)	87.0 (11.1)	86.0 (12.5)	0.004	0.001	0.001
AA/EPA ratio	86.5 (11.8)	87.9 (11.7)	87.0 (10.9)	87.3 (11.4)	0.55	0.66	0.81
n-6/n-3 ratio	86.5 (12.3)	86.4 (11.8)	88.2 (10.2)	87.6 (11.4)	0.03	0.02	0.02

Abbreviations: MUFA, monounsaturated fatty acid; PUFA, polyunsaturated fatty acid; SD, standard deviation; SFA, saturated fatty acid.

Note: Unadjusted raw quality of life scores within each quartile are presented as mean (SD) (*n* = 3009). Quartiles (Q1–Q4) for individual and composite fatty acids, as well as EPA + DHA, were defined based on their relative abundances (percentage of total fatty acids), whereas quartiles for the AA/EPA and n-6/n-3 ratios were derived from their

unitless absolute values. Model 1 was adjusted for age, sex, and cancer type. Model 2 was additionally adjusted for body mass index, monthly personal income, smoking status, drinking status, living arrangement, physical activity, number of comorbidities, duration of survivorship, and disease trajectory. Model 3 was further adjusted for COVID-19 severity. NA indicates that the *P* value for trend could not be calculated due to values falling below the limit of detection. Eicosadienoic acid (20:2) and trans fatty acids were analysed individually and were excluded from the composite calculations of polyunsaturated or monounsaturated fatty acids.

Table S4. Comparison of Baseline Quality of Life Scores Between Included and Excluded Cancer Survivors

**Variables**	**Included, Mean (SD) (*n* = 2679)**[a]	**Excluded, Mean (SD) (*n* = 330)**	***P* value**[a]
EORTC QLQ-C30 Summary Score	87.2 (11.5)	87.2 (11.3)	0.93
Global Health Status (QL)	85.5 (14.0)	85.4 (14.2)	0.91
Physical functioning (PF)	94.2 (14.1)	93.8 (14.8)	0.67
Role functioning (RF)	88.2 (15.7)	88.5 (15.8)	0.76
Emotional functioning (EF)	82.1 (17.6)	82.1 (19.1)	0.99
Cognitive functioning (CF)	86.5 (19.7)	86.3 (18.5)	0.88
Social functioning (SF)	69.4 (23.4)	68.4 (24.5)	0.46
Fatigue (FA)	23.2 (20.2)	21.8 (19.7)	0.23
Nausea and vomiting (NV)	2.7 (9.4)	2.5 (7.6)	0.74
Pain (PA)	13.2 (18.6)	13.2 (18.2)	0.96
Dyspnoea (DY)	14.9 (19.8)	14.7 (20.8)	0.88
Insomnia (SL)	21.9 (26.1)	20.2 (27.1)	0.27
Appetite loss (AP)	8.1 (16.7)	8.1 (16.1)	0.95
Constipation (CO)	11.4 (20.5)	12.1 (20.0)	0.53
Diarrhoea (DI)	7.7 (16.5)	9.5 (17.6)	0.09
Financial difficulties (FI)	17.5 (24.7)	16.0 (22.1)	0.25

Abbreviations: EORTC QLQ-C30, European Organisation for Research and Treatment of Cancer Quality of Life Questionnaire Core 30; SD, standard deviation.

Note: Data are presented as mean (SD). Included participants (*n* = 2679) were defined as those with complete data for all covariates in the final multivariable-adjusted model (Model 3). Excluded participants (*n* = 330) represent individuals omitted due to missing covariate data.

[a] Comparisons between included and excluded participants were performed using independent-samples *t* tests.

Table S5. Baseline Whole-Blood Fatty Acid Profiles and Derived Indices Across Phenotypes

Fatty Acids and Derived Indices	Phenotype 1 (Moderate SFA, *n* = 821)	Phenotype 2 (High-SFA/low-PUFA, *n* = 259)	Phenotype 3 (Intermediate SFA-PUFA, *n* = 387)	Phenotype 4 (Moderate PUFA, *n* = 725)	Phenotype 5 (High PUFA, *n* = 817)
Total SFA	44.41 [42.64, 46.14]	45.07 [42.57, 46.95]	42.48 [41.02, 44.09]	42.53 [40.42, 44.62]	40.58 [38.93, 42.28]
Myristic acid (14:0)	0.42 [0.32, 0.55]	0.32 [0.20, 0.43]	0.26 [0.16, 0.38]	0.21 [0.13, 0.31]	0.18 [0.12, 0.26]
Pentadecanoic acid (15:0)	0.17 [0.15, 0.20]	0.28 [0.22, 0.34]	0.17 [0.10, 0.21]	0.13 [0.09, 0.17]	0.11 [0.09, 0.13]
Palmitic acid (16:0)	24.32 [23.07, 25.80]	24.83 [22.93, 26.53]	23.46 [22.04, 24.87]	21.48 [19.61, 23.33]	20.43 [18.94, 21.66]
Margaric acid (17:0)	0.42 [0.37, 0.46]	0.45 [0.40, 0.49]	0.46 [0.42, 0.51]	0.41 [0.36, 0.47]	0.35 [0.32, 0.39]
Stearic acid (18:0)	13.95 [12.95, 15.05]	15.98 [14.88, 17.00]	15.21 [14.22, 16.27]	14.78 [13.79, 15.86]	14.06 [13.22, 15.23]
Arachidic acid (20:0)	0.43 [0.36, 0.49]	0.43 [0.38, 0.49]	0.51 [0.45, 0.58]	0.48 [0.42, 0.56]	0.44 [0.39, 0.50]
Heneicosanoic acid (21:0)	0.07 [0.00, 0.10]	0.00 [0.00, 0.04]	0.00 [0.00, 0.00]	0.00 [0.00, 0.06]	0.07 [0.00, 0.10]
Behenic acid (22:0)	1.76 [1.40, 2.13]	1.41 [1.18, 1.65]	1.46 [1.23, 1.69]	1.95 [1.49, 2.52]	2.05 [1.58, 2.45]
Tricosanoic acid (23:0)	0.32 [0.27, 0.38]	0.58 [0.49, 0.64]	0.38 [0.31, 0.46]	0.38 [0.31, 0.45]	0.35 [0.30, 0.41]
Lignoceric acid (24:0)	2.21 [1.86, 2.56]	0.68 [0.52, 0.88]	0.66 [0.54, 0.82]	2.45 [2.05, 2.84]	2.44 [1.97, 2.82]
Total MUFA	24.38 [22.69, 26.18]	24.37 [22.86, 26.30]	24.76 [23.13, 26.59]	22.93 [21.39, 24.76]	21.81 [20.00, 23.40]
Myristoleic acid (14:1)	0.00 [0.00, 0.00]	0.00 [0.00, 0.00]	0.00 [0.00, 0.00]	0.00 [0.00, 0.00]	0.00 [0.00, 0.00]
Pentadecenoic acid (15:1)	0.80 [0.65, 0.93]	0.00 [0.00, 0.05]	0.86 [0.73, 1.02]	0.87 [0.72, 1.01]	0.87 [0.74, 1.02]
Palmitoleic acid (16:1)	1.00 [0.82, 1.28]	0.85 [0.62, 1.07]	0.80 [0.64, 1.05]	0.66 [0.53, 0.85]	0.64 [0.50, 0.78]
Heptadecenoic acid (17:1)	1.18 [0.92, 1.37]	0.37 [0.32, 0.44]	1.32 [1.11, 1.55]	1.35 [1.11, 1.56]	1.36 [1.13, 1.58]
Elaidic acid (18:1n-9 *trans*)	0.12 [0.10, 0.15]	0.09 [0.07, 0.13]	0.00 [0.00, 0.09]	0.10 [0.00, 0.14]	0.12 [0.09, 0.14]
Oleic acid (18:1n-9 *cis*)	18.06 [16.41, 20.05]	18.94 [17.50, 20.38]	17.58 [16.23, 19.59]	16.33 [14.99, 18.01]	15.44 [14.07, 17.04]
Gondoic acid (20:1n-9)	0.30 [0.26, 0.35]	0.31 [0.26, 0.39]	0.36 [0.31, 0.40]	0.33 [0.28, 0.39]	0.28 [0.24, 0.33]
Erucic acid (22:1)	0.44 [0.30, 0.65]	0.96 [0.59, 1.51]	0.74 [0.53, 1.17]	0.57 [0.37, 0.86]	0.36 [0.25, 0.51]
Nervonic acid (24:1n-9)	2.22 [1.81, 2.62]	2.68 [2.00, 3.10]	2.55 [2.07, 3.03]	2.51 [2.05, 3.04]	2.47 [2.00, 2.92]

Table S5. Baseline Whole-Blood Fatty Acid Profiles and Derived Indices Across Phenotypes (Continued)

Fatty Acids and Derived Indices	Phenotype 1 (Moderate SFA, *n* = 821)	Phenotype 2 (High-SFA/low-PUFA, *n* = 259)	Phenotype 3 (Intermediate SFA-PUFA, *n* = 387)	Phenotype 4 (Moderate PUFA, *n* = 725)	Phenotype 5 (High PUFA, *n* = 817)
Total n-3 PUFA	3.27 [2.69, 3.98]	2.79 [2.22, 3.50]	3.44 [2.86, 4.04]	4.37 [3.45, 5.34]	5.11 [4.33, 6.12]
α-Linolenic acid (ALA, 18:3n-3)	0.29 [0.23, 0.40]	0.31 [0.24, 0.40]	0.32 [0.25, 0.42]	0.32 [0.23, 0.42]	0.31 [0.24, 0.41]
Eicosatrienoic acid (ETE, 20:3n-3)	0.00 [0.00, 0.00]	0.00 [0.00, 0.00]	0.00 [0.00, 0.00]	0.00 [0.00, 0.00]	0.00 [0.00, 0.00]
Eicosapentaenoic acid (EPA, 20:5n-3)	0.31 [0.20, 0.49]	0.23 [0.18, 0.31]	0.40 [0.22, 0.64]	0.46 [0.26, 0.90]	0.41 [0.28, 0.76]
Docosapentaenoic acid (DPA, 22:5n-3)	0.83 [0.65, 1.04]	0.70 [0.40, 0.98]	0.89 [0.69, 1.08]	1.05 [0.84, 1.34]	1.33 [1.11, 1.60]
Docosahexaenoic acid (DHA, 22:6n-3)	1.68 [1.30, 2.11]	1.43 [1.15, 1.89]	1.67 [1.32, 2.10]	2.27 [1.75, 2.92]	2.79 [2.27, 3.48]
Total n-6 PUFA	25.48 [23.75, 27.14]	25.37 [23.52, 27.01]	27.04 [25.50, 28.54]	27.48 [25.57, 29.57]	30.03 [28.04, 32.15]
Linolelaidic acid (18:2n-6 *trans*)	1.60 [1.43, 1.77]	1.67 [1.53, 1.82]	1.56 [1.40, 1.75]	1.62 [1.45, 1.83]	1.49 [1.34, 1.66]
Linoleic acid (LA, 18:2n-6 *cis*)	16.77 [15.34, 18.17]	16.17 [15.07, 17.23]	16.99 [15.69, 18.04]	17.77 [16.35, 19.08]	17.92 [16.30, 19.40]
γ-Linolenic acid (GLA, 18:3n-6)	0.16 [0.11, 0.22]	0.11 [0.00, 0.17]	0.00 [0.00, 0.13]	0.00 [0.00, 0.00]	0.14 [0.10, 0.21]
Dihomo-γ-linolenic acid (DGLA, 20:3n-6)	1.13 [0.95, 1.32]	1.02 [0.83, 1.18]	1.08 [0.93, 1.32]	1.13 [0.95, 1.36]	1.32 [1.12, 1.53]
Arachidonic acid (AA, 20:4n-6)	6.06 [5.18, 7.09]	5.28 [4.33, 6.18]	6.21 [5.25, 7.35]	7.30 [6.09, 8.63]	9.04 [7.90, 10.44]
Docosadienoic acid (22:2n-6)	0.09 [0.00, 0.14]	0.00 [0.00, 0.00]	0.00 [0.00, 0.00]	0.00 [0.00, 0.07]	0.00 [0.00, 0.11]
Adrenic acid (22:4n-6)	0.72 [0.58, 0.87]	0.22 [0.14, 0.30]	0.27 [0.16, 0.34]	0.87 [0.69, 1.05]	1.01 [0.85, 1.22]
Osbond acid (22:5n-6)	0.29 [0.21, 0.37]	2.29 [1.85, 2.75]	2.32 [1.90, 2.66]	0.36 [0.24, 0.47]	0.45 [0.35, 0.57]
Eicosadienoic acid (20:2)	0.35 [0.31, 0.40]	0.34 [0.30, 0.38]	0.35 [0.29, 0.40]	0.40 [0.34, 0.45]	0.40 [0.36, 0.45]
EPA + DHA	2.06 [1.62, 2.61]	1.70 [1.37, 2.21]	2.12 [1.71, 2.65]	2.88 [2.16, 3.69]	3.36 [2.69, 4.23]
AA/EPA ratio	20.00 [12.04, 30.05]	21.90 [16.31, 28.92]	15.48 [9.77, 26.18]	15.47 [7.54, 27.81]	22.16 [11.33, 32.73]
n-6/n-3 ratio	7.80 [6.63, 9.27]	8.99 [7.35, 11.31]	7.97 [6.82, 9.35]	6.34 [5.30, 7.66]	5.95 [5.04, 6.84]

Abbreviations: AA, arachidonic acid; DHA, docosahexaenoic acid; EPA, eicosapentaenoic acid; IQR, interquartile range; MUFA, monounsaturated fatty acid; PUFA, polyunsaturated fatty acid; SFA, saturated fatty acid.

Note: Data are presented as median (IQR). Column headers denote the 5 whole-blood fatty acid phenotypes identified via unsupervised K-means clustering within the baseline cross-sectional sample (N = 3009). In the "Fatty Acids and Derived Indices" column, individual and composite fatty acids, alongside EPA + DHA, are expressed as relative abundances (percentages of the total measured whole-blood fatty acid pool). The derived AA/EPA and n-6/n-3 ratios are calculated as absolute mathematical quotients and are unitless. Eicosadienoic acid (20:2) and *trans* fatty acids were analysed individually and were excluded from the composite calculations of PUFA or MUFA. To maintain computational stability during unsupervised dimensionality reduction, variables exhibiting zero values in greater than 50% of the baseline population—specifically heneicosanoic acid (21:0), myristoleic acid (14:1), eicosatrienoic acid (20:3n-3), and docosadienoic acid (22:2n-6)—were excluded from the initial principal component analysis and subsequent clustering algorithms. These excluded variables, alongside the derived ratios, are included in this table exclusively for comprehensive descriptive evaluation and phenotype characterisation.

Table S6. Bootstrap Stability of the Five Whole-Blood Fatty Acid Phenotypes

Phenotype	No. of Participants	Mean Jaccard Similarity
Phenotype 1 (moderate SFA)	821	0.965
Phenotype 2 (high SFA/low PUFA)	259	0.987
Phenotype 3 (intermediate SFA-PUFA)	387	0.973
Phenotype 4 (moderate PUFA)	725	0.999
Phenotype 5 (high PUFA)	817	0.965

Note: Cluster stability was assessed using 1,000 bootstrap resamples of the seven retained principal component analysis (PCA) scores. Five-cluster K-means clustering was refitted in each resample, and cluster-wise stability was quantified as the mean Jaccard similarity between each original phenotype and its best-matching bootstrap cluster. Using Jaccard similarity thresholds of ≤0.50 for dissolution and >0.75 for recovery, no phenotype was dissolved, and all five phenotypes were successfully recovered in all 1,000 bootstrap resamples. The clustering solution obtained from the full dataset within the stability procedure showed 100% agreement with the original phenotype assignments after label matching.

Table S7. Contextual Interpretation of Adjusted Baseline EORTC QLQ-C30 Differences Across Whole-Blood Fatty Acid Phenotypes

Variables	Maximum adjusted between-phenotype difference	Relative to 5-point contextual benchmark
EORTC QLQ-C30 Summary Score	1.81	<5
Global Health Status (QL)	3.05	<5
Physical functioning (PF)	2.17	<5
Role functioning (RF)	2.51	<5
Emotional functioning (EF)	1.00	<5
Cognitive functioning (CF)	2.73	<5
Social functioning (SF)	4.26	<5
Fatigue (FA)	2.50	<5
Nausea and vomiting (NV)	1.04	<5
Pain (PA)	2.58	<5
Dyspnoea (DY)	3.15	<5
Insomnia (SL)	2.56	<5
Appetite loss (AP)	1.40	<5
Constipation (CO)	2.68	<5
Diarrhoea (DI)	2.57	<5
Financial difficulties (FI)	2.74	<5

Abbreviations: EORTC QLQ-C30, European Organisation for Research and Treatment of Cancer Quality of Life Questionnaire Core 30; MID, minimally important difference.

Note: Maximum adjusted between-phenotype differences were calculated as the difference between the highest and lowest Model 2 estimated marginal means across the five whole-blood fatty acid phenotypes reported in Table 2. A 5-point absolute difference was used as the contextual benchmark for effect magnitude, representing the lower bound of the commonly used 5–10-point range for clinically important group-level EORTC QLQ-C30 differences. For the EORTC QLQ-C30 summary score, the 5-point benchmark was used for contextual interpretation because the cited MID guidance did not derive a summary-score-specific MID.

Table S8. Covariate-Adjusted Dietary Intake Profiles Across Whole-Blood Fatty Acid Phenotypes

Variables	Phenotype 1 (Moderate SFA, *n* = 755)	Phenotype 2 (High-SFA/low-PUFA, *n* = 241)	Phenotype 3 (Intermediate SFA-PUFA, *n* = 364)	Phenotype 4 (Moderate PUFA, *n* = 681)	Phenotype 5 (High PUFA, *n* = 771)	*P* value[a]
Rice	236.08 (224.26, 247.90)	227.15 (208.58, 245.72)	220.13 (204.45, 235.81)	216.28 (204.36, 228.19)	211.03 (199.04, 223.03)	0.007
Wheat-based foods	59.32 (54.76, 63.88)	55.79 (48.62, 62.96)	53.67 (47.62, 59.72)	67.39 (62.79, 71.99)	66.28 (61.65, 70.91)	<0.001
Whole grains	43.69 (38.70, 48.69)	50.39 (42.54, 58.24)	51.89 (45.26, 58.52)	49.57 (44.54, 54.61)	48.31 (43.24, 53.38)	0.17
Fried wheat foods	5.80 (4.73, 6.86)	6.91 (5.24, 8.59)	7.08 (5.67, 8.49)	5.63 (4.56, 6.71)	6.38 (5.30, 7.46)	0.49
Pork and pork products	56.15 (52.44, 59.86)	55.57 (49.74, 61.39)	56.46 (51.54, 61.38)	56.41 (52.67, 60.15)	55.10 (51.34, 58.86)	0.18
Beef, mutton, and other red meats	14.83 (12.74, 16.92)	23.42 (20.13, 26.70)	23.25 (20.48, 26.03)	15.67 (13.56, 17.78)	17.01 (14.89, 19.13)	<0.001
Poultry	23.69 (21.21, 26.16)	27.12 (23.23, 31.00)	27.12 (23.83, 30.40)	24.64 (22.14, 27.13)	26.24 (23.73, 28.75)	0.29
Seafood	53.71 (49.19, 58.22)	54.60 (47.51, 61.69)	57.35 (51.36, 63.34)	53.88 (49.33, 58.43)	47.84 (43.26, 52.42)	0.046
Eggs and egg products	75.12 (69.59, 80.65)	73.10 (64.41, 81.79)	73.55 (66.21, 80.88)	74.22 (68.65, 79.80)	75.61 (70.00, 81.22)	0.41
Light-colored vegetables	340.85 (319.27, 362.42)	331.76 (297.85, 365.67)	335.56 (306.93, 364.19)	314.32 (292.57, 336.07)	323.27 (301.37, 345.16)	0.50
Dark-colored vegetables	131.65 (118.35, 144.95)	140.75 (119.85, 161.66)	145.64 (127.99, 163.29)	129.50 (116.09, 142.91)	129.10 (115.60, 142.60)	0.77
Soybean products	45.70 (41.72, 49.67)	41.01 (34.77, 47.26)	44.32 (39.05, 49.60)	46.12 (42.12, 50.13)	42.65 (38.62, 46.68)	0.08
Pickled and smoked foods	7.47 (6.09, 8.86)	7.79 (5.62, 9.97)	9.01 (7.17, 10.84)	7.48 (6.09, 8.88)	6.80 (5.39, 8.20)	0.50
Fresh fruits	148.01 (137.19, 158.84)	157.33 (140.32, 174.34)	157.75 (143.38, 172.11)	140.69 (129.78, 151.61)	149.33 (138.34, 160.31)	0.37
Nuts and seeds	24.60 (22.34, 26.86)	26.21 (22.65, 29.76)	25.21 (22.21, 28.21)	23.92 (21.64, 26.20)	24.25 (21.96, 26.54)	0.39
Dairy products	176.95 (166.34, 187.57)	163.35 (146.67, 180.04)	164.30 (150.21, 178.39)	176.73 (166.03, 187.43)	168.68 (157.90, 179.45)	0.08
Drinking water	1166.18 (1112.51, 1219.86)	1230.86 (1146.50, 1315.21)	1256.86 (1185.63, 1328.09)	1167.70 (1113.58, 1221.81)	1178.91 (1124.43, 1233.38)	0.10
Cooking oil	36.49 (35.04, 37.94)	35.69 (33.41, 37.97)	35.71 (33.78, 37.63)	37.61 (36.15, 39.07)	39.01 (37.54, 40.49)	<0.001
Salt	5.61 (5.31, 5.91)	5.23 (4.75, 5.71)	5.24 (4.84, 5.65)	5.54 (5.23, 5.84)	5.47 (5.16, 5.78)	0.30
Added sugar	4.71 (4.25, 5.17)	3.81 (3.08, 4.54)	4.55 (3.93, 5.17)	4.74 (4.28, 5.21)	4.51 (4.04, 4.97)	0.09

Table S8. Covariate-Adjusted Dietary Intake Profiles Across Whole-Blood Fatty Acid Phenotypes (*N* = 2812) (Continued)

Variables	Phenotype 1 (Moderate SFA, *n* = 755)	Phenotype 2 (High-SFA/low-PUFA, *n* = 241)	Phenotype 3 (Intermediate SFA-PUFA, *n* = 364)	Phenotype 4 (Moderate PUFA, *n* = 681)	Phenotype 5 (High PUFA, *n* = 771)	*P* value[a]
Dietary Variety(n)	12.85 (12.68, 13.02)	13.37 (13.10, 13.65)	13.24 (13.01, 13.47)	12.85 (12.67, 13.02)	12.98 (12.81, 13.16)	<0.001
Total energy intake (kcal/d)	1746.71 (1698.74, 1794.68)	1758.73 (1683.34, 1834.12)	1649.30 (1585.68, 1712.92)	1771.31 (1722.97, 1819.64)	1743.12 (1694.43, 1791.81)	0.008

Abbreviations: ANCOVA, analysis of covariance; CI, confidence interval; EMM, estimated marginal mean.

Note: Data are presented as covariate-adjusted estimated marginal means (95% CIs). Column headers denote the 5 whole-blood fatty acid phenotypes identified via unsupervised *K*-means clustering. The analytical sample was restricted to participants with valid dietary assessments and plausible total energy intakes (*N* = 2812). With the exception of total energy intake (expressed in kcal/d) and dietary variety (quantified as an aggregate count of reported food items with non-zero intake; range, 0–16), all dietary variables are expressed in grams per day (g/d). Individual dietary components were adjusted for age, sex, cancer type, and total energy intake to account for absolute consumption volume. Both total energy intake and dietary variety were adjusted exclusively for age, sex, and cancer type.

[a] Overall statistical significance across the 5 phenotypes was determined via analysis of covariance (ANCOVA). *P* values reflect the omnibus test results.

Table S9. Covariate-Adjusted Chinese Dietary Balance Index-22 (DBI-22) Component Scores Across Whole-Blood Fatty Acid Phenotypes

Variables	Phenotype 1 (Moderate SFA, *n* = 755)	Phenotype 2 (High-SFA/low-PUFA, *n* = 241)	Phenotype 3 (Intermediate SFA-PUFA, *n* = 364)	Phenotype 4 (Moderate PUFA, *n* = 681)	Phenotype 5 (High PUFA, *n* = 771)	*P* value[a]
High Bound Score (HBS)	8.67 (8.27, 9.06)	8.87 (8.22, 9.52)	7.89 (7.35, 8.43)	8.82 (8.41, 9.23)	9.11 (8.71, 9.52)	0.003
Low Bound Score (LBS)	17.51 (16.82, 18.21)	16.63 (15.50, 17.76)	17.67 (16.72, 18.61)	17.18 (16.46, 17.90)	17.66 (16.94, 18.37)	0.60
Diet Quality Distance (DQD)	26.18 (25.48, 26.88)	25.50 (24.36, 26.64)	25.56 (24.60, 26.51)	26.01 (25.28, 26.73)	26.77 (26.05, 27.49)	0.16
Cereal	−1.74 (−2.11, −1.37)	−1.81 (−2.42, −1.20)	−2.43 (−2.94, −1.92)	−1.58 (−1.96, −1.19)	−1.90 (−2.28, −1.51)	0.07
Vegetable	−1.11 (−1.23, −0.99)	−1.02 (−1.22, −0.83)	−1.12 (−1.28, −0.95)	−1.20 (−1.33, −1.08)	−1.16 (−1.28, −1.03)	0.42
Fruit	−2.47 (−2.62, −2.32)	−2.35 (−2.60, −2.11)	−2.50 (−2.71, −2.30)	−2.62 (−2.78, −2.47)	−2.52 (−2.67, −2.37)	0.09
Dairy	−2.60 (−2.77, −2.43)	−2.75 (−3.02, −2.48)	−2.83 (−3.06, −2.61)	−2.54 (−2.71, −2.37)	−2.71 (−2.88, −2.54)	0.24
Soybean	−1.85 (−2.02, −1.68)	−1.77 (−2.06, −1.49)	−1.77 (−2.01, −1.54)	−1.79 (−1.97, −1.62)	−1.86 (−2.04, −1.68)	0.90
Red meat and products, poultry and game	2.65 (2.49, 2.81)	3.15 (2.88, 3.41)	2.67 (2.46, 2.89)	2.69 (2.52, 2.85)	2.84 (2.67, 3.00)	0.005
Fish and shrimp	−0.96 (−1.06, −0.86)	−0.89 (−1.05, −0.73)	−0.93 (−1.07, −0.79)	−0.90 (−1.00, −0.79)	−1.02 (−1.13, −0.92)	0.49
Egg	1.26 (1.07, 1.44)	1.33 (1.02, 1.63)	0.99 (0.74, 1.25)	1.32 (1.13, 1.51)	1.42 (1.23, 1.61)	0.07
Cooking oil	2.32 (2.14, 2.51)	2.32 (2.02, 2.63)	2.18 (1.93, 2.44)	2.46 (2.26, 2.65)	2.70 (2.51, 2.89)	0.002
Alcoholic beverage	0.00 (−0.00, 0.01)	0.01 (−0.00, 0.02)	0.02 (0.01, 0.03)	0.01 (0.00, 0.02)	0.01 (−0.00, 0.01)	0.20
Added sugar	0.03 (−0.00, 0.05)	0.00 (−0.04, 0.05)	0.07 (0.03, 0.11)	0.03 (−0.00, 0.06)	0.03 (−0.00, 0.06)	0.16
Salt	0.87 (0.75, 0.98)	0.72 (0.54, 0.90)	0.71 (0.56, 0.86)	0.88 (0.77, 1.00)	0.81 (0.69, 0.92)	0.17
Diet variety	−2.60 (−2.74, −2.45)	−2.32 (−2.56, −2.09)	−2.65 (−2.85, −2.45)	−2.49 (−2.64, −2.34)	−2.58 (−2.73, −2.43)	0.20
Drinking water	−2.65 (−2.87, −2.43)	−2.37 (−2.73, −2.01)	−2.19 (−2.49, −1.89)	−2.62 (−2.85, −2.39)	−2.60 (−2.83, −2.37)	0.045

Abbreviations: ANCOVA, analysis of covariance; CI, confidence interval; DBI-22, Chinese Dietary Balance Index-22; DQD, diet quality distance; HBS, high bound score; LBS, low bound score.

Note: Data are presented as covariate-adjusted estimated marginal means (95% CIs). Column headers denote the 5 whole-blood fatty acid phenotypes identified via unsupervised K-means clustering. The analytical sample was restricted to participants with valid dietary assessments and plausible total energy intakes ($N = 2812$). All

evaluated variables represent unitless component scores derived from the DBI-22 framework. Within this scoring system, negative values denote inadequate dietary intake (deficit), whereas positive values indicate excessive intake (surplus). Consistent with a hierarchical covariate adjustment strategy, DBI-22 indices (including HBS, LBS, DQD, and individual food group scores) were adjusted exclusively for cancer type, given that age, sex, and body weight parameters are intrinsically integrated into the DBI-22 scoring algorithm. Alcoholic beverage scores were estimated based on consumption frequency rather than quantitative volumetric assessments owing to questionnaire limitations.

[a] Overall statistical significance across the 5 phenotypes was determined via analysis of covariance (ANCOVA). *P* values reflect the omnibus test results.

Table S10. Covariate-Adjusted Pass Rates for Nutrition Literacy Domains Across Whole-Blood Fatty Acid Phenotypes

**Nutrition Literacy Domains**	**Phenotype 1 (Moderate SFA, *n* = 755)**	**Phenotype 2 (High-SFA/low-PUFA, *n* = 241)**	**Phenotype 3 (Intermediate SFA-PUFA, *n* = 364)**	**Phenotype 4 (Moderate PUFA, *n* = 681)**	**Phenotype 5 (High PUFA, *n* = 771)**	***P* value[a]**
Functional pass rate (%)	43.0 (38.8, 47.3)	43.4 (36.7, 50.0)	44.2 (38.5, 49.8)	43.8 (39.4, 48.1)	45.7 (41.3, 50.1)	0.88
Information pass rate (%)	45.3 (41.0, 49.6)	47.9 (41.1, 54.7)	50.1 (44.4, 55.9)	46.5 (42.1, 50.8)	45.2 (40.9, 49.6)	0.56
Application pass rate (%)	27.4 (23.6, 31.3)	31.7 (25.5, 37.9)	29.6 (24.4, 34.8)	30.6 (26.6, 34.6)	28.7 (24.8, 32.6)	0.63
Calculation pass rate (%)	26.3 (22.5, 30.0)	28.4 (22.3, 34.5)	27.3 (22.2, 32.4)	31.2 (27.1, 35.3)	29.9 (25.8, 33.9)	0.27
Nutrition Literacy pass rate (%)	40.3 (36.1, 44.6)	41.4 (34.7, 48.2)	39.8 (34.1, 45.5)	45.0 (40.6, 49.4)	41.0 (36.6, 45.3)	0.39

Abbreviations: CI, confidence interval; PUFA, polyunsaturated fatty acid; SFA, saturated fatty acid.

Note: Data are presented as covariate-adjusted estimated marginal probabilities (95% CIs) expressed as percentages. Column headers denote the 5 whole-blood fatty acid phenotypes identified via unsupervised K-means clustering. The analytical sample was restricted to participants with valid dietary assessments (*n* = 2812). Binary outcomes (pass rates across nutrition literacy domains) were evaluated using multivariable logistic regression models. To preserve upstream sociodemographic variance, all evaluated outcomes were adjusted exclusively for age, sex, and cancer type.

[a] Overall statistical significance across the 5 phenotypes was determined via Wald χ^2 tests, with *P* values reflecting omnibus test results.

Table S11. Comparison of Baseline Characteristics Between the Overlapping Longitudinal Subcohort and the Exclusively Cross-sectional Cohort

**Characteristics**	**Overall**	**Exclusively Cross-sectional Cohort (*n* = 2586)**	**Overlapping Longitudinal Subcohort (*n* = 423)**	***P* value[a]**
**Age group, y (*n* = 3009)**				<0.001
<60	746 (24.8)	599 (23.2)	147 (34.8)	
60–64	634 (21.1)	532 (20.6)	102 (24.1)	
65–69	911 (30.3)	799 (30.9)	112 (26.5)	
≥70	718 (23.9)	656 (25.4)	62 (14.7)	
**Sex (*n* = 3009)**				0.02
Male	612 (20.3)	507 (19.6)	105 (24.8)	
Female	2397 (79.7)	2079 (80.4)	318 (75.2)	
**Monthly personal income, CNY (*n* = 2984)**				<0.001
≤1000	256 (8.6)	206 (8.0)	50 (11.9)	
1001–3000	967 (32.4)	805 (31.4)	162 (38.5)	
3001–5000	1275 (42.7)	1140 (44.5)	135 (32.1)	
>5000	486 (16.3)	412 (16.1)	74 (17.6)	
**Education (*n* = 2818)**				0.14
Primary school or less	248 (8.8)	212 (8.7)	36 (9.2)	
Middle school	1368 (48.5)	1175 (48.5)	193 (49.1)	
High school	901 (32.0)	790 (32.6)	111 (28.2)	
College or higher	301 (10.7)	248 (10.2)	53 (13.5)	
**Physical activity (*n* = 2915)**				0.01
5–7 times per week	880 (30.2)	744 (29.7)	136 (32.9)	
3–4 times per week	882 (30.3)	738 (29.5)	144 (34.8)	
1–2 times per week	715 (24.5)	633 (25.3)	82 (19.8)	
<1 time per week	438 (15.0)	386 (15.4)	52 (12.6)	
**Smoking status (*n* = 2922)**				0.38
Never smoker	2641 (90.4)	2253 (90.1)	388 (92.2)	
Former smoker	187 (6.4)	166 (6.6)	21 (5.0)	
Current smoker	94 (3.2)	82 (3.3)	12 (2.9)	
**Drinking status (*n* = 2895)**				0.48
Never drinker	2475 (85.5)	2121 (85.7)	354 (84.3)	
Former drinker	145 (5.0)	119 (4.8)	26 (6.2)	
Current drinker	275 (9.5)	235 (9.5)	40 (9.5)	

Table S11. Comparison of Baseline Characteristics Between the Overlapping Longitudinal Subcohort and the Exclusively Cross-sectional Cohort (Continued)

**Characteristics**	**Overall**	**Exclusively Cross-sectional Cohort (*n* = 2586)**	**Overlapping Longitudinal Subcohort (*n* = 423)**	***P* value**
**Living arrangement (*n* = 2937)**				0.21
With spouse	1346 (45.8)	1163 (46.2)	183 (43.8)	
With family	225 (7.7)	190 (7.5)	35 (8.4)	
With spouse and family	1117 (38.0)	944 (37.5)	173 (41.4)	
Live alone	249 (8.5)	222 (8.8)	27 (6.5)	
**Body mass index (*n* = 2980)**				0.56
Underweight	104 (3.5)	93 (3.6)	11 (2.6)	
Normal	1561 (52.4)	1350 (52.7)	211 (50.6)	
Overweight	1014 (34.0)	864 (33.7)	150 (36.0)	
Obese	301 (10.1)	256 (10.0)	45 (10.8)	
**Number of comorbidities (*n* = 3004)**				<0.001
0	883 (29.4)	703 (27.2)	180 (42.7)	
1	952 (31.7)	832 (32.2)	120 (28.4)	
2	586 (19.5)	510 (19.8)	76 (18.0)	
≥3	583 (19.4)	537 (20.8)	46 (10.9)	
**Duration of cancer survivorship, y (*n* = 2977)**				<0.001
<5	350 (11.8)	253 (9.9)	97 (23.0)	
5–10	1115 (37.5)	862 (33.7)	253 (60.1)	
≥10	1512 (50.8)	1441 (56.4)	71 (16.9)	
**Disease trajectory (*n* = 3007)**				<0.001
Cured	1069 (35.6)	916 (35.4)	153 (36.2)	
Well-controlled	608 (20.2)	549 (21.2)	59 (13.9)	
Stable	987 (32.8)	817 (31.6)	170 (40.2)	
Worsened	27 (0.9)	24 (0.9)	3 (0.7)	
Unknown	316 (10.5)	278 (10.8)	38 (9.0)	
**Relapse (*n* = 3008)**				0.053
Yes	238 (7.9)	215 (8.3)	23 (5.4)	
No	2770 (92.1)	2370 (91.7)	400 (94.6)	
**Metastasis (*n* = 3005)**				0.06
Yes	144 (4.8)	132 (5.1)	12 (2.8)	
No	2861 (95.2)	2450 (94.9)	411 (97.2)	

Table S11. Comparison of Baseline Characteristics Between the Overlapping Longitudinal Subcohort and the Exclusively Cross-sectional Cohort (Continued)

**Characteristics**	**Overall**	**Exclusively Cross-sectional Cohort (*n* = 2586)**	**Overlapping Longitudinal Subcohort (*n* = 423)**	***P* value**
**Cancer type (*n* = 3009)**				<0.001
Hematologic	73 (2.4)	65 (2.5)	8 (1.9)	
Breast	1113 (37.0)	987 (38.2)	126 (29.8)	
Uterine and cervical	137 (4.6)	127 (4.9)	10 (2.4)	
Colorectal	301 (10.0)	251 (9.7)	50 (11.8)	
Genitourinary	120 (4.0)	106 (4.1)	14 (3.3)	
Head and neck	54 (1.8)	47 (1.8)	7 (1.7)	
Hepatopancreatobiliary	66 (2.2)	58 (2.2)	8 (1.9)	
Lung	361 (12.0)	258 (10.0)	103 (24.3)	
Ovarian	80 (2.7)	72 (2.8)	8 (1.9)	
Thyroid	186 (6.2)	165 (6.4)	21 (5.0)	
Upper gastrointestinal	218 (7.2)	182 (7.0)	36 (8.5)	
Multiple primary cancers	262 (8.7)	234 (9.0)	28 (6.6)	
Others	38 (1.3)	34 (1.3)	4 (0.9)	
**Omega-3 supplement use (*n* = 2925)**				0.02
Regular user	373 (12.8)	313 (12.4)	60 (14.6)	
Occasional user	466 (15.9)	419 (16.7)	47 (11.5)	
Non-user	2086 (71.3)	1783 (70.9)	303 (73.9)	
**Severity of COVID-19 (*n* = 2976)**				0.17
Uninfected	502 (16.9)	416 (16.3)	86 (20.5)	
Asymptomatic	82 (2.8)	70 (2.7)	12 (2.9)	
Mild	1750 (58.8)	1511 (59.1)	239 (57.0)	
Moderate	598 (20.1)	519 (20.3)	79 (18.9)	
Severe or critical	44 (1.5)	41 (1.6)	3 (0.7)	
**Time since COVID-19 (*n* = 2396)**				>0.99
≤1 month	65 (2.7)	56 (2.7)	9 (2.8)	
>1 month	2331 (97.3)	2017 (97.3)	314 (97.2)	

Abbreviations: BMI, body mass index; CNY, Chinese yuan; COVID-19, coronavirus disease 2019.

Note: Data are presented as No. (%). Denominators for individual characteristics vary due to missing data; percentages are derived from available cases within each category. The denominator for time since COVID-19 infection was restricted to participants with a documented infection history.

[a] *P* values were calculated using Pearson χ^2 tests or Fisher exact tests, as appropriate, to evaluate baseline homogeneity between participants enrolled in the longitudinal subcohort and those retained exclusively for cross-sectional analysis.

Table S12. Baseline Characteristics of Cancer Survivors Across Whole-Blood Fatty Acid Phenotypes

**Characteristics**	**Overall**	**Phenotype 1 (*n* = 821)**	**Phenotype 2 (*n* = 259)**	**Phenotype 3 (*n* = 387)**	**Phenotype 4 (*n* = 725)**	**Phenotype 5 (*n* = 817)**	***P* value**
**Age group, y (*n* = 3009)**							
<60	746 (24.8)	202 (24.6)	79 (30.5)	97 (25.1)	166 (22.9)	202 (24.7)	
60–64	634 (21.1)	188 (22.9)	48 (18.5)	83 (21.4)	145 (20.0)	170 (20.8)	0.08
65–69	911 (30.3)	231 (28.1)	71 (27.4)	107 (27.6)	225 (31.0)	277 (33.9)	
≥70	718 (23.9)	200 (24.4)	61 (23.6)	100 (25.8)	189 (26.1)	168 (20.6)	
**Sex (*n* = 3009)**							
Male	612 (20.3)	137 (16.7)	56 (21.6)	73 (18.9)	202 (27.9)	144 (17.6)	<0.001
Female	2397 (79.7)	684 (83.3)	203 (78.4)	314 (81.1)	523 (72.1)	673 (82.4)	
**Monthly personal income, CNY (*n* = 2984)**							
≤1000	256 (8.6)	79 (9.8)	19 (7.4)	23 (6.0)	79 (10.9)	56 (6.9)	
1001–3000	967 (32.4)	289 (35.7)	92 (35.7)	126 (32.6)	211 (29.2)	249 (30.8)	0.002
3001–5000	1275 (42.7)	337 (41.6)	98 (38.0)	176 (45.6)	298 (41.3)	366 (45.3)	
>5000	486 (16.3)	105 (13.0)	49 (19.0)	61 (15.8)	134 (18.6)	137 (17.0)	
**Education (*n* = 2818)**							
Primary school or less	248 (8.8)	58 (7.6)	27 (11.1)	48 (13.3)	58 (8.6)	57 (7.3)	
Middle school	1368 (48.5)	388 (51.1)	106 (43.6)	160 (44.4)	339 (50.0)	375 (48.2)	0.009
High school	901 (32.0)	244 (32.1)	86 (35.4)	101 (28.1)	214 (31.6)	256 (32.9)	
College or higher	301 (10.7)	69 (9.1)	24 (9.9)	51 (14.2)	67 (9.9)	90 (11.6)	

Table S12. Baseline Characteristics of Cancer Survivors Across Whole-Blood Fatty Acid Phenotypes (Continued)

Characteristics	Overall	Phenotype 1 (*n* = 821)	Phenotype 2 (*n* = 259)	Phenotype 3 (*n* = 387)	Phenotype 4 (*n* = 725)	Phenotype 5 (*n* = 817)	*P* value
**Physical activity (*n* = 2915)**							<0.001
5–7 times per week	880 (30.2)	192 (24.3)	89 (36.0)	127 (34.0)	213 (30.2)	259 (32.5)	
3–4 times per week	882 (30.3)	234 (29.6)	84 (34.0)	109 (29.1)	218 (30.9)	237 (29.7)	
1–2 times per week	715 (24.5)	226 (28.6)	48 (19.4)	94 (25.1)	171 (24.2)	176 (22.1)	
<1 time per week	438 (15.0)	139 (17.6)	26 (10.5)	44 (11.8)	104 (14.7)	125 (15.7)	
**Smoking status (*n* = 2922)**							<0.001
Never smoker	2641 (90.4)	737 (92.8)	223 (88.5)	341 (91.2)	621 (87.7)	719 (90.6)	
Former smoker	187 (6.4)	31 (3.9)	17 (6.7)	19 (5.1)	73 (10.3)	47 (5.9)	
Current smoker	94 (3.2)	26 (3.3)	12 (4.8)	14 (3.7)	14 (2.0)	28 (3.5)	
**Drinking status (*n* = 2895)**							0.002
Never drinker	2475 (85.5)	685 (87.2)	206 (82.4)	331 (89.9)	569 (81.1)	684 (86.7)	
Former drinker	145 (5.0)	31 (3.9)	12 (4.8)	14 (3.8)	49 (7.0)	39 (4.9)	
Current drinker	275 (9.5)	70 (8.9)	32 (12.8)	23 (6.2)	84 (12.0)	66 (8.4)	
**Living arrangement (*n* = 2937)**							0.02
With spouse	1346 (45.8)	369 (46.3)	98 (38.4)	159 (41.8)	350 (49.5)	370 (46.4)	
With family	225 (7.7)	50 (6.3)	18 (7.1)	41 (10.8)	52 (7.4)	64 (8.0)	
With spouse and family	1117 (38.0)	308 (38.6)	119 (46.7)	150 (39.5)	254 (35.9)	286 (35.8)	
Live alone	249 (8.5)	70 (8.8)	20 (7.8)	30 (7.9)	51 (7.2)	78 (9.8)	

Table S12. Baseline Characteristics of Cancer Survivors Across Whole-Blood Fatty Acid Phenotypes (Continued)

**Characteristics**	**Overall**	**Phenotype 1 (*n* = 821)**	**Phenotype 2 (*n* = 259)**	**Phenotype 3 (*n* = 387)**	**Phenotype 4 (*n* = 725)**	**Phenotype 5 (*n* = 817)**	***P* value**
**Body mass index (*n* = 2980)**							<0.001
Underweight	104 (3.5)	20 (2.5)	10 (3.9)	11 (2.9)	38 (5.3)	25 (3.1)	
Normal	1561 (52.4)	380 (46.6)	147 (56.8)	243 (63.6)	387 (54.4)	404 (49.8)	
Overweight	1014 (34.0)	318 (39.0)	77 (29.7)	96 (25.1)	223 (31.3)	300 (36.9)	
Obese	301 (10.1)	97 (11.9)	25 (9.7)	32 (8.4)	64 (9.0)	83 (10.2)	
**Number of comorbidities (*n* = 3004)**							0.87
0	883 (29.4)	226 (27.6)	76 (29.5)	114 (29.5)	231 (32.0)	236 (28.9)	
1	952 (31.7)	274 (33.4)	84 (32.6)	118 (30.5)	220 (30.4)	256 (31.4)	
2	586 (19.5)	152 (18.5)	53 (20.5)	78 (20.2)	143 (19.8)	160 (19.6)	
≥3	583 (19.4)	168 (20.5)	45 (17.4)	77 (19.9)	129 (17.8)	164 (20.1)	
**Duration of cancer survivorship, y (*n* = 2977)**							<0.001
<5	350 (11.8)	85 (10.5)	17 (6.6)	31 (8.1)	94 (13.1)	123 (15.1)	
5–10	1115 (37.5)	327 (40.3)	90 (35.2)	144 (37.6)	271 (37.9)	283 (34.9)	
≥10	1512 (50.8)	399 (49.2)	149 (58.2)	208 (54.3)	350 (49.0)	406 (50.0)	
**Disease trajectory (*n* = 3007)**							0.99
Cured	1069 (35.6)	291 (35.4)	100 (38.6)	131 (33.9)	257 (35.4)	290 (35.5)	
Well-controlled	608 (20.2)	164 (20.0)	48 (18.5)	84 (21.8)	147 (20.3)	165 (20.2)	
Stable	987 (32.8)	275 (33.5)	81 (31.3)	119 (30.8)	244 (33.7)	268 (32.8)	
Worsened	27 (0.9)	7 (0.9)	1 (0.4)	5 (1.3)	7 (1.0)	7 (0.9)	
Unknown	316 (10.5)	84 (10.2)	29 (11.2)	47 (12.2)	70 (9.7)	86 (10.5)	

Table S12. Baseline Characteristics of Cancer Survivors Across Whole-Blood Fatty Acid Phenotypes (Continued)

**Characteristics**	**Overall**	**Phenotype 1 (*n* = 821)**	**Phenotype 2 (*n* = 259)**	**Phenotype 3 (*n* = 387)**	**Phenotype 4 (*n* = 725)**	**Phenotype 5 (*n* = 817)**	***P* value**
**Relapse (*n* = 3008)**							
Yes	238 (7.9)	52 (6.3)	20 (7.7)	45 (11.6)	49 (6.8)	72 (8.8)	0.02
No	2770 (92.1)	769 (93.7)	239 (92.3)	342 (88.4)	675 (93.2)	745 (91.2)	
**Metastasis (*n* = 3005)**							
Yes	144 (4.8)	31 (3.8)	12 (4.6)	29 (7.5)	30 (4.1)	42 (5.1)	0.06
No	2861 (95.2)	790 (96.2)	247 (95.4)	356 (92.5)	694 (95.9)	774 (94.9)	
**Severity of COVID-19 (*n* = 2976)**							
Uninfected	502 (16.9)	145 (18.0)	25 (9.8)	47 (12.2)	147 (20.5)	138 (17.0)	<0.001
Asymptomatic	82 (2.8)	29 (3.6)	5 (2.0)	10 (2.6)	20 (2.8)	18 (2.2)	
Mild	1750 (58.8)	457 (56.7)	179 (70.2)	251 (65.0)	389 (54.2)	474 (58.4)	
Moderate	598 (20.1)	162 (20.1)	43 (16.9)	71 (18.4)	150 (20.9)	172 (21.2)	
Severe or critical	44 (1.5)	13 (1.6)	3 (1.2)	7 (1.8)	12 (1.7)	9 (1.1)	
**Time since COVID-19 (*n* = 2396)**							0.14
≤1 month	65 (2.7)	21 (3.3)	6 (2.7)	9 (2.7)	20 (3.6)	9 (1.4)	
>1 month	2331 (97.3)	619 (96.7)	217 (97.3)	322 (97.3)	532 (96.4)	641 (98.6)	
**Omega-3 supplement use (*n* = 2925)**							
Regular user	373 (12.8)	93 (11.7)	36 (14.5)	53 (14.0)	87 (12.3)	104 (13.1)	0.74
Occasional user	466 (15.9)	126 (15.8)	46 (18.5)	52 (13.8)	117 (16.5)	125 (15.7)	
Non-user	2086 (71.3)	578 (72.5)	167 (67.1)	273 (72.2)	503 (71.1)	565 (71.2)	

Table S12. Baseline Characteristics of Cancer Survivors Across Whole-Blood Fatty Acid Phenotypes (Continued)

Characteristics	Overall	Phenotype 1 (*n* = 821)	Phenotype 2 (*n* = 259)	Phenotype 3 (*n* = 387)	Phenotype 4 (*n* = 725)	Phenotype 5 (*n* = 817)	*P* value
**Cancer type (*n* = 3009)**							<0.001
Hematologic	73 (2.4)	27 (3.3)	6 (2.3)	11 (2.8)	21 (2.9)	8 (1.0)	
Breast	1113 (37.0)	318 (38.7)	93 (35.9)	148 (38.2)	231 (31.9)	323 (39.5)	
Uterine and cervical	137 (4.6)	49 (6.0)	6 (2.3)	19 (4.9)	27 (3.7)	36 (4.4)	
Colorectal	301 (10.0)	74 (9.0)	20 (7.7)	40 (10.3)	79 (10.9)	88 (10.8)	
Genitourinary	120 (4.0)	37 (4.5)	12 (4.6)	14 (3.6)	35 (4.8)	22 (2.7)	
Head and neck	54 (1.8)	15 (1.8)	6 (2.3)	4 (1.0)	15 (2.1)	14 (1.7)	
Hepatopancreatobiliary	66 (2.2)	11 (1.3)	6 (2.3)	7 (1.8)	24 (3.3)	18 (2.2)	
Lung	361 (12.0)	78 (9.5)	32 (12.4)	47 (12.1)	87 (12.0)	117 (14.3)	
Ovarian	80 (2.7)	22 (2.7)	6 (2.3)	13 (3.4)	16 (2.2)	23 (2.8)	
Thyroid	186 (6.2)	55 (6.7)	17 (6.6)	27 (7.0)	35 (4.8)	52 (6.4)	
Upper gastrointestinal	218 (7.2)	55 (6.7)	19 (7.3)	24 (6.2)	77 (10.6)	43 (5.3)	
Multiple primary cancers	262 (8.7)	64 (7.8)	32 (12.4)	29 (7.5)	67 (9.2)	70 (8.6)	
Others	38 (1.3)	16 (1.9)	4 (1.5)	4 (1.0)	11 (1.5)	3 (0.4)	

Abbreviations: CNY, Chinese yuan; COVID-19, coronavirus disease 2019.

Note: Data are presented as No. (%). Denominators for individual characteristics vary due to missing data; percentages are derived from available cases within each category. The denominator for time since COVID-19 infection was restricted to participants with a documented infection history. Statistical differences in categorical covariates across the 5 whole-blood fatty acid phenotypes were determined using Pearson χ^2 tests. Fisher exact tests were applied for sparse contingency tables containing expected cell counts of less than 5.

Table S13. Adjusted Annualised Changes in Quality of Life From Baseline to the 12-Month Follow-Up Across Whole-Blood Fatty Acid Phenotypes

Variables	Phenotype 1 (Moderate SFA, *n* = 104)	Phenotype 2 (High-SFA/low-PUFA, *n* = 23)	Phenotype 3 (Intermediate SFA-PUFA, *n* = 22)	Phenotype 4 (Moderate PUFA, *n* = 119)	Phenotype 5 (High PUFA, *n* = 130)	Model 1 *P* value (*N* = 423)	Model 2 *P* value (*N* = 398)	Model 3 *P* value (*N* = 395)
EORTC QLQ-C30 Summary Score	−0.91 (−4.70, 2.89)	−5.51 (−10.46, −0.56)	1.60 (−3.19, 6.40)	−1.00 (−4.56, 2.55)	−0.64 (−4.32, 3.04)	0.03	0.047	0.03
Global Health Status (QL)	−13.38 (−22.74, −4.02)	−16.17 (−28.41, −3.93)	−8.76 (−20.58, 3.06)	−15.54 (−24.30, −6.78)	−13.43 (−22.55, −4.32)	0.17	0.60	0.43
Physical functioning (PF)	0.97 (−4.20, 6.13)	−2.88 (−9.63, 3.86)	2.91 (−3.60, 9.42)	2.34 (−2.50, 7.17)	2.05 (−2.97, 7.06)	0.21	0.29	0.34
Role functioning (RF)	−0.70 (−5.63, 4.24)	−6.89 (−13.32, −0.46)	0.12 (−6.10, 6.34)	−2.00 (−6.63, 2.62)	−1.91 (−6.70, 2.87)	0.23	0.12	0.08
Emotional functioning (EF)	1.27 (−4.50, 7.04)	−2.09 (−9.60, 5.42)	5.16 (−2.13, 12.45)	−0.23 (−5.62, 5.16)	−0.21 (−5.79, 5.37)	0.30	0.24	0.17
Cognitive functioning (CF)	−1.20 (−8.18, 5.79)	−1.24 (−10.37, 7.88)	0.44 (−8.36, 9.24)	1.68 (−4.88, 8.23)	1.02 (−5.75, 7.80)	0.64	0.65	0.72
Social functioning (SF)	−3.28 (−10.32, 3.75)	−4.85 (−14.01, 4.30)	2.16 (−6.75, 11.08)	−3.69 (−10.28, 2.91)	1.00 (−5.81, 7.81)	0.02	0.04	0.06
Fatigue (FA)	2.78 (−4.75, 10.31)	8.80 (−1.02, 18.62)	1.15 (−8.34, 10.64)	2.67 (−4.38, 9.72)	3.53 (−3.77, 10.84)	0.36	0.49	0.41
Nausea and vomiting (NV)	1.26 (−1.56, 4.07)	1.18 (−2.49, 4.85)	1.23 (−2.30, 4.77)	1.46 (−1.18, 4.10)	1.45 (−1.27, 4.18)	0.76	>0.99	0.90
Pain (PA)	−0.37 (−6.58, 5.85)	7.30 (−0.81, 15.40)	−3.04 (−10.89, 4.81)	−1.04 (−6.86, 4.78)	−1.54 (−7.58, 4.49)	0.04	0.052	0.04
Dyspnoea (DY)	1.63 (−5.64, 8.89)	7.64 (−1.86, 17.13)	−2.11 (−11.25, 7.04)	0.70 (−6.10, 7.51)	0.10 (−6.93, 7.13)	0.21	0.23	0.14
Insomnia (SL)	−0.63 (−9.97, 8.70)	4.37 (−7.83, 16.57)	−3.05 (−14.79, 8.69)	−0.43 (−9.24, 8.38)	0.81 (−8.24, 9.87)	0.70	0.73	0.74
Appetite loss (AP)	4.14 (−1.55, 9.83)	5.50 (−1.88, 12.87)	5.88 (−1.24, 12.99)	3.21 (−2.10, 8.52)	5.43 (−0.07, 10.93)	0.76	0.62	0.64
Constipation (CO)	3.39 (−4.06, 10.84)	18.97 (9.26, 28.69)	1.49 (−7.84, 10.82)	6.81 (−0.14, 13.76)	4.42 (−2.77, 11.62)	<0.001	<0.001	<0.001
Diarrhoea (DI)	2.05 (−3.41, 7.51)	2.82 (−4.30, 9.93)	−1.92 (−8.80, 4.97)	2.24 (−2.87, 7.34)	1.95 (−3.34, 7.23)	0.54	0.62	0.61
Financial difficulties (FI)	−2.98 (−13.43, 7.47)	3.79 (−9.80, 17.37)	−4.56 (−17.75, 8.64)	1.69 (−8.08, 11.46)	−3.71 (−13.81, 6.38)	0.26	0.20	0.21

Abbreviations: ANCOVA, analysis of covariance; CI, confidence interval; EMM, estimated marginal mean; EORTC QLQ-C30, European Organisation for Research and Treatment of Cancer Quality of Life Questionnaire Core 30; PUFA, polyunsaturated fatty acid; SFA, saturated fatty acid.

Note: Data for individual phenotypes are expressed as estimated marginal means (95% CIs) derived from Model 2. Annualised changes reflect adjusted score variations from baseline to the 12-month prospective follow-up. Global statistical significance across the 5 phenotypes was determined via type II analysis of covariance (ANCOVA). To

evaluate result robustness, 3 progressive multivariable models were constructed. Model 1 was adjusted for age, sex, cancer type, and the corresponding baseline quality of life score. Model 2 was additionally adjusted for body mass index, monthly personal income, smoking status, drinking status, living arrangement, physical activity, comorbidity burden, duration of survivorship, and disease trajectory. Model 3 was further adjusted for COVID-19 severity. Variations in total analytical sample sizes (*n* = 423, 398, and 395 for Models 1, 2, and 3, respectively) reflect the stepwise restriction to participants with complete covariate data. Inherent item-level missingness was present across specific quality of life domains (maximum missing fraction, 1.2% for the financial difficulties domain).

Table S14. Differences in Annualised Quality-of-Life Change From Baseline to the 12-Month Follow-Up for Specific Phenotypes Compared With the General-Population-Mapped Reference Pool

Variables	Phenotype 2 vs Phenotype 1+4+5	Model 1 *P* value (*N* = 423)	Model 2 *P* value (*N* = 398)	Model 3 *P* value (*N* = 395)	Phenotype 3 vs Phenotype 1+4+5	Model 1 *P* value (*N* = 423)	Model 2 *P* value (*N* = 398)	Model 3 *P* value (*N* = 395)
EORTC QLQ-C30 Summary Score	−4.66 (−8.14, −1.19)	0.02	0.009	0.006	2.45 (−1.06, 5.96)	0.045	0.17	0.15
Global Health Status (QL)	−2.05 (−10.61, 6.51)	0.32	0.64	0.50	5.36 (−3.28, 14.00)	0.046	0.22	0.30
Physical functioning (PF)	−4.67 (−9.41, 0.08)	0.07	0.054	0.07	1.12 (−3.66, 5.91)	0.33	0.64	0.55
Role functioning (RF)	−5.35 (−9.88, −0.83)	0.048	0.02	0.02	1.66 (−2.90, 6.22)	0.29	0.47	0.48
Emotional functioning (EF)	−2.37 (−7.63, 2.89)	0.44	0.38	0.35	4.88 (−0.45, 10.20)	0.07	0.07	0.06
Cognitive functioning (CF)	−1.74 (−8.15, 4.66)	0.43	0.59	0.53	−0.06 (−6.52, 6.40)	0.95	0.99	0.92
Social functioning (SF)	−2.86 (−9.29, 3.57)	0.39	0.38	0.47	4.16 (−2.36, 10.67)	0.11	0.21	0.27
Fatigue (FA)	5.80 (−1.10, 12.71)	0.09	0.10	0.07	−1.84 (−8.81, 5.12)	0.28	0.60	0.66
Nausea and vomiting (NV)	−0.21 (−2.79, 2.36)	0.78	0.87	0.85	−0.16 (−2.76, 2.44)	0.22	0.90	0.67
Pain (PA)	8.28 (2.58, 13.98)	0.008	0.005	0.003	−2.06 (−7.81, 3.70)	0.18	0.48	0.59
Dyspnoea (DY)	6.83 (0.16, 13.50)	0.04	0.045	0.04	−2.92 (−9.63, 3.80)	0.30	0.39	0.21
Insomnia (SL)	4.46 (−4.09, 13.01)	0.42	0.31	0.25	−2.97 (−11.62, 5.69)	0.34	0.50	0.63
Appetite loss (AP)	1.24 (−3.94, 6.42)	0.97	0.64	0.60	1.62 (−3.60, 6.84)	0.34	0.54	0.49
Constipation (CO)	14.10 (7.30, 20.90)	<0.001	<0.001	<0.001	−3.38 (−10.23, 3.46)	0.19	0.33	0.34
Diarrhoea (DI)	0.74 (−4.26, 5.74)	0.98	0.77	0.75	−3.99 (−9.05, 1.06)	0.08	0.12	0.12
Financial difficulties (FI)	5.45 (−4.08, 14.99)	0.35	0.26	0.29	−2.89 (−12.56, 6.78)	0.34	0.56	0.58

Abbreviations: CI, confidence interval; EORTC QLQ-C30, European Organisation for Research and Treatment of Cancer Quality of Life Questionnaire Core 30.

Note: Data are expressed as adjusted annualised mean differences (estimates [95% CIs]) across the 16 EORTC QLQ-C30 domains for specific phenotypes (Phenotypes 2 and 3) compared with the general-population-mapped reference pool (merged Phenotypes 1, 4, and 5). Differences represent between-group contrasts in annualised QoL change from baseline to the 12-month follow-up. Interpretation follows the EORTC QLQ-C30 scoring direction. For functional, global health, and summary scores, positive estimates indicate more favourable change and negative estimates indicate less favourable change relative to the reference pool; for symptom domains, positive estimates

indicate greater symptom worsening and negative estimates indicate relative symptom improvement. Point estimates and 95% CIs are derived exclusively from Model 2. To evaluate result robustness, *P* values are presented across 3 progressive multivariable models. Model 1 was adjusted for age, sex, cancer type, and the corresponding baseline quality of life score. Model 2 was additionally adjusted for body mass index, monthly personal income, smoking status, drinking status, living arrangement, physical activity, comorbidity burden, duration of survivorship, and disease trajectory. Model 3 was further adjusted for COVID-19 severity. Variations in total analytical sample sizes (n = 423, 398, and 395 for Models 1, 2, and 3, respectively) reflect the stepwise restriction to participants with complete covariate data. Inherent item-level missingness was present across specific quality of life domains (maximum missing fraction, 1.2% for the financial difficulties domain).

Table S15. Contextual Interpretation of Differences in Annualised Quality-of-Life Change

Variables	Phenotype 2 vs Phenotype 1+4+5	Relative to 5-point contextual benchmark	Phenotype 3 vs Phenotype 1+4+5	Relative to 5-point contextual benchmark
EORTC QLQ-C30 Summary Score	−4.66 (−8.14, −1.19)	<5	2.45 (−1.06, 5.96)	<5
Global Health Status (QL)	−2.05 (−10.61, 6.51)	<5	5.36 (−3.28, 14.00)	≥5
Physical functioning (PF)	−4.67 (−9.41, 0.08)	<5	1.12 (−3.66, 5.91)	<5
Role functioning (RF)	−5.35 (−9.88, −0.83)	≥5	1.66 (−2.90, 6.22)	<5
Emotional functioning (EF)	−2.37 (−7.63, 2.89)	<5	4.88 (−0.45, 10.20)	<5
Cognitive functioning (CF)	−1.74 (−8.15, 4.66)	<5	−0.06 (−6.52, 6.40)	<5
Social functioning (SF)	−2.86 (−9.29, 3.57)	<5	4.16 (−2.36, 10.67)	<5
Fatigue (FA)	5.80 (−1.10, 12.71)	≥5	−1.84 (−8.81, 5.12)	<5
Nausea and vomiting (NV)	−0.21 (−2.79, 2.36)	<5	−0.16 (−2.76, 2.44)	<5
Pain (PA)	8.28 (2.58, 13.98)	≥5	−2.06 (−7.81, 3.70)	<5
Dyspnoea (DY)	6.83 (0.16, 13.50)	≥5	−2.92 (−9.63, 3.80)	<5
Insomnia (SL)	4.46 (−4.09, 13.01)	<5	−2.97 (−11.62, 5.69)	<5
Appetite loss (AP)	1.24 (−3.94, 6.42)	<5	1.62 (−3.60, 6.84)	<5
Constipation (CO)	14.10 (7.30, 20.90)	≥5	−3.38 (−10.23, 3.46)	<5
Diarrhoea (DI)	0.74 (−4.26, 5.74)	<5	−3.99 (−9.05, 1.06)	<5
Financial difficulties (FI)	5.45 (−4.08, 14.99)	≥5	−2.89 (−12.56, 6.78)	<5

Abbreviations: CI, confidence interval; EORTC QLQ-C30, European Organisation for Research and Treatment of Cancer Quality of Life Questionnaire Core 30; MID, minimally important difference; QoL, quality of life.

Note: Data are presented as Model 2 adjusted annualised mean differences in QoL change (estimate [95% CI]) for Phenotypes 2 and 3 compared with the general-population-mapped reference pool (Phenotypes 1, 4, and 5). Higher functional, global health, and summary scores indicate better QoL or functioning, whereas higher symptom scores indicate greater symptom burden. Classification relative to the 5-point contextual benchmark was based on the absolute magnitude of the point estimate, irrespective of statistical significance. A 5-point absolute difference was used as the contextual benchmark for effect magnitude, representing the lower bound of the

commonly used 5–10-point range for clinically important group-level EORTC QLQ-C30 differences. For the EORTC QLQ-C30 summary score, the 5-point benchmark was used for contextual interpretation because the cited MID guidance did not derive a summary-score-specific MID.

Table S16. Adjusted Annualised Changes in Quality of Life From the 24-Month Retrospective Assessment to the Baseline Across Whole-Blood Fatty Acid Phenotypes

**Variables**	**Phenotype 1 (Moderate SFA, *n* = 104)**	**Phenotype 2 (High-SFA/low-PUFA, *n* = 23)**	**Phenotype 3 (Intermediate SFA-PUFA, *n* = 22)**	**Phenotype 4 (Moderate PUFA, *n* = 119)**	**Phenotype 5 (High PUFA, *n* = 130)**	**Model 1 *P* value (*N* = 423)**	**Model 2 *P* value (*N* = 398)**	**Model 3 *P* value (*N* = 395)**
EORTC QLQ-C30 Summary Score	−1.57 (−3.69, 0.54)	−0.40 (−3.17, 2.37)	−1.97 (−4.65, 0.71)	−1.19 (−3.17, 0.80)	−1.36 (−3.42, 0.70)	0.65	0.75	0.58
Global Health Status (QL)	−5.46 (−10.55, −0.37)	−5.57 (−12.24, 1.10)	−7.73 (−14.15, −1.30)	−4.59 (−9.36, 0.18)	−6.35 (−11.29, −1.40)	0.78	0.64	0.59
Physical functioning (PF)	−0.99 (−3.86, 1.87)	0.44 (−3.31, 4.18)	−1.87 (−5.49, 1.75)	−1.73 (−4.41, 0.95)	−1.65 (−4.44, 1.14)	0.80	0.54	0.54
Role functioning (RF)	−2.35 (−4.97, 0.27)	−0.58 (−4.01, 2.85)	−2.08 (−5.39, 1.23)	−2.33 (−4.78, 0.12)	−1.83 (−4.37, 0.72)	0.62	0.67	0.73
Emotional functioning (EF)	−3.58 (−6.78, −0.39)	−2.63 (−6.80, 1.54)	−5.10 (−9.13, −1.08)	−2.46 (−5.45, 0.54)	−2.56 (−5.66, 0.54)	0.69	0.37	0.36
Cognitive functioning (CF)	−1.94 (−5.94, 2.06)	−2.47 (−7.68, 2.74)	−2.62 (−7.66, 2.42)	−2.85 (−6.59, 0.88)	−2.60 (−6.47, 1.28)	0.98	0.96	0.98
Social functioning (SF)	−3.37 (−7.60, 0.86)	−1.26 (−6.79, 4.28)	−5.70 (−11.07, −0.34)	−3.51 (−7.47, 0.45)	−2.49 (−6.58, 1.61)	0.41	0.43	0.34
Fatigue (FA)	2.37 (−1.72, 6.45)	1.05 (−4.28, 6.38)	2.98 (−2.18, 8.13)	2.65 (−1.17, 6.47)	2.42 (−1.55, 6.39)	0.93	0.95	0.95
Nausea and vomiting (NV)	0.51 (−0.91, 1.93)	0.73 (−1.12, 2.58)	0.08 (−1.70, 1.87)	0.64 (−0.69, 1.96)	0.10 (−1.28, 1.47)	0.85	0.61	0.73
Pain (PA)	2.19 (−1.64, 6.02)	1.53 (−3.48, 6.53)	4.59 (−0.23, 9.42)	2.29 (−1.30, 5.88)	3.22 (−0.49, 6.93)	0.70	0.58	0.47
Dyspnoea (DY)	−2.43 (−6.57, 1.70)	−4.23 (−9.62, 1.17)	−3.16 (−8.38, 2.06)	−2.44 (−6.33, 1.44)	−1.46 (−5.48, 2.56)	0.76	0.65	0.64
Insomnia (SL)	−0.37 (−5.29, 4.55)	−2.25 (−8.69, 4.19)	2.42 (−3.77, 8.61)	−3.49 (−8.11, 1.13)	−1.06 (−5.84, 3.71)	0.13	0.06	0.04
Appetite loss (AP)	3.73 (0.51, 6.95)	1.01 (−3.21, 5.22)	1.98 (−2.05, 6.02)	2.97 (−0.03, 5.97)	3.18 (0.08, 6.29)	0.39	0.46	0.37
Constipation (CO)	4.12 (−0.29, 8.53)	4.46 (−1.33, 10.25)	1.33 (−4.23, 6.89)	2.30 (−1.84, 6.43)	2.85 (−1.44, 7.13)	0.75	0.50	0.46
Diarrhoea (DI)	0.16 (−3.09, 3.40)	−0.85 (−5.09, 3.39)	1.10 (−3.00, 5.20)	−0.74 (−3.79, 2.31)	−0.38 (−3.53, 2.76)	0.49	0.72	0.56
Financial difficulties (FI)	3.14 (−2.50, 8.78)	1.33 (−6.04, 8.69)	6.66 (−0.48, 13.80)	2.27 (−3.01, 7.55)	1.00 (−4.46, 6.45)	0.25	0.28	0.29

Abbreviations: ANCOVA, analysis of covariance; CI, confidence interval; EMM, estimated marginal mean; EORTC QLQ-C30, European Organisation for Research and Treatment of Cancer Quality of Life Questionnaire Core 30; PUFA, polyunsaturated fatty acid; SFA, saturated fatty acid.

Note: Data for individual phenotypes are expressed as estimated marginal means (95% CIs) derived from Model 2. Annualised changes reflect adjusted score variations from the 24-month retrospective assessment to the baseline. Global statistical significance across the 5 phenotypes was determined via type II analysis of covariance (ANCOVA). To evaluate result robustness, 3 progressive multivariable models were constructed. Model 1 was adjusted for age, sex, cancer type, and the corresponding 24-month retrospective quality of life score. Model 2 was additionally adjusted for body mass index, monthly personal income, smoking status, drinking status, living arrangement, physical activity, comorbidity burden, duration of survivorship, and disease trajectory. Model 3 was further adjusted for COVID-19 severity. Variations in total analytical sample sizes (*n* = 423, 398, and 395 for Models 1, 2, and 3, respectively) reflect the stepwise restriction to participants with complete covariate data. Inherent item-level missingness was present across specific quality of life domains (maximum missing fraction, 1.2% for the financial difficulties domain).

Table S17. Differences in Annualised Quality-of-Life Change From the 24-Month Retrospective Assessment to Baseline for Specific Phenotypes Compared With the General-Population-Mapped Reference Pool

**Variables**	**Phenotype 2 vs Phenotype 1+4+5**	**Model 1 *P* value (*N* = 423)**	**Model 2 *P* value (*N* = 398)**	**Model 3 *P* value (*N* = 395)**	**Phenotype 3 vs Phenotype 1+4+5**	**Model 1 *P* value (*N* = 423)**	**Model 2 *P* value (*N* = 398)**	**Model 3 *P* value (*N* = 395)**
EORTC QLQ-C30 Summary Score	0.98 (−0.96, 2.92)	0.42	0.32	0.25	−0.60 (−2.55, 1.36)	0.55	0.55	0.46
Global Health Status (QL)	−0.10 (−4.77, 4.56)	0.83	0.96	0.87	−2.26 (−6.96, 2.44)	0.60	0.34	0.40
Physical functioning (PF)	1.89 (−0.73, 4.52)	0.24	0.16	0.20	−0.41 (−3.07, 2.24)	0.67	0.76	0.61
Role functioning (RF)	1.59 (−0.82, 4.00)	0.38	0.20	0.25	0.09 (−2.34, 2.51)	0.71	0.94	0.96
Emotional functioning (EF)	0.24 (−2.69, 3.17)	0.56	0.87	0.89	−2.24 (−5.19, 0.71)	0.60	0.14	0.13
Cognitive functioning (CF)	−0.01 (−3.65, 3.63)	0.94	>0.99	0.88	−0.16 (−3.83, 3.52)	0.77	0.93	0.99
Social functioning (SF)	1.87 (−2.00, 5.74)	0.24	0.34	0.33	−2.58 (−6.50, 1.34)	0.61	0.20	0.13
Fatigue (FA)	−1.43 (−5.17, 2.31)	0.66	0.45	0.42	0.50 (−3.28, 4.28)	0.58	0.80	0.82
Nausea and vomiting (NV)	0.32 (−0.99, 1.62)	0.70	0.63	0.97	−0.33 (−1.64, 0.98)	0.91	0.62	0.58
Pain (PA)	−1.04 (−4.55, 2.48)	0.98	0.56	0.38	2.02 (−1.52, 5.57)	0.46	0.26	0.28
Dyspnoea (DY)	−2.12 (−5.91, 1.68)	0.77	0.27	0.24	−1.05 (−4.88, 2.78)	0.73	0.59	0.84
Insomnia (SL)	−0.61 (−5.13, 3.91)	0.64	0.79	0.56	4.06 (−0.48, 8.61)	0.14	0.08	0.10
Appetite loss (AP)	−2.29 (−5.24, 0.67)	0.15	0.13	0.11	−1.31 (−4.27, 1.65)	0.55	0.38	0.35
Constipation (CO)	1.38 (−2.67, 5.42)	0.60	0.50	0.63	−1.76 (−5.82, 2.30)	0.79	0.39	0.44
Diarrhoea (DI)	−0.53 (−3.50, 2.43)	0.92	0.72	0.68	1.42 (−1.57, 4.42)	0.12	0.35	0.23
Financial difficulties (FI)	−0.81 (−5.97, 4.36)	0.76	0.76	0.78	4.52 (−0.72, 9.76)	0.23	0.09	0.10

Abbreviations: CI, confidence interval; EORTC QLQ-C30, European Organisation for Research and Treatment of Cancer Quality of Life Questionnaire Core 30.

Note: Data are expressed as adjusted annualised mean differences (estimates [95% CIs]) across the 16 EORTC QLQ-C30 domains for specific phenotypes (Phenotypes 2 and 3) compared with the general-population-mapped reference pool (merged Phenotypes 1, 4, and 5). Differences represent between-group contrasts in annualised QoL change from the 24-month retrospective assessment to baseline. Interpretation follows the EORTC QLQ-C30 scoring direction. For functional, global health, and summary scores, positive estimates indicate more favourable change and negative estimates indicate less favourable change relative to the reference pool; for symptom domains, positive

estimates indicate greater symptom worsening and negative estimates indicate relative symptom improvement. Point estimates and 95% CIs are derived exclusively from Model 2. To evaluate result robustness, *P* values are presented across 3 progressive multivariable models. Model 1 was adjusted for age, sex, cancer type, and the corresponding 24-month retrospective quality of life score. Model 2 was additionally adjusted for body mass index, monthly personal income, smoking status, drinking status, living arrangement, physical activity, comorbidity burden, duration of survivorship, and disease trajectory. Model 3 was further adjusted for COVID-19 severity. Variations in total analytical sample sizes (*n* = 423, 398, and 395 for Models 1, 2, and 3, respectively) reflect the stepwise restriction to participants with complete covariate data. Inherent item-level missingness was present across specific quality of life domains (maximum missing fraction, 1.2% for the financial difficulties domain).

Table S18. Adjusted Annualised Changes in Quality of Life From Baseline to the 12-Month Follow-Up Across Whole-Blood Fatty Acid Phenotypes Using Multiple Imputation

**Variables**	**Phenotype 1 (Moderate SFA, *n* = 113)**	**Phenotype 2 (High-SFA/low-PUFA, *n* = 24)**	**Phenotype 3 (Intermediate SFA-PUFA, *n* = 24)**	**Phenotype 4 (Moderate PUFA, *n* = 128)**	**Phenotype 5 (High PUFA, *n* = 134)**	**Model 1 *P* value (*N* = 423)**	**Model 2 *P* value (*N* = 423)**	**Model 3 *P* value (*N* = 423)**
EORTC QLQ-C30 Summary Score	−0.48 (−4.26, 3.30)	−4.94 (−9.87, −0.01)	2.18 (−2.50, 6.85)	−0.40 (−3.93, 3.13)	−0.01 (−3.71, 3.70)	0.03	0.04	0.03
Global Health Status (QL)	−13.29 (−22.35, −4.23)	−16.85 (−28.72, −4.97)	−8.64 (−19.86, 2.58)	−15.43 (−23.91, −6.95)	−13.31 (−22.21, −4.40)	0.17	0.46	0.49
Physical functioning (PF)	1.03 (−4.14, 6.19)	−2.59 (−9.34, 4.16)	2.86 (−3.51, 9.23)	3.04 (−1.80, 7.88)	2.27 (−2.79, 7.34)	0.21	0.19	0.21
Role functioning (RF)	−1.59 (−6.61, 3.43)	−7.11 (−13.65, −0.56)	−0.51 (−6.71, 5.69)	−1.90 (−6.59, 2.80)	−2.26 (−7.16, 2.65)	0.23	0.18	0.20
Emotional functioning (EF)	1.74 (−3.90, 7.37)	−1.53 (−8.87, 5.81)	5.10 (−1.84, 12.05)	0.68 (−4.57, 5.94)	0.71 (−4.80, 6.22)	0.30	0.32	0.28
Cognitive functioning (CF)	0.74 (−6.14, 7.63)	−0.62 (−9.62, 8.38)	−0.31 (−8.80, 8.18)	2.65 (−3.80, 9.09)	2.61 (−4.14, 9.36)	0.64	0.64	0.59
Social functioning (SF)	−2.71 (−9.61, 4.19)	−4.21 (−13.21, 4.79)	1.93 (−6.61, 10.47)	−2.91 (−9.37, 3.55)	1.37 (−5.36, 8.11)	0.02	0.07	0.09
Fatigue (FA)	2.00 (−5.45, 9.45)	8.12 (−1.61, 17.85)	−0.29 (−9.50, 8.92)	1.30 (−5.67, 8.27)	2.31 (−4.98, 9.61)	0.36	0.35	0.30
Nausea and vomiting (NV)	1.59 (−1.39, 4.57)	0.81 (−3.09, 4.71)	0.56 (−3.12, 4.25)	1.24 (−1.55, 4.03)	1.22 (−1.69, 4.14)	0.76	0.95	0.88
Pain (PA)	−0.59 (−6.70, 5.52)	7.03 (−0.96, 15.02)	−4.58 (−12.13, 2.97)	−1.65 (−7.37, 4.06)	−2.22 (−8.21, 3.78)	0.04	0.02	0.01
Dyspnoea (DY)	0.17 (−7.03, 7.37)	6.88 (−2.54, 16.29)	−2.98 (−11.85, 5.89)	−0.58 (−7.32, 6.16)	−1.39 (−8.43, 5.66)	0.21	0.17	0.12
Insomnia (SL)	−0.20 (−9.27, 8.87)	4.05 (−7.83, 15.92)	−4.23 (−15.42, 6.96)	−0.44 (−8.95, 8.08)	0.45 (−8.42, 9.32)	0.70	0.66	0.66
Appetite loss (AP)	3.98 (−1.67, 9.63)	4.92 (−2.40, 12.25)	4.52 (−2.40, 11.44)	3.59 (−1.67, 8.84)	5.38 (−0.12, 10.89)	0.76	0.79	0.81
Constipation (CO)	3.37 (−4.04, 10.77)	18.20 (8.52, 27.88)	1.09 (−7.99, 10.18)	6.26 (−0.64, 13.16)	3.83 (−3.40, 11.06)	<0.001	<0.001	<0.001
Diarrhoea (DI)	1.55 (−3.82, 6.92)	1.90 (−5.12, 8.92)	−3.03 (−9.67, 3.61)	1.39 (−3.63, 6.41)	1.66 (−3.59, 6.91)	0.54	0.45	0.38
Financial difficulties (FI)	−3.17 (−13.64, 7.29)	1.95 (−11.68, 15.59)	−3.67 (−16.61, 9.27)	0.14 (−9.63, 9.91)	−4.75 (−14.96, 5.46)	0.26	0.41	0.42

Abbreviations: ANCOVA, analysis of covariance; CI, confidence interval; EMM, estimated marginal mean; EORTC QLQ-C30, European Organisation for Research and Treatment of Cancer Quality of Life Questionnaire Core 30; MICE, multiple imputation by chained equations; PUFA, polyunsaturated fatty acid; SFA, saturated fatty acid.

Note: Data for individual phenotypes are expressed as pooled estimated marginal means (95% CIs) derived from Model 2 across 20 multiply imputed datasets. Annualised changes reflect adjusted score variations from baseline to the 12-month prospective follow-up. Missing covariate and outcome data were addressed using multiple imputation by chained equations (MICE) to preserve statistical power and mitigate potential bias. Global statistical significance across the 5 phenotypes was determined by pooling *F* statistics (D1 statistic) from type II analysis of covariance (ANCOVA). Pairwise comparisons were synthesized using Rubin rules to incorporate both within-imputation and between-imputation variance. To evaluate result robustness, 3 progressive multivariable models were constructed. Model 1 was adjusted for age, sex, cancer type, and the corresponding baseline quality of life score. Model 2 was additionally adjusted for body mass index, educational level, monthly personal income, smoking status, drinking status, living arrangement, physical activity, comorbidity burden, duration of survivorship, and disease trajectory. Model 3 was further adjusted for COVID-19 severity. Notably, educational level—which was excluded from the primary complete-case analysis due to a high missing data rate—was incorporated into the imputed models to maximize confounding adjustment without compromising sample size. In contrast to the complete-case analysis, the total analytical sample size remained consistent across all models ($N = 423$), as missing values were successfully accounted for through the imputation process.

Table S19. Differences in Annualised Quality-of-Life Change From Baseline to the 12-Month Follow-Up for Specific Phenotypes Compared With the General-Population-Mapped Reference Pool Using Multiple Imputation

**Variables**	**Phenotype 2 vs Phenotype 1+4+5**	**Model 1 *P* value (*N* = 423)**	**Model 2 *P* value (*N* = 423)**	**Model 3 *P* value (*N* = 423)**	**Phenotype 3 vs Phenotype 1+4+5**	**Model 1 *P* value (*N* = 423)**	**Model 2 *P* value (*N* = 423)**	**Model 3 *P* value (*N* = 423)**
EORTC QLQ-C30 Summary Score	−4.64 (−8.06, −1.22)	0.02	0.008	0.005	2.47 (−0.91, 5.85)	0.045	0.15	0.17
Global Health Status (QL)	−2.84 (−11.05, 5.37)	0.32	0.50	0.41	5.37 (−2.72, 13.46)	0.045	0.19	0.31
Physical functioning (PF)	−4.70 (−9.39, −0.02)	0.07	0.049	0.06	0.74 (−3.87, 5.35)	0.33	0.75	0.74
Role functioning (RF)	−5.19 (−9.73, −0.65)	0.047	0.02	0.02	1.41 (−3.06, 5.87)	0.29	0.54	0.61
Emotional functioning (EF)	−2.57 (−7.65, 2.50)	0.44	0.32	0.28	4.06 (−0.95, 9.07)	0.06	0.11	0.11
Cognitive functioning (CF)	−2.62 (−8.85, 3.61)	0.43	0.41	0.32	−2.31 (−8.45, 3.83)	0.95	0.46	0.42
Social functioning (SF)	−2.79 (−9.04, 3.45)	0.39	0.38	0.46	3.34 (−2.82, 9.50)	0.11	0.29	0.39
Fatigue (FA)	6.25 (−0.50, 13.00)	0.09	0.07	0.04	−2.16 (−8.83, 4.50)	0.28	0.52	0.65
Nausea and vomiting (NV)	−0.54 (−3.24, 2.16)	0.78	0.69	0.54	−0.79 (−3.45, 1.87)	0.22	0.56	0.47
Pain (PA)	8.52 (2.97, 14.06)	0.008	0.003	0.002	−3.10 (−8.56, 2.37)	0.18	0.27	0.30
Dyspnoea (DY)	7.47 (0.95, 14.00)	0.04	0.02	0.02	−2.38 (−8.80, 4.04)	0.30	0.47	0.36
Insomnia (SL)	4.11 (−4.12, 12.34)	0.42	0.33	0.24	−4.17 (−12.29, 3.96)	0.34	0.32	0.42
Appetite loss (AP)	0.61 (−4.47, 5.68)	0.97	0.82	0.75	0.20 (−4.80, 5.21)	0.34	0.94	0.78
Constipation (CO)	13.71 (7.02, 20.41)	< 0.001	< 0.001	< 0.001	−3.39 (−9.96, 3.17)	0.19	0.31	0.33
Diarrhoea (DI)	0.37 (−4.49, 5.22)	0.98	0.88	0.80	−4.56 (−9.35, 0.23)	0.08	0.06	0.049
Financial difficulties (FI)	4.55 (−4.91, 14.01)	0.35	0.35	0.38	−1.07 (−10.42, 8.27)	0.34	0.82	0.94

Abbreviations: CI, confidence interval; EORTC QLQ-C30, European Organisation for Research and Treatment of Cancer Quality of Life Questionnaire Core 30; MICE, multiple imputation by chained equations.

Note: Data are expressed as pooled adjusted annualised mean differences (estimates [95% CIs]) across the 16 EORTC QLQ-C30 domains for specific phenotypes (Phenotypes 2 and 3) compared with the general-population-mapped reference pool (merged Phenotypes 1, 4, and 5). Differences represent between-group contrasts in annualised QoL change from baseline to the 12-month follow-up. Interpretation follows the EORTC QLQ-C30 scoring direction. For functional, global health, and summary

scores, positive estimates indicate more favourable change and negative estimates indicate less favourable change relative to the reference pool; for symptom domains, positive estimates indicate greater symptom worsening and negative estimates indicate relative symptom improvement. Missing covariate and outcome data were addressed using multiple imputation by chained equations (MICE) with 20 imputed datasets. Point estimates and 95% CIs derived exclusively from Model 2 were synthesized using Rubin rules to incorporate within-imputation and between-imputation variance. To evaluate result robustness, *P* values are presented across 3 progressive multivariable models. Model 1 was adjusted for age, sex, cancer type, and the corresponding baseline quality of life score. Model 2 was additionally adjusted for body mass index, educational level, monthly personal income, smoking status, drinking status, living arrangement, physical activity, comorbidity burden, duration of survivorship, and disease trajectory. Model 3 was further adjusted for COVID-19 severity. Notably, educational level—which was excluded from the primary complete-case analysis due to a high missing data rate—was incorporated into the imputed models to maximize confounding adjustment without compromising sample size. In contrast to the complete-case analysis, the total analytical sample size remained consistent across all models (N = 423), as missing values were successfully accounted for through the imputation process.

Table S20. Adjusted Annualised Changes in Quality of Life From the 24-Month Retrospective Assessment to the Baseline Across Whole-Blood Fatty Acid Phenotypes Using Multiple Imputation

Variables	Phenotype 1 (Moderate SFA, *n* = 113)	Phenotype 2 (High-SFA/low-PUFA, *n* = 24)	Phenotype 3 (Intermediate SFA-PUFA, *n* = 24)	Phenotype 4 (Moderate PUFA, *n* = 128)	Phenotype 5 (High PUFA, *n* = 134)	Model 1 *P* value (*N* = 423)	Model 2 *P* value (*N* = 423)	Model 3 *P* value (*N* = 423)
EORTC QLQ-C30 Summary Score	−2.00 (−4.11, 0.10)	−0.81 (−3.57, 1.94)	−2.53 (−5.14, 0.07)	−1.35 (−3.32, 0.62)	−1.67 (−3.74, 0.39)	0.65	0.54	0.40
Global Health Status (QL)	−5.28 (−10.31, −0.24)	−4.96 (−11.57, 1.65)	−7.10 (−13.34, −0.87)	−4.08 (−8.80, 0.64)	−6.06 (−11.00, −1.12)	0.78	0.55	0.52
Physical functioning (PF)	−1.50 (−4.33, 1.33)	0.03 (−3.68, 3.74)	−2.35 (−5.85, 1.16)	−1.86 (−4.51, 0.79)	−1.92 (−4.70, 0.85)	0.80	0.67	0.72
Role functioning (RF)	−2.89 (−5.60, −0.18)	−1.13 (−4.69, 2.42)	−3.55 (−6.89, −0.20)	−2.47 (−5.01, 0.06)	−2.14 (−4.79, 0.52)	0.62	0.56	0.55
Emotional functioning (EF)	−3.65 (−6.80, −0.49)	−2.32 (−6.44, 1.80)	−4.24 (−8.13, −0.34)	−2.30 (−5.25, 0.65)	−2.60 (−5.68, 0.49)	0.69	0.45	0.39
Cognitive functioning (CF)	−2.41 (−6.32, 1.50)	−2.41 (−7.51, 2.68)	−2.65 (−7.47, 2.17)	−2.79 (−6.44, 0.86)	−2.87 (−6.69, 0.96)	0.98	0.99	0.98
Social functioning (SF)	−3.60 (−7.80, 0.60)	−0.96 (−6.46, 4.54)	−5.16 (−10.36, 0.05)	−3.07 (−7.00, 0.86)	−2.23 (−6.34, 1.88)	0.41	0.39	0.32
Fatigue (FA)	3.12 (−0.97, 7.20)	1.83 (−3.52, 7.17)	4.40 (−0.64, 9.45)	3.27 (−0.55, 7.09)	2.99 (−1.01, 7.00)	0.93	0.91	0.90
Nausea and vomiting (NV)	0.59 (−0.86, 2.04)	0.78 (−1.11, 2.68)	0.89 (−0.90, 2.67)	0.75 (−0.60, 2.11)	0.24 (−1.18, 1.66)	0.85	0.72	0.81
Pain (PA)	2.04 (−1.76, 5.84)	1.93 (−3.04, 6.90)	4.00 (−0.68, 8.68)	1.69 (−1.86, 5.25)	3.24 (−0.47, 6.95)	0.70	0.45	0.32
Dyspnoea (DY)	−1.18 (−5.26, 2.90)	−2.88 (−8.21, 2.44)	−2.38 (−7.42, 2.67)	−1.88 (−5.70, 1.94)	−0.71 (−4.70, 3.28)	0.76	0.66	0.71
Insomnia (SL)	1.06 (−3.83, 5.95)	−0.92 (−7.31, 5.48)	3.94 (−2.08, 9.97)	−1.67 (−6.25, 2.92)	0.71 (−4.06, 5.48)	0.13	0.08	0.04
Appetite loss (AP)	4.56 (1.34, 7.78)	1.66 (−2.56, 5.88)	3.03 (−0.92, 6.98)	3.25 (0.25, 6.25)	3.70 (0.56, 6.84)	0.39	0.32	0.26
Constipation (CO)	3.58 (−0.75, 7.91)	4.09 (−1.60, 9.78)	1.94 (−3.41, 7.29)	1.89 (−2.16, 5.94)	2.21 (−2.03, 6.45)	0.75	0.54	0.57
Diarrhoea (DI)	0.05 (−3.25, 3.34)	−0.38 (−4.67, 3.91)	1.58 (−2.47, 5.63)	−0.80 (−3.90, 2.29)	−0.45 (−3.67, 2.78)	0.49	0.56	0.47
Financial difficulties (FI)	3.52 (−2.00, 9.03)	1.06 (−6.15, 8.28)	6.24 (−0.60, 13.08)	2.16 (−3.00, 7.32)	1.15 (−4.25, 6.54)	0.25	0.28	0.29

Abbreviations: ANCOVA, analysis of covariance; CI, confidence interval; EMM, estimated marginal mean; EORTC QLQ-C30, European Organisation for Research and Treatment of Cancer Quality of Life Questionnaire Core 30; MICE, multiple imputation by chained equations; PUFA, polyunsaturated fatty acid; SFA, saturated fatty acid.

Note: Data for individual phenotypes are expressed as pooled estimated marginal means (95% CIs) derived from Model 2 across 20 multiply imputed datasets. Annualised changes reflect adjusted score variations from the 24-month retrospective assessment to the baseline. Missing covariate and outcome data were addressed using multiple imputation by chained equations (MICE) to preserve statistical power and mitigate potential bias. Global statistical significance across the 5 phenotypes was determined by pooling *F* statistics (D1 statistic) from type II analysis of covariance (ANCOVA). Pairwise comparisons were synthesized using Rubin rules to incorporate both within-imputation and between-imputation variance. To evaluate result robustness, 3 progressive multivariable models were constructed. Model 1 was adjusted for age, sex, cancer type, and the corresponding 24-month retrospective quality of life score. Model 2 was additionally adjusted for body mass index, educational level, monthly personal income, smoking status, drinking status, living arrangement, physical activity, comorbidity burden, duration of survivorship, and disease trajectory. Model 3 was further adjusted for COVID-19 severity. Notably, educational level—which was excluded from the primary complete-case analysis due to a high missing data rate—was incorporated into the imputed models to maximize confounding adjustment without compromising sample size. In contrast to the complete-case analysis, the total analytical sample size remained consistent across all models ($N = 423$), as missing values were successfully accounted for through the imputation process.

Table S21. Differences in Annualised Quality-of-Life Change From the 24-Month Retrospective Assessment to Baseline for Specific Phenotypes Compared With the General-Population-Mapped Reference Pool Using Multiple Imputation

Variables	Phenotype 2 vs Phenotype 1+4+5	Model 1 *P* value (*N* = 423)	Model 2 *P* value (*N* = 423)	Model 3 *P* value (*N* = 423)	Phenotype 3 vs Phenotype 1+4+5	Model 1 *P* value (*N* = 423)	Model 2 *P* value (*N* = 423)	Model 3 *P* value (*N* = 423)
EORTC QLQ-C30 Summary Score	0.86 (−1.05, 2.77)	0.42	0.38	0.35	−0.86 (−2.74, 1.02)	0.55	0.37	0.30
Global Health Status (QL)	0.18 (−4.39, 4.75)	0.83	0.94	0.83	−1.97 (−6.46, 2.53)	0.60	0.39	0.43
Physical functioning (PF)	1.79 (−0.78, 4.36)	0.24	0.17	0.24	−0.59 (−3.12, 1.95)	0.67	0.65	0.51
Role functioning (RF)	1.37 (−1.10, 3.83)	0.38	0.28	0.39	−1.05 (−3.46, 1.37)	0.71	0.40	0.28
Emotional functioning (EF)	0.53 (−2.32, 3.38)	0.56	0.71	0.82	−1.39 (−4.19, 1.42)	0.60	0.33	0.28
Cognitive functioning (CF)	0.27 (−3.24, 3.79)	0.94	0.88	0.78	0.04 (−3.42, 3.50)	0.77	0.98	0.90
Social functioning (SF)	2.00 (−1.80, 5.81)	0.24	0.30	0.33	−2.19 (−5.94, 1.56)	0.61	0.25	0.17
Fatigue (FA)	−1.30 (−5.00, 2.40)	0.66	0.49	0.50	1.28 (−2.37, 4.92)	0.58	0.49	0.50
Nausea and vomiting (NV)	0.25 (−1.06, 1.57)	0.70	0.70	0.98	0.36 (−0.93, 1.65)	0.90	0.58	0.50
Pain (PA)	−0.39 (−3.84, 3.05)	0.98	0.82	0.67	1.67 (−1.71, 5.06)	0.46	0.33	0.29
Dyspnoea (DY)	−1.63 (−5.32, 2.06)	0.77	0.39	0.38	−1.12 (−4.75, 2.51)	0.73	0.55	0.75
Insomnia (SL)	−0.95 (−5.38, 3.48)	0.64	0.67	0.52	3.91 (−0.44, 8.27)	0.14	0.08	0.09
Appetite loss (AP)	−2.17 (−5.10, 0.75)	0.15	0.14	0.14	−0.81 (−3.67, 2.05)	0.55	0.58	0.60
Constipation (CO)	1.53 (−2.40, 5.46)	0.60	0.44	0.59	−0.62 (−4.47, 3.22)	0.79	0.75	0.71
Diarrhoea (DI)	0.02 (−2.91, 2.95)	0.92	0.99	0.98	1.98 (−0.90, 4.86)	0.12	0.18	0.15
Financial difficulties (FI)	−1.21 (−6.21, 3.79)	0.76	0.64	0.70	3.96 (−0.97, 8.90)	0.23	0.12	0.12

Abbreviations: CI, confidence interval; EORTC QLQ-C30, European Organisation for Research and Treatment of Cancer Quality of Life Questionnaire Core 30; MICE, multiple imputation by chained equations.

Note: Data are expressed as pooled adjusted annualised mean differences (estimates [95% CIs]) across the 16 EORTC QLQ-C30 domains for specific phenotypes (Phenotypes 2 and 3) compared with the general-population-mapped reference pool (merged Phenotypes 1, 4, and 5). Differences represent between-group contrasts in annualised QoL change from the 24-month retrospective assessment to baseline. Interpretation follows the EORTC QLQ-C30 scoring direction. For functional, global health,

and summary scores, positive estimates indicate more favourable change and negative estimates indicate less favourable change relative to the reference pool; for symptom domains, positive estimates indicate greater symptom worsening and negative estimates indicate relative symptom improvement. Missing covariate and outcome data were addressed using multiple imputation by chained equations (MICE) with 20 imputed datasets. Point estimates and 95% CIs derived exclusively from Model 2 were synthesized using Rubin rules to incorporate within-imputation and between-imputation variance. To evaluate result robustness, *P* values are presented across 3 progressive multivariable models. Model 1 was adjusted for age, sex, cancer type, and the corresponding 24-month retrospective quality of life score. Model 2 was additionally adjusted for body mass index, educational level, monthly personal income, smoking status, drinking status, living arrangement, physical activity, comorbidity burden, duration of survivorship, and disease trajectory. Model 3 was further adjusted for COVID-19 severity. Notably, educational level—which was excluded from the primary complete-case analysis due to a high missing data rate—was incorporated into the imputed models to maximize confounding adjustment without compromising sample size. In contrast to the complete-case analysis, the total analytical sample size remained consistent across all models ($N = 423$), as missing values were successfully accounted for through the imputation process.

Table S22. Dietary-Adjusted Sensitivity Analyses of Differences in Annualised Quality-of-Life Change From Baseline to the 12-Month Follow-Up for Phenotypes 2 and 3 Compared With the General-Population-Mapped Reference Pool

**Variables**	**Phenotype 2 Restricted Model 2 (*N* = 374)**	***P* value**	**Phenotype 2 Diet-adjusted model (*N* = 374)**	***P* value**	**Phenotype 3 Restricted Model 2 (*N* = 374)**	***P* value**	**Phenotype 3 Diet-adjusted model (*N* = 374)**	***P* value**
EORTC QLQ-C30 Summary Score	−4.70 (−8.26, −1.14)	0.01	−4.93 (−8.52, −1.35)	0.007	2.86 (−0.76, 6.47)	0.12	2.83 (−0.79, 6.45)	0.12
Global Health Status (QL)	−2.67 (−11.28, 5.95)	0.54	−3.13 (−11.81, 5.55)	0.48	6.54 (−2.20, 15.28)	0.14	6.47 (−2.28, 15.22)	0.15
Physical functioning (PF)	−4.58 (−9.48, 0.32)	0.07	−4.90 (−9.82, 0.02)	0.051	0.82 (−4.16, 5.80)	0.75	0.83 (−4.15, 5.80)	0.74
Role functioning (RF)	−5.65 (−10.21, −1.09)	0.02	−6.13 (−10.70, −1.56)	0.009	1.75 (−2.87, 6.37)	0.46	1.70 (−2.90, 6.30)	0.47
Emotional functioning (EF)	−2.40 (−7.84, 3.04)	0.39	−2.64 (−8.13, 2.84)	0.34	5.91 (0.39, 11.44)	0.04	5.89 (0.35, 11.43)	0.04
Cognitive functioning (CF)	−2.21 (−8.49, 4.07)	0.49	−2.53 (−8.86, 3.79)	0.43	−0.07 (−6.43, 6.29)	0.98	−0.12 (−6.49, 6.25)	0.97
Social functioning (SF)	−2.86 (−9.40, 3.69)	0.39	−3.06 (−9.66, 3.53)	0.36	4.67 (−2.01, 11.34)	0.17	4.60 (−2.09, 11.29)	0.18
Fatigue (FA)	6.44 (−0.60, 13.48)	0.07	6.96 (−0.12, 14.04)	0.054	−2.07 (−9.21, 5.06)	0.57	−2.02 (−9.15, 5.12)	0.58
Nausea and vomiting (NV)	−0.14 (−2.60, 2.33)	0.91	0.09 (−2.38, 2.57)	0.94	−0.44 (−2.93, 2.05)	0.73	−0.42 (−2.91, 2.06)	0.74
Pain (PA)	8.58 (2.73, 14.43)	0.004	8.86 (2.97, 14.76)	0.003	−1.52 (−7.45, 4.42)	0.62	−1.48 (−7.42, 4.47)	0.63
Dyspnoea (DY)	7.19 (0.31, 14.07)	0.04	7.07 (0.14, 14.00)	0.046	−4.23 (−11.18, 2.73)	0.23	−4.21 (−11.19, 2.76)	0.24
Insomnia (SL)	4.75 (−3.99, 13.50)	0.29	5.09 (−3.71, 13.90)	0.26	−3.37 (−12.27, 5.53)	0.46	−3.40 (−12.31, 5.52)	0.45
Appetite loss (AP)	1.74 (−3.58, 7.06)	0.52	1.93 (−3.42, 7.27)	0.48	1.48 (−3.90, 6.86)	0.59	1.53 (−3.85, 6.92)	0.58
Constipation (CO)	12.99 (5.91, 20.07)	<0.001	13.26 (6.13, 20.40)	<0.001	−3.72 (−10.87, 3.43)	0.31	−3.67 (−10.83, 3.49)	0.31
Diarrhoea (DI)	0.72 (−4.29, 5.73)	0.78	0.68 (−4.37, 5.73)	0.79	−4.45 (−9.54, 0.63)	0.09	−4.48 (−9.58, 0.61)	0.08
Financial difficulties (FI)	6.15 (−3.57, 15.87)	0.21	6.00 (−3.80, 15.81)	0.23	−3.21 (−13.12, 6.69)	0.52	−3.25 (−13.19, 6.69)	0.52

Abbreviations: CI, confidence interval; EORTC QLQ-C30, European Organisation for Research and Treatment of Cancer Quality of Life Questionnaire Core 30; QoL, quality of life.

Note: Data are expressed as adjusted annualised mean differences (estimates [95% CIs]) across the 16 EORTC QLQ-C30 domains for Phenotypes 2 and 3 compared with the general-population-mapped reference pool (Phenotypes 1, 4, and 5). Differences represent between-group contrasts in annualised QoL change from baseline to the 12-month follow-up. Interpretation follows the EORTC QLQ-C30 scoring direction. For functional, global health, and summary scores, positive estimates indicate more favourable

change and negative estimates indicate less favourable change relative to the reference pool; for symptom domains, positive estimates indicate greater symptom worsening and negative estimates indicate relative symptom improvement. The restricted Model 2 was fitted in the same participants eligible for the dietary sensitivity analysis. The diet-adjusted Model 2 used the identical analytical sample and additionally adjusted for total energy intake and intake of beef, mutton, and other red meats. Both models otherwise adjusted for age, sex, cancer type, body mass index, monthly personal income, smoking status, drinking status, living arrangement, physical activity, comorbidity burden, duration of survivorship, disease trajectory, and the corresponding baseline QoL score. Small variations in analytical sample size across QoL domains reflect item-level missingness.

Table S23. Restricted-Sample Sensitivity Analyses of Differences in Annualised Quality-of-Life Change From Baseline to the 12-Month Follow-Up for Phenotype 2 Compared With the General-Population-Mapped Reference Pool

**Variables**	**Phenotype 2 Excluding breast cancer (*N* = 283)**	***P* value**	**Phenotype 2 Women only (*N* = 296)**	***P* value**	**Phenotype 2 Survivorship 5–10 years (*N* = 236)**	***P* value**
EORTC QLQ-C30 Summary Score	−5.52 (−9.53, −1.51)	0.007	−4.01 (−8.02, 0.00)	0.050	−6.69 (−10.98, −2.41)	0.002
Global Health Status (QL)	−0.45 (−10.50, 9.60)	0.93	−3.00 (−13.02, 7.03)	0.56	0.96 (−9.71, 11.63)	0.86
Physical functioning (PF)	−5.42 (−10.62, −0.23)	0.04	−3.74 (−9.09, 1.61)	0.17	−7.27 (−13.28, −1.26)	0.02
Role functioning (RF)	−4.26 (−9.25, 0.72)	0.09	−5.62 (−10.59, −0.64)	0.03	−7.00 (−12.66, −1.34)	0.02
Emotional functioning (EF)	−5.83 (−11.01, −0.64)	0.03	−0.73 (−6.93, 5.47)	0.82	−4.27 (−10.86, 2.31)	0.20
Cognitive functioning (CF)	−4.93 (−12.11, 2.25)	0.18	−1.33 (−8.93, 6.27)	0.73	−4.39 (−12.37, 3.60)	0.28
Social functioning (SF)	−2.83 (−9.91, 4.24)	0.43	−2.78 (−10.00, 4.44)	0.45	−4.52 (−12.87, 3.83)	0.29
Fatigue (FA)	6.25 (−1.45, 13.96)	0.11	6.04 (−1.96, 14.04)	0.14	9.98 (1.66, 18.30)	0.02
Nausea and vomiting (NV)	−0.12 (−3.41, 3.16)	0.94	−2.14 (−5.12, 0.84)	0.16	0.02 (−3.61, 3.64)	0.99
Pain (PA)	7.74 (1.40, 14.09)	0.02	7.93 (1.38, 14.48)	0.02	11.99 (4.69, 19.30)	0.001
Dyspnoea (DY)	8.94 (1.38, 16.50)	0.02	6.91 (−0.61, 14.44)	0.07	8.07 (−0.49, 16.64)	0.06
Insomnia (SL)	4.50 (−5.29, 14.28)	0.37	4.67 (−5.52, 14.86)	0.37	7.96 (−3.06, 18.97)	0.16
Appetite loss (AP)	1.88 (−3.79, 7.54)	0.52	0.12 (−6.15, 6.38)	0.97	4.08 (−2.46, 10.61)	0.22
Constipation (CO)	17.72 (10.15, 25.29)	<0.001	13.57 (5.37, 21.78)	0.001	17.02 (7.98, 26.06)	<0.001
Diarrhoea (DI)	−0.99 (−6.75, 4.78)	0.74	−1.31 (−6.82, 4.21)	0.64	1.53 (−4.58, 7.65)	0.62
Financial difficulties (FI)	6.02 (−4.61, 16.65)	0.27	5.19 (−5.62, 16.00)	0.34	2.67 (−9.81, 15.15)	0.67

Abbreviations: CI, confidence interval; EORTC QLQ-C30, European Organisation for Research and Treatment of Cancer Quality of Life Questionnaire Core 30; QoL, quality of life.

Note: Data are expressed as adjusted annualised mean differences (estimates [95% CIs]) across the 16 EORTC QLQ-C30 domains for Phenotype 2 compared with the general-population-mapped reference pool (Phenotypes 1, 4, and 5). Differences represent between-group contrasts in annualised QoL change from baseline to the 12-month follow-up. Interpretation follows the EORTC QLQ-C30 scoring direction. For functional, global health, and summary scores, positive estimates indicate more favourable

change and negative estimates indicate less favourable change relative to the reference pool; for symptom domains, positive estimates indicate greater symptom worsening and negative estimates indicate relative symptom improvement. Sensitivity analyses repeated the core Model 2 after excluding breast cancer survivors, restricting the analysis to women, or restricting the analysis to survivors 5–10 years after diagnosis. In the analysis excluding breast cancer survivors, cancer type remained included as a covariate. In the women-only analysis, sex was omitted by design and smoking status was omitted because it showed no within-stratum variation. In the analysis restricted to survivors 5–10 years after diagnosis, duration of survivorship was omitted because it was fixed by the restriction. All other Model 2 covariates and the corresponding baseline QoL score were retained. Small variations in analytical sample size across QoL domains reflect item-level missingness.

Table S24. Restricted-Sample Sensitivity Analyses of Differences in Annualised Quality-of-Life Change From Baseline to the 12-Month Follow-Up for Phenotype 3 Compared With the General-Population-Mapped Reference Pool

Variables	Phenotype 3 Excluding breast cancer (*N* = 283)	*P* value	Phenotype 3 Women only (*N* = 296)	*P* value	Phenotype 3 Survivorship 5–10 years (*N* = 236)	*P* value
EORTC QLQ-C30 Summary Score	2.02 (−2.24, 6.29)	0.35	2.38 (−1.68, 6.44)	0.25	2.52 (−1.87, 6.91)	0.26
Global Health Status (QL)	3.00 (−7.68, 13.68)	0.58	6.53 (−3.60, 16.65)	0.20	2.90 (−8.01, 13.81)	0.60
Physical functioning (PF)	1.03 (−4.47, 6.54)	0.71	2.03 (−3.36, 7.42)	0.46	1.02 (−5.18, 7.21)	0.75
Role functioning (RF)	2.02 (−3.26, 7.31)	0.45	2.50 (−2.54, 7.54)	0.33	1.14 (−4.66, 6.95)	0.70
Emotional functioning (EF)	4.13 (−1.40, 9.66)	0.14	5.44 (−0.85, 11.72)	0.09	4.88 (−1.87, 11.63)	0.16
Cognitive functioning (CF)	0.13 (−7.49, 7.75)	0.97	2.07 (−5.63, 9.76)	0.60	0.87 (−7.30, 9.04)	0.83
Social functioning (SF)	3.53 (−4.03, 11.09)	0.36	3.91 (−3.42, 11.25)	0.30	2.94 (−5.65, 11.53)	0.50
Fatigue (FA)	−3.01 (−11.18, 5.16)	0.47	0.91 (−7.16, 8.98)	0.82	0.39 (−8.11, 8.89)	0.93
Nausea and vomiting (NV)	−0.51 (−3.99, 2.97)	0.77	0.20 (−2.81, 3.21)	0.90	0.33 (−3.38, 4.03)	0.86
Pain (PA)	−2.57 (−9.32, 4.18)	0.45	−1.45 (−8.08, 5.18)	0.67	−2.64 (−10.13, 4.84)	0.49
Dyspnoea (DY)	−3.11 (−11.13, 4.90)	0.44	−1.73 (−9.32, 5.86)	0.65	−3.34 (−12.10, 5.42)	0.45
Insomnia (SL)	2.94 (−7.42, 13.30)	0.58	−1.63 (−11.97, 8.70)	0.76	−0.98 (−12.27, 10.30)	0.86
Appetite loss (AP)	3.02 (−2.99, 9.03)	0.32	0.96 (−5.37, 7.30)	0.76	3.13 (−3.55, 9.81)	0.36
Constipation (CO)	−3.43 (−11.41, 4.56)	0.40	−2.45 (−10.72, 5.82)	0.56	−7.47 (−16.72, 1.79)	0.11
Diarrhoea (DI)	−4.83 (−10.96, 1.29)	0.12	−4.84 (−10.43, 0.76)	0.09	−5.20 (−11.46, 1.06)	0.10
Financial difficulties (FI)	−1.14 (−12.51, 10.24)	0.84	−1.95 (−12.95, 9.05)	0.73	−2.01 (−14.78, 10.75)	0.76

Abbreviations: CI, confidence interval; EORTC QLQ-C30, European Organisation for Research and Treatment of Cancer Quality of Life Questionnaire Core 30; QoL, quality of life.

Note: Data are expressed as adjusted annualised mean differences (estimates [95% CIs]) across the 16 EORTC QLQ-C30 domains for Phenotype 3 compared with the general-population-mapped reference pool (Phenotypes 1, 4, and 5). Differences represent between-group contrasts in annualised QoL change from baseline to the 12-month follow-up. Interpretation follows the EORTC QLQ-C30 scoring direction. For functional, global health, and summary scores, positive estimates indicate more favourable

change and negative estimates indicate less favourable change relative to the reference pool; for symptom domains, positive estimates indicate greater symptom worsening and negative estimates indicate relative symptom improvement. Sensitivity analyses repeated the core Model 2 after excluding breast cancer survivors, restricting the analysis to women, or restricting the analysis to survivors 5–10 years after diagnosis. In the analysis excluding breast cancer survivors, cancer type remained included as a covariate. In the women-only analysis, sex was omitted by design and smoking status was omitted because it showed no within-stratum variation. In the analysis restricted to survivors 5–10 years after diagnosis, duration of survivorship was omitted because it was fixed by the restriction. All other Model 2 covariates and the corresponding baseline QoL score were retained. Small variations in analytical sample size across QoL domains reflect item-level missingness.

Table S25. Differentially Abundant Whole-Blood Fatty Acids Between Specific Phenotypes and the General-Population-Mapped Reference Pool

Panel A: Phenotype 2			Panel B: Phenotype 3		
**Whole-Blood Fatty Acids**	**Phenotype 2 CLR Difference**	**FDR**	**Whole-Blood Fatty Acids**	**Phenotype 3 CLR Difference**	**FDR**
Pentadecenoic acid (15:1)	−3.50	<0.001	Osbond acid (22:5n-6)	2.06	<0.001
Osbond acid (22:5n-6)	2.14	<0.001	Lignoceric acid (24:0)	−1.13	<0.001
Heptadecenoic acid (17:1)	−1.05	<0.001	Adrenic acid (22:4n-6)	−1.60	<0.001
Lignoceric acid (24:0)	−1.00	<0.001	Margaric acid (17:0)	0.24	<0.001
Adrenic acid (22:4n-6)	−1.53	<0.001	Erucic acid (22:1)	0.63	<0.001
Tricosanoic acid (23:0)	0.65	<0.001	Arachidic acid (20:0)	0.24	<0.001
Pentadecanoic acid (15:0)	0.92	<0.001	Stearic acid (18:0)	0.16	<0.001
Stearic acid (18:0)	0.30	<0.001	Gondoic acid (20:1n-9)	0.25	<0.001
Oleic acid (18:1n-9 *cis*)	0.34	<0.001	Palmitic acid (16:0)	0.15	<0.001
Palmitic acid (16:0)	0.31	<0.001	Oleic acid (18:1n-9 *cis*)	0.17	<0.001
Erucic acid (22:1)	0.99	<0.001	Elaidic acid (18:1n-9 *trans*)	−0.91	<0.001
Margaric acid (17:0)	0.33	<0.001	Tricosanoic acid (23:0)	0.18	<0.001
Linolelaidic acid (18:2n-6 *trans*)	0.26	<0.001	Behenic acid (22:0)	−0.16	<0.001
Nervonic acid (24:1n-9)	0.26	<0.001	Nervonic acid (24:1n-9)	0.15	<0.001
Gondoic acid (20:1n-9)	0.29	<0.001	Pentadecenoic acid (15:1)	0.12	<0.001
Palmitoleic acid (16:1)	0.29	<0.001	Linolelaidic acid (18:2n-6 *trans*)	0.09	<0.001
Arachidic acid (20:0)	0.15	<0.001	Heptadecenoic acid (17:1)	0.11	<0.001
Linoleic acid (LA, 18:2n-6 *cis*)	0.12	<0.001	Docosahexaenoic acid (DHA, 22:6n-3)	−0.17	<0.001
Docosapentaenoic acid (DPA, 22:5n-3)	−0.29	<0.001	Pentadecanoic acid (15:0)	0.04	<0.001
Docosahexaenoic acid (DHA, 22:6n-3)	−0.20	<0.001	Palmitoleic acid (16:1)	0.14	<0.001

Table S25. Differentially Abundant Whole-Blood Fatty Acids Between Specific Phenotypes and the General-Population-Mapped Reference Pool (Continued)

Panel A: Phenotype 2			Panel B: Phenotype 3		
**Whole-Blood Fatty Acids**	**Phenotype 2 CLR Difference**	**FDR**	**Whole-Blood Fatty Acids**	**Phenotype 3 CLR Difference**	**FDR**
Myristic acid (14:0)	0.27	<0.001	Linoleic acid (LA, 18:2n-6 *cis*)	0.07	<0.001
Arachidonic acid (AA, 20:4n-6)	−0.14	<0.001	γ-Linolenic acid (GLA, 18:3n-6)	−0.71	<0.001
Eicosapentaenoic acid (EPA, 20:5n-3)	−0.30	<0.001	Arachidonic acid (AA, 20:4n-6)	−0.07	<0.001
α-Linolenic acid (ALA, 18:3n-3)	0.18	<0.001	α-Linolenic acid (ALA, 18:3n-3)	0.12	<0.001
Eicosadienoic acid (20:2)	0.09	<0.001	Docosapentaenoic acid (DPA, 22:5n-3)	−0.10	<0.001
Behenic acid (22:0)	−0.11	<0.001	Myristic acid (14:0)	−0.10	0.17
γ-Linolenic acid (GLA, 18:3n-6)	−0.03	<0.001	Dihomo-γ-linolenic acid (DGLA, 20:3n-6)	0.03	0.19
Elaidic acid (18:1n-9 *trans*)	0.22	0.06	Eicosapentaenoic acid (EPA, 20:5n-3)	0.01	0.30
Dihomo-γ-linolenic acid (DGLA, 20:3n-6)	0.02	0.11	Eicosadienoic acid (20:2)	−0.01	0.75

Abbreviations: CLR, centred log-ratio; FDR, false discovery rate.

Note: Data are expressed as centred log-ratio (CLR) differences, quantifying the relative abundance of specific whole-blood fatty acids for Phenotype 2 (Panel A) and Phenotype 3 (Panel B) compared with the general-population-mapped reference pool (merged Phenotypes 1, 4, and 5). Positive CLR differences denote relative enrichment of the fatty acid within the specific phenotype compared with the reference pool, whereas negative differences denote relative depletion. Statistical significance for pairwise comparisons was determined via Wilcoxon rank-sum tests. To account for multiple comparisons, P values were adjusted using the Benjamini-Hochberg false discovery rate (FDR) procedure. Variables within each panel are sorted in ascending order of their FDR-adjusted P values.

Figure S1. Participant Selection and Cohort Formation

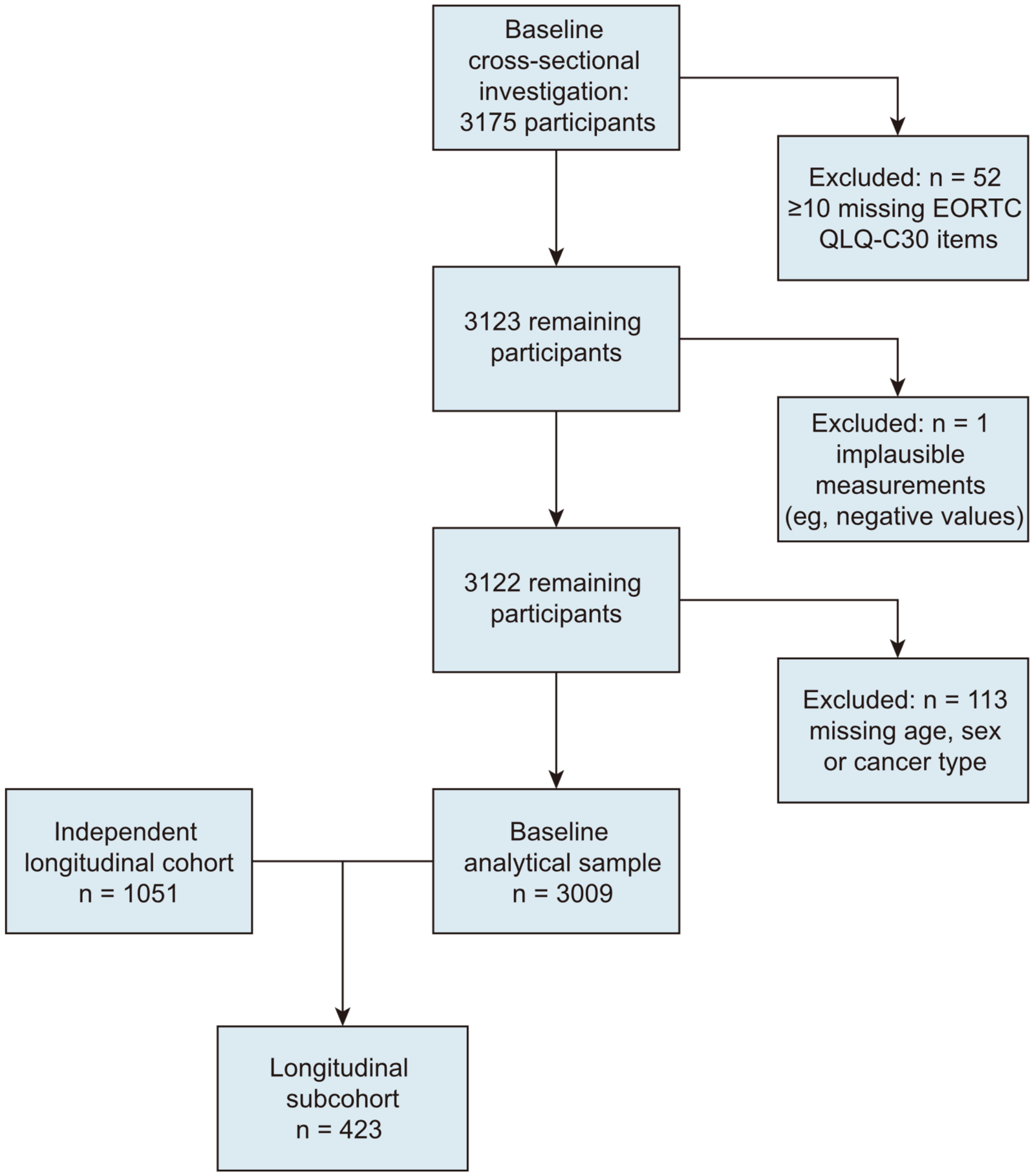

Abbreviations: EORTC QLQ-C30, European Organisation for Research and Treatment of Cancer Quality of Life Questionnaire Core 30.

Note: The final baseline analytical sample comprised 3009 cancer survivors. Cross-linking this baseline analytical sample with an independent longitudinal cohort (*n* = 1051) identified 423 overlapping participants who constituted the longitudinal subcohort. Baseline sociodemographic, lifestyle, and clinical characteristics of the analytical sample are presented in Table 1.

Figure S2. Unsupervised Clustering of Whole-Blood Fatty Acid Profiles Among Cancer Survivors

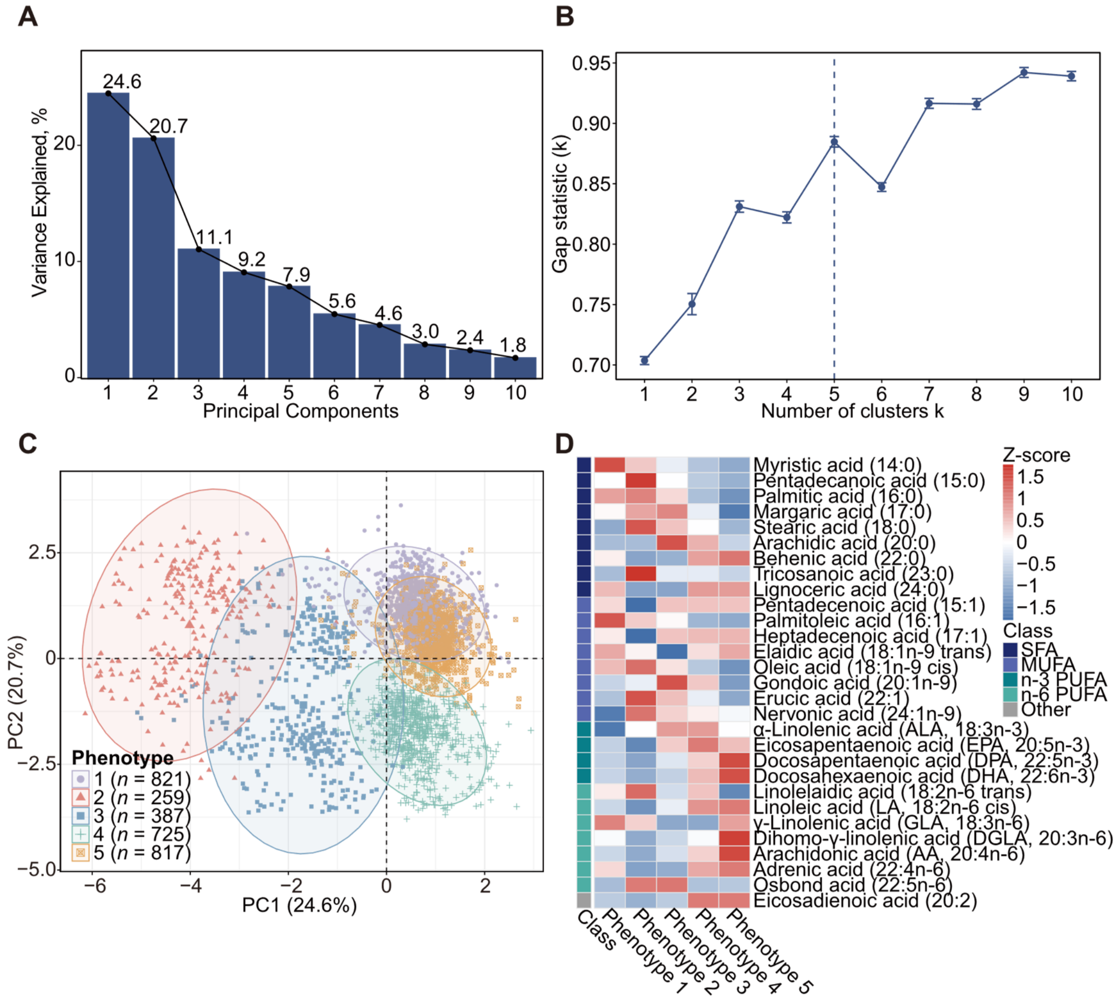

A, Scree plot illustrating the percentage of variance explained by the top 10 principal components following centred log-ratio transformation. **B,** Gap statistic curve used to determine the optimal cluster number, demonstrating a peak at $k = 5$. **C,** Principal component analysis (PCA) score plot visualising the distribution of the 5 whole-blood fatty acid clusters across the first 2 dimensions. Ellipses indicate 95% confidence intervals. **D,** Standardised heatmap depicting the standardised medians (Z scores) of whole-blood fatty acids across the 5 clusters.

Abbreviations: MUFA, monounsaturated fatty acid; PCA, principal component analysis; PUFA, polyunsaturated fatty acid; SFA, saturated fatty acid.

Note: To maintain computational stability during unsupervised dimensionality reduction, variables exhibiting zero values in greater than 50% of the baseline population—specifically heneicosanoic acid (21:0), myristoleic acid (14:1), eicosatrienoic acid (20:3n-3), and docosadienoic acid (22:2n-6)—were excluded from the initial principal component analysis and subsequent clustering algorithms. For the retained fatty acids, exact zero values were replaced with one-half of the minimum nonzero value observed for the corresponding fatty acid before CLR transformation.

Figure S3. Dietary Intake Profiles, Nutritional Balance, and Nutrition Literacy Across Whole-Blood Fatty Acid Phenotypes

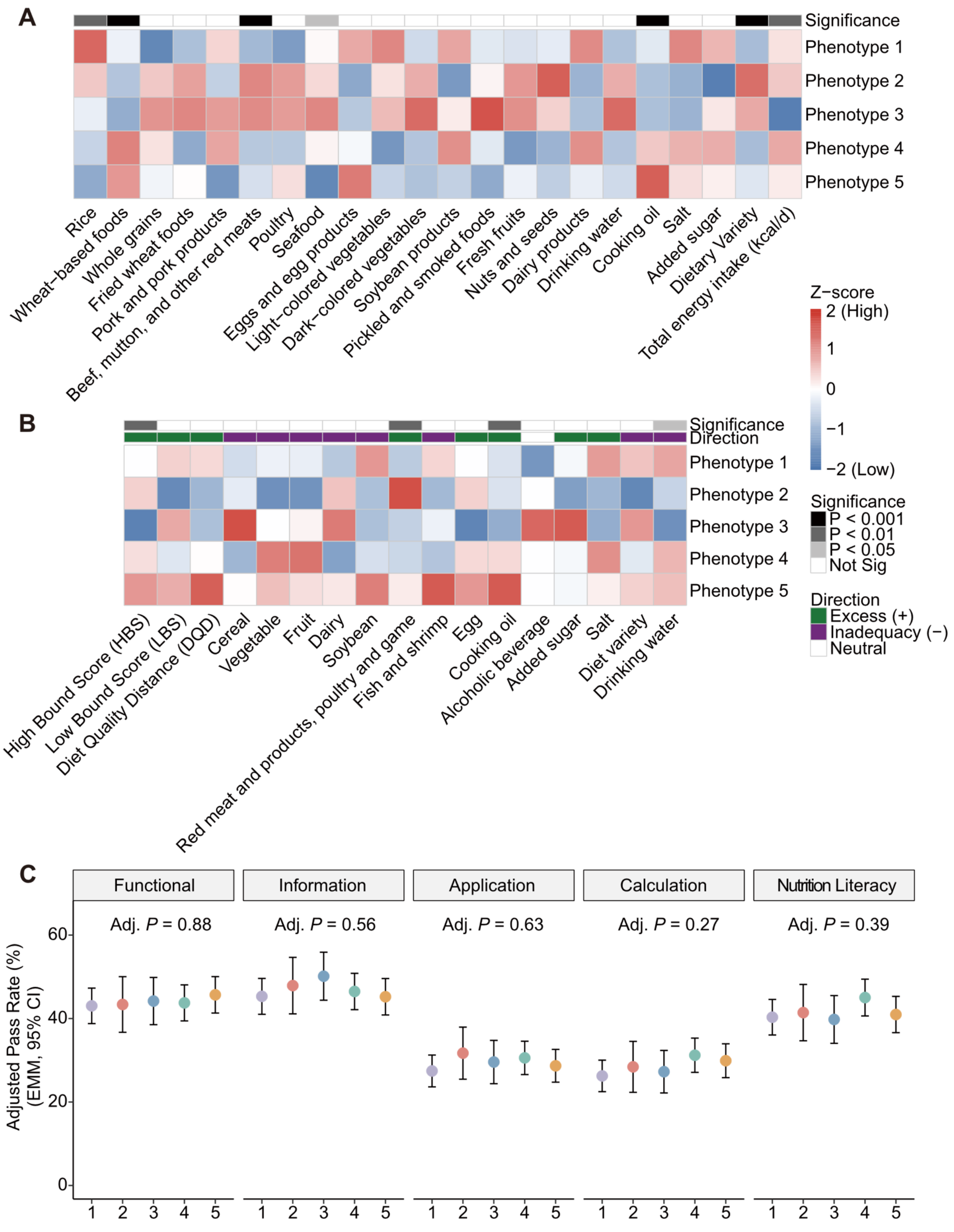

A, Dietary intake profiles, dietary variety, and total energy intake derived from a semiquantitative food frequency questionnaire. **B**, Dietary balance evaluated using the Chinese Dietary Balance Index-22 (DBI-22). **C**, Nutrition literacy performance across 5 evaluation domains.

Abbreviations: Adj. *P*, adjusted P value; CI, confidence interval; DBI-22, Chinese Dietary Balance Index-22; DQD, diet quality distance; EMM, estimated marginal mean; HBS, high bound score; LBS, low bound score.

Note: The analytical sample was restricted to participants with valid dietary assessments and plausible

total energy intakes (*n* = 2812). In A and B, data are visualized as standardised Z-scores of covariate-adjusted estimated marginal means (EMMs) across the 5 phenotypes. In B, to unify the interpretation of dietary balance, DBI-22 scores (including inadequacy and excess indicators) were converted to absolute values prior to standardization; the Direction annotation track specifies whether the original value was positive (indicating dietary excess, green) or negative (indicating dietary inadequacy, purple). In C, point estimates represent covariate-adjusted pass rates (EMMs), and error bars indicate 95% CIs. A hierarchical covariate adjustment strategy was implemented: DBI-22 indices were adjusted for cancer type; total energy and dietary variety were adjusted for age, sex, and cancer type; individual dietary components were further adjusted for total energy. Overall statistical significance across the phenotypes was determined using analysis of covariance (ANCOVA) for dietary profiles (A and B) and Wald $\chi 2$ tests derived from logistic regression models for nutrition literacy pass rates (C). Comprehensive absolute estimates and exact P values for all evaluated variables are detailed in Tables S8–S10.

Figure S4. Differential Whole-Blood Fatty Acid Signatures for Phenotypes 2 and 3 Compared With the General-Population-Mapped Reference Pool

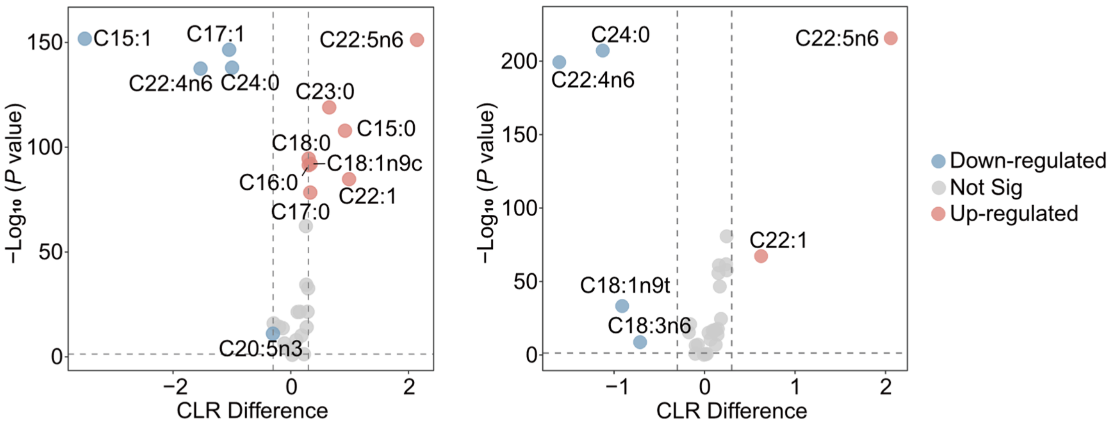

Abbreviations: C15:0, pentadecanoic acid; C15:1, pentadecenoic acid; C16:0, palmitic acid; C17:0, margaric acid; C17:1, heptadecenoic acid; C18:0, stearic acid; C18:1n9c, oleic acid; C18:1n9t, elaidic acid; C18:3n6, γ-linolenic acid; C20:5n3, eicosapentaenoic acid; C22:1, erucic acid; C22:4n6, adrenic acid; C22:5n6, osbond acid; C24:0, lignoceric acid; CLR, centred log-ratio.

Note: Volcano plots depict differentially abundant whole-blood fatty acids for Phenotype 2 (left) and Phenotype 3 (right) relative to the general-population-mapped reference pool (merged Phenotypes 1, 4, and 5). Effect sizes are expressed as CLR differences on the *x*-axis, with statistical significance (−log~10~ *P* values) on the *y*-axis. Significance thresholds for differential abundance were defined as a Wilcoxon rank-sum test $P < .05$ and an absolute CLR difference > 0.3. Red circles indicate relative enrichment, blue circles indicate relative depletion, and gray circles denote nonsignificant variations.
