# Supplementary material for: Whole-Blood Fatty Acid Phenotypes and Subsequent Quality-of-Life Changes Among Cancer Survivors
Source: Nutrients. 2026 Aug 28;18(17):2838. doi: 10.3390/nu18172838 (PMC13567407; doi:10.3390/nu18172838)
Supplement: Supplementary file 1 [file nutrients-18-02838-s001.zip › nutrients-4459442-supplementary/Supplementary_File_S2_Whole_Blood_Fatty_Acid_Phenotype_Calculator.pdf]

Supplementary File S2. Toolkit for the Whole-Blood Fatty Acid Phenotype Calculator

Overview

The complete computational toolkit for the Whole-Blood Fatty Acid Phenotype Calculator (version 1.0.0) has been archived in a dedicated, public GitHub repository. This repository contains the necessary components required to reproduce the phenotype assignment and external projection workflow described in this study.

Access Link

The toolkit is available at the following URL:

https://github.com/lvl000/Whole-Blood-Fatty-Acid-Phenotype-Calculator

Contents of the Repository

1. Phenotype_Calculator.R: The core R script containing the predict_phenotype() function.
2. Anonymous_Phenotype_Core.RData: The serialized parameter file containing the baseline feature list, PCA rotation matrix, cluster centroids, 99% distance rejection boundaries, and the baseline fallback limits for zero-imputation.
3. example_validation_cohort.xlsx: A de-identified sample dataset provided for validation and testing of the calculator's functionality.
4. README.md: Detailed instructions regarding system requirements (R version, necessary packages), input data formatting (e.g., automatic handling of percentage-scale inputs), and execution steps.

Access and Reproducibility

Reviewers can access the source code and example data directly via the link above. For a comprehensive explanation of the mathematical framework, including PCA-based projection and distance-based outlier rejection, please refer to Methods S3.
